# SLFN11 informs on standard of care and novel treatments in a wide range of cancer models

**DOI:** 10.1038/s41416-020-01199-4

**Published:** 2020-12-18

**Authors:** Claudia Winkler, Joshua Armenia, Gemma N. Jones, Luis Tobalina, Matthew J. Sale, Tudor Petreus, Tarrion Baird, Violeta Serra, Anderson T. Wang, Alan Lau, Mathew J. Garnett, Patricia Jaaks, Elizabeth A. Coker, Andrew J. Pierce, Mark J. O’Connor, Elisabetta Leo

**Affiliations:** 1 Bioscience, Oncology R&D, AstraZeneca, Cambridge, UK; 2 Bioinformatics and Data Science, Research and Early Development, Oncology R&D, AstraZeneca, Cambridge, UK; 3 Translational Medicine, Oncology R&D, AstraZeneca, Cambridge, UK; 4 Signalling Programme, The Babraham Institute, Babraham Research Campus, Cambridge, CB22 3AT, Cambridge, UK; 5 Experimental Therapeutics Group, Vall d’ Hebron Institute of Oncology, Barcelona, Spain; 6 Wellcome Sanger Institute, Cambridge, UK

**Keywords:** Cancer imaging, Cancer models, Tumour biomarkers, Breast cancer

## Abstract

**Background:**

Schlafen 11 (SLFN11) has been linked with response to DNA-damaging agents (DDA) and PARP inhibitors. An in-depth understanding of several aspects of its role as a biomarker in cancer is missing, as is a comprehensive analysis of the clinical significance of SLFN11 as a predictive biomarker to DDA and/or DNA damage-response inhibitor (DDRi) therapies.

**Methods:**

We used a multidisciplinary effort combining specific immunohistochemistry, pharmacology tests, anticancer combination therapies and mechanistic studies to assess SLFN11 as a potential biomarker for stratification of patients treated with several DDA and/or DDRi in the preclinical and clinical setting.

**Results:**

SLFN11 protein associated with both preclinical and patient treatment response to DDA, but not to non-DDA or DDRi therapies, such as WEE1 inhibitor or olaparib in breast cancer. SLFN11-low/absent cancers were identified across different tumour types tested. Combinations of DDA with DDRi targeting the replication-stress response (ATR, CHK1 and WEE1) could re-sensitise SLFN11-absent/low cancer models to the DDA treatment and were effective in upper gastrointestinal and genitourinary malignancies.

**Conclusion:**

SLFN11 informs on the standard of care chemotherapy based on DDA and the effect of selected combinations with ATR, WEE1 or CHK1 inhibitor in a wide range of cancer types and models.

## Background

In vitro, independent analyses have identified Schlafen 11 (SLFN11) as the strongest predictor of sensitivity to DNA-damaging agents (DDA), such as topoisomerase I (e.g., irinotecan), topoisomerase II (e.g., etoposide), DNA synthesis inhibitors (e.g., gemcitabine) and DNA cross-linkers and alkylating agents (e.g., cisplatin).^1,2^ In addition to DDA, SLFN11 has been also associated with sensitivity to PARP inhibitors (PARPi),^3–6^ which belong to the class of DNA damage-response inhibitors (DDRi). For the aforementioned agents, and in multiple cancer cell line panels, the presence of SLFN11 has been shown to correlate with sensitivity, whereas the low or absent expression has been associated with resistance.^1,7^ SLFN11 is only expressed in humans and very few other vertebrates^8^, and recent publications highlight SLFN11 bimodal expression in cancer cells.^3–5,9,10^ Furthermore, it has been reported that the expression of SLFN11 is greater in tumours than in adjacent normal tissues^1,11^ and that SLFN11 expression decreases on exposure to chemotherapy.^5,9^ At present, it is unknown whether this bimodal pattern of expression is preserved in tumour tissues and a global assessment of SLFN11 across different cancer types is missing to inform whether SLFN11 may be a relevant biomarker for stratification in cancer patients.

A common downstream effect of DDA in the S-phase of the cell cycle is the slowing or stalling of DNA replication forks, the key characteristic of replication stress.^12,13^ Replication stress in turn triggers the activation of ataxia telangiectasia mutated and Rad3-related (ATR) and checkpoint kinase 1 (CHK1). The ATR-CHK1 signalling pathway helps to halt DNA replication until the replication-stress condition can be relieved and downstream signalling eventually induces a cell cycle arrest. WEE1 kinase is also involved in modulating the replication-stress response.^13^ Accordingly, ATR/WEE1 and CHK1 inhibitors have been associated with the induction of replication stress, DNA damage and cell death in specific cancer types.^14,15^

A connection between SLFN11 and replication stress has been recently reported.^3,7,16,17^ Following S-phase DNA damage and replication stress, SLFN11 has been shown to bind to replication forks^16^ where it blocks replication independently of ATR^7^ and induces the expression of early response genes.^17^ In SLFN11-negative settings, combinations of ATR inhibitor (ATRi) with topoisomerase I inhibitors^7^ or PARPi^3^ have been shown to reverse resistance to the monotherapy by relieving the S-phase checkpoint. Accordingly, this sensitisation was also described for the combination of irinotecan and trabectedin with ATR inhibitor in PDX models.^18,19^ Whether low or absent SLFN11 cancers may be re-sensitised to chemotherapy with combinations of other DDRi, particularly those that also modulate the replication stress response, is currently unknown as an in-depth analysis of whether SLFN11 is associated with response to DDRi monotherapies other than PARPi.

So far, quantification of SLFN11 has mainly been performed by transcript levels in cancer,^1,20,21^ with some protein assessment by IHC in lung,^4,5,9,22^ breast^19^ and colorectal cancer (CRC).^23^ Thus, a broad understanding of the correlation between SLFN11 by transcript and protein levels beyond these indications is still missing, as is a comprehensive analysis of the clinical significance of SLFN11 as a predictive marker to DDA and DDRi therapies.

In this study, in a multidisciplinary effort, we aimed to gain insights into the role of SLFN11 as a biomarker for sensitivity to DDA and DDRi therapies, in preclinical and clinical settings. We evaluated SLFN11 in different cancer types and correlated it with drug and patient treatment in two different breast cancer cohorts. We explored novel sensitisation strategies for DDA monotherapies in SLFN11-absent settings by combining DDA with DDRi and examined the applicability of these combinations to overcome drug resistance caused by low or absent SLFN11 protein in different cancer types.

## Methods

### Cell culture and compounds

DU145 cells were cultured in EMEM media (ATCC) and HT29 cells in McCoy’s 5A modified medium (Sigma) supplemented with 10% FBS (Sigma). In the pancreatic cell line panel, ASPC-1, BXPC3, KP-4, PSN1 and YAPC were cultured in RPMI 1640 media plus 10% FBS; CAPAN-1 and CFPAC-1 cells in IMDM media plus 20% FBS and 10% FBS, respectively; CAPAN-2 cells in McCoy’s 5A media plus 10% FBS and HPAF-II, HS-766T, HUP-T4, MiA-PACA-2 in DMEM media plus 10% FBS. PANC-02.03, PANC-03.27, PANC-04.03, PANC-08.13 and PANC-10.05 cells were grown in RPMI 1640 media plus 15% FBS and 1 U/ml insulin and HPAC cells in 1:1 DMEM/Hams’ F12 medium mix, 5% FBS, 0.002 mg/ml insulin, 0.005 mg/ml transferrin, 40 ng/ml hydrocortisone and 10 ng/ml EGF. All pancreatic cell line media was additionally supplemented with penicillin, streptomycin and 2 mM glutamine (all from GIBCO); insulin, transferrin, hydrocortisone and EGF were from Sigma. All cell lines were authenticated by short tandem repeat (STR) DNA fingerprinting analysis and validated free of *Mycoplasma* and virus, as previously described.^14^ Ceralasertib (AZD6738),^24^ adavosertib (AZD1775),^25^ AZD7648,^26^ AZD0156,^27^ AZ-31, AZD6244, prexasertib,^28^ SRA737 and olaparib were synthesised at AstraZeneca. Gemcitabine, cisplatin, hydroxyurea (HU) and etoposide were obtained from Tocris, camptothecin from Sigma, and GDC-0623 from Cayman Chemicals. Stock solutions of gemcitabine (50 mM), cisplatin (1.67 mM) and HU (1 M) were prepared in an aqueous solution; all other drugs were dissolved at 10 mM concentration in DMSO. In total, 10 mM SN-38 dissolved in DMSO was obtained from Abcam (ab141108). The experiment to verify SLFN11 protein levels following continuous chemotherapy treatment was performed similarly as previously described.^29^ Briefly, DU145 cells were plated in T75 flasks (0 h time point). Twenty-four hours later, treatment with DMSO or SN-38 at final concentrations of 1 and 4 nM was initiated based on reported median SN-38 plasma concentrations circulating in patients for up to 3 weeks after infusion of the standard dose of irinotecan.^29^ Every 3 till 4 days cells were split, and an aliquot was taken for immunoblotting. Every 24–48 h, the medium was refreshed to minimise potential confounding effects deriving from SN-38 chemical instability. Due to cytotoxicity effects, for the 4 nM dose aliquots were only taken at day 3 and 20. Knockout of SLFN11 was performed by CRISPR/Cas9 in-house. Transient knockdown of SLFN11 was performed by siRNA transfections using RNAi-Max kit (Thermofisher Scientific), as previously described.^1^

### Combination synergy and correlation analysis

Combination activity (synergism) was performed and calculated using the HSA dose-additivity model in Genedata Screener software. For correlation analysis of SLFN11 RMA normalised gene expression with drug response (log(IC_50_)), mutational burden, copy number variations or ploidy data sets were downloaded from GDSC database.^30^ To test response to various DDA or DDRi monotherapies or to determine SLFN11 mRNA expression in cell lines, Sanger pharmacology monotherapy data (IC_50_ values)^30^ and SLFN11 expression data were retrieved from publicly available and unpublished data from GDSC and AstraZeneca. SN-38 drug combination data were taken from an in-house database of unpublished collaboration data between Sanger and AstraZeneca. Cell lines with HSA values (excess effect over Highest Single Agent) greater than 0.1 and maximum activity of the combination over 0.5 were considered to benefit from the combination treatment. CCLE SLFN11 RNA-Seq expression data from the DepMap Consortium 20Q1 release were used to divide the cell lines into SLFN11 high and low groups. We fitted a mixture of Gaussians model to the RNA-Seq expression data to try to determine the best threshold to define the groups, but since a significantly better fit was obtained by using more than two components, a range of threshold values were considered to be equally valid. Cells with log2(TMP + 1) values greater than 2 were classified as SLFN11 high and the rest as SLFN11 low, but similar results were obtained with a threshold of 0.5. A summary of all the combined data can be found in Supplementary Table S1.

### CellTiter-Glo viability assays, clonogenic assay and IncuCyte time-lapse imaging

For CellTiter-Glo luminescent assays (Promega), cells in 96-well plates were compound dosed using a HP dispenser or manually dosed, and cell viability was determined 72 h later. Spheroids were formed with fibroblasts, as previously described.^31^ After 3 or 4 days, formed spheroids were compound dosed using HP dispenser and 72 h later, cell viability determined by 3D-cell titre Glo assay (Promega). Percentage growth for both 2D and 3D cultures was determined using the equation (*T*–*T*0)/(C–*T*0) × 100, where *T* = Compound-treated cells/spheroids; *T*0 = cells/spheroids at 0 h time point and C = control cells/spheroids. Clonogenic assays and IncuCyte time-lapse imaging were performed as previously described.^32^

### Immunostaining and immunoblotting

For the high-content imaging assay, cells were incubated with 10 µM (5-ethynyl-2’-deoxyuridine) EDU (Thermo Fisher Scientific) prior fixation. EDU Click-iT reactions were performed in a buffer containing 100 mM Tris-HCl pH 7.5, 2 mM CuSO_4_, 100 mM ascorbate and 5 µM Alexa Fluor 647 azide (Thermo Fisher Scientific) for 30 min, followed by several washes in 1% BSA–PBS and immunostaining. For immunostaining, cells were fixed and permeabilised with 4% paraformaldehyde (Affymetrix) and 0.35% Triton X-100 (Sigma), blocked in 3% BSA–PBS and incubated with primary antibodies, followed by secondary Alexa-Flour conjugated antibodies for 1 h and counterstaining with DAPI. Confocal images were acquired with a Cell Voyager 7000 spinning disk confocal microscope (Yokogawa) using the ×40 or ×60 objective. Image analysis was performed on a Columbus™ image data storage and analysis system (Perkin Elmer) using optimised image algorithms to identify the response of DNA damage and replication stress response markers. For immunoblotting, cells were lysed with CelLyticTM lysis reagent (Sigma),^3^ or whole-cell extracts were obtained as described^32^ or by direct lysis in Laemmli sample buffer (Bio Rad). Subcellular fractions were prepared using the Subcellular Protein Fractionation Kit (Thermo Fisher Scientific). GAPDH served as a loading control for the cytoplasmic fraction, and H3 as a loading control of the chromatin-bound fraction. Cell lysates were analysed by standard SDS-PAGE immunoblotting. The used antibodies for immunoblotting and immunofluorescence are summarised in Supplementary Table S2.

### PDX models

Patient-derived multi-tumour tissue microarrays (TMAs, TMA 17–20 and 22–24) were obtained from Champions Oncology and associated patient/tumour profiles, patient treatment information and PDX drug sensitivity data were retrieved from Champions TumorGraft® database (https://database.championsoncology.com). For the clinical dataset, we assessed SLFN11 protein in the grafts and retrospectively correlated it with patient treatment outcome information, available from Champions TumorGraft® database. The optimal SLFN11 H-score cut-off to predict response (TGI >50) was derived using the OptimalCutpoints R package with the Youden method. TNBC and two ovarian PDX models were generated in VHIO (Spain). These models were drug-treated, RNA sequenced and response analysed, as described.^33,34^ Briefly, those models were treated with either olaparib at 50 mg/kg QD continuous, WEE1 inhibitor adavosertib (AZD1775) at 60 and 120 mg/kg 5 days on/9 days off, or a combination of olaparib and WEE1i (for this combination, continuous 50 mg/kg QD olaparib plus the aforementioned dosing and scheduling of WEE1i). All experiments were performed in accordance with relevant guidelines and regulations. Information regarding the institutional and/or licensing committee approving the experiments, informed consent and mouse handling can be found in Cruz C. et al., 2018^34^ and Castroviejo-Bermejo M. et al.^33^ Mouse handling was performed under SPF conditions. Mice were euthanised when tumours reached 1500 mm^3^ or in case of severe weight loss or necrosis, in accordance with institutional guidelines.^33,34^

### Immunohistochemistry and image analysis

SLFN11 IHC was performed on 4-µm thick sections of formalin-fixed paraffin-embedded cell blocks or tissues and carried out on Bond RX (Leica Microsystem) using ER1 (pH 6, Leica) antigen retrieval. Slides from cell and xenograft tissue were stained with primary rabbit polyclonal anti-SLFN11 antibody (Supplementary Table S2). Digital slide image acquisition and HALO (Indica Labs) image analysis were performed, as previously described.^35^ Each PDX model was represented by two cores, and the average H-score was calculated. The same algorithm was used across all PDX models.

### Statistical analysis

A two-tailed paired Student’s *t* test or Wilcoxon test was used to determine statistical differences between two groups of the data, whereas a one-way ANOVA with Dunnett’s T3 multiple comparisons test was used to calculate statistical differences between more groups of the data, as indicated, and is denoted as *P < 0.05; **P < 0.01; ***P < 0.001; ****P < 0.0001; ns, not significant (*P* > 0.05) (GraphPad Prism V8). A two-sided Fisher’s exact test was used to assess the statistical difference between the number of cell lines classified as benefiting from the SN-38/DDRi drug combinations in SLFN11 high versus low groups (fisher.test function in R version 3.6.0).

## Results

### In vitro SLFN11 correlates with response to DDA and some DDRi monotherapies

**Fig. 1:**
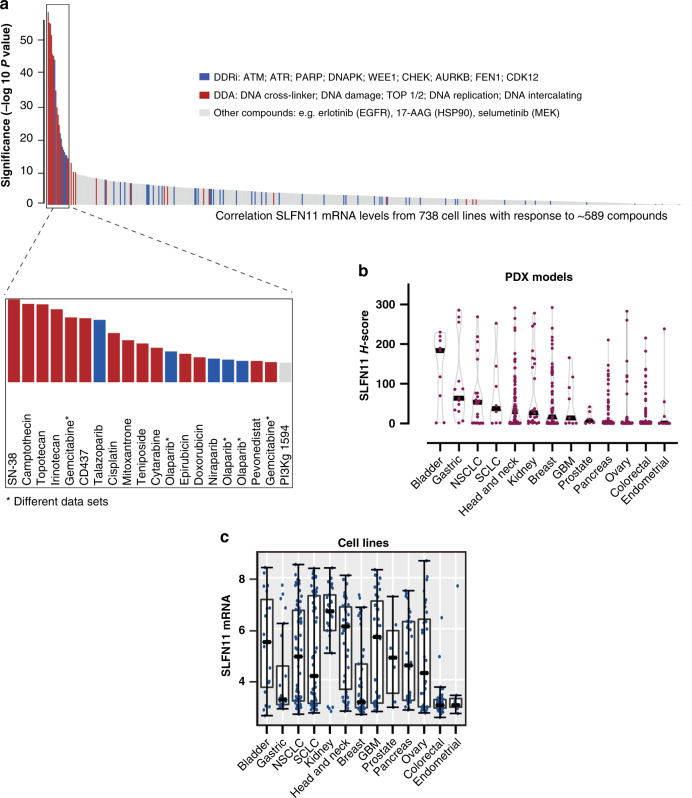
In vitro SLFN11 correlates with response to DDA and some DDRi. SLFN11 low or absent cancers are found across different tumour indications. **a** Correlation of SLFN11 mRNA levels (RMA normalised gene expression) from 738 cell lines with the response to ~589 monotherapy anticancer therapeutics in a multidisciplinary effort from GDSC and AstraZeneca. GDSC genomics of drug sensitivity in cancer. The inset shows the 20 most significantly correlated compounds with SLFN11. **b** SLFN11 protein in Champions Oncology Patient-Derived Xenograft (PDX) models of the different cancer types as determined by IHC assay (*N* = 472). **c** SLFN11 RMA normalised gene expression in cell lines from GDSC grouped by tissue of origin. The bars represent medians ± s.d.

To independently validate the role of SLFN11 as potential biomarker, SLFN11 mRNA expression from 738 cell lines was correlated with the response to ~589 compounds in publicly available data from genomics of drug sensitivity in cancer (GDSC) and AstraZeneca (Fig. 1a). In line with previous reports,^1,3,36^ we found different DDA and some DDRi such as the PARPi talazoparib and olaparib (at µM concentrations) among the most significantly correlated drugs (inset in Fig. 1a). Less significant correlations were found with other PARPi (e.g., veliparib, which inhibits PARP, but does not “trap” it on the DNA), with other DDR inhibitors such as ATM or DNA-PK inhibitor, as well as all other classes of targeted therapies (Fig. 1a).

In the GDSC panel of cell lines, no correlation between SLFN11 DNA copy number alteration and the response to DDA was found (Supplementary Fig. S1A). Mutations in SLFN11 gene were found with low frequency in ~ 6% (*n* = 51) of cell lines, with five cell lines presenting more than a single mutation (Supplementary Fig. S1B and Supplementary Table S3). Half of the mutant cell lines showed low SLFN11 expression (Robust Multichip Average (RMA)—normalised gene expression <5). This included those with missense mutations and not all the truncating variants showed lower mRNA expression. Furthermore, SLFN11 was not correlated with ploidy or mutational burden in cancer (Supplementary Fig. S1C). Thus, we conclude that SLFN11 expression (but not its genetic alterations) is the major determinant correlating with response to DDA and DDRi treatment.

To validate the specificity of an in-house developed IHC assay as well as to validate the correlation between SLFN11 and the response to different DDA and DDRi, SLFN11 knockout (KO) cells were generated in DU145 prostate cancer cells using CRISPR/Cas9 (Supplementary Fig. S1D; Supplementary Fig. S1E, F confirm the KO by western blotting and immunofluorescence, respectively). After subcellular fractionation, SLFN11 was mainly found in the soluble nuclear- and chromatin-bound fraction (Supplementary Fig. S1G). The nuclear SLFN11 localisation was confirmed by IHC (Supplementary Fig. S1D highlights antibody binding to the N-terminal domain (AA 255–333) in SLFN11), but not in SLFN11-absent colorectal HT29 or SLFN11 KO cells (Supplementary Fig. S1H), and less in cells were SLFN11 has been downregulated by siRNA (KD, Supplementary Fig. S1I), validating the sensitivity and accuracy of our IHC assay. Upon permanent (KO) or transient downregulation (KD) of SLFN11, cells were found resistant to several DDA as reported (Supplementary Fig. S2A, B), but less to different DDRi monotherapies. Accordingly, modest resistance could be seen for ATR inhibitor (ATRi) AZD6738 (ceralasertib) and CHK1/2 inhibitor (CHKi) prexasertib, but not for ATM inhibitor (ATMi) AZD0156, WEE1 inhibitor (WEE1i) AZD1775 (adavosertib) (Supplementary Fig. S2C, D) or olaparib (Supplementary Fig. S2E-G).

### SLFN11 associates with both preclinical and clinical response to DDA therapies in breast cancer

Our next goal was to determine SLFN11 transcript and protein levels in different cancer models. SLFN11 protein was assessed with our IHC assay in 472 PDX models from different cancer types (Fig. 1b). The data were compared to transcript levels in cell lines grouped by the same cancer tissue of origin (Fig. 1c). Interestingly, we found varied SLFN11 transcript and protein levels in the different cancer types and noted some concordance between transcript levels in cell lines and protein levels in PDX models (Fig. 1b, c). However, despite the largely observed and described bimodal expression in cell lines,^4,37^ we only observed a bimodal pattern of SLFN11 protein in some, but not all PDX’s. We also found SLFN11 absent/low tumours across the different cancer types tested (Fig. 1b).

**Fig. 2:**
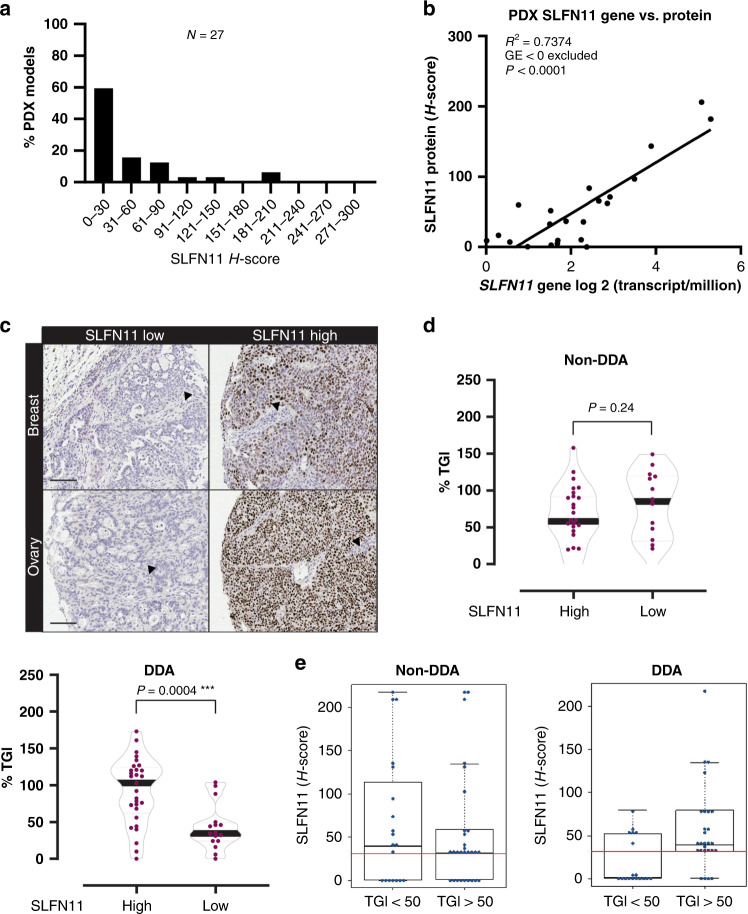
SLFN11 associates with both preclinical and clinical response to DDA therapies in breast cancer. SLFN11 low cancers are less responsive to DDA treatment. **a** Frequency distribution of SLFN11 H-scores in the analysed breast/ovarian cancer cohort. **b** Pearson’s correlation between SLFN11 gene (RNA-sequencing) and protein (H-score) expression. *R* = 0.7374; *P* < 0.0001. **c** Representative images of nuclear SLFN11 determined by IHC in breast and ovarian PDX models. Left, SLFN11 low and right, SLFN11 high, for the indicated cancers. The arrows indicate mice stromal and endothelial cells that are SLFN11 negative. Scale bars, 100 µm. **d** Violin plots representing median % TGI for non-DDA and DDA monotherapy treatment in SLFN11 low (<31 H-score) and high (>31 H-score) breast cancer PDX’s (Wilcoxon test). **e** Box plots depicting median SLFN11 H-scores for non-DDA and DDA treatment in responding (%TGI > 50) and non-responding (%TGI < 50) breast cancer PDX’s. The red continuous line represents the cut point of 31 SLFN11 H-score.

We next sought to validate the correlation between SLFN11 and the response to different DDRi and DDA in vivo. Thereby, we focused on two different breast cancer cohorts treated with DDRi, DDA or non-DDA-based chemotherapy. We first tested a cohort (*N* = 27) mainly composed of triple-negative breast cancer (TNBC).^33,34^ Figure 2a shows the frequency distribution of SLFN11 across the PDXs, indicating a non-bimodal pattern of expression. SLFN11 transcript and protein levels were significantly correlated in this cohort (Fig. 2b; *R*^2^ = 0.7374; *P* < 0.0001), demonstrating that transcript and protein assessment equally reflects SLFN11 levels in PDX tissues. Representative images of grafts demonstrated nuclear SLFN11 localisation in cancer cells, but not mouse-derived stromal and endothelial cells, consistent with the absent expression of SLFN11 in mouse^8^ (Fig. 2c). These PDXs were treated with DDRi monotherapy or combination treatment (olaparib and WEE1i). In analogy with our in vitro results, no significant association between SLFN11 and olaparib or WEE1i single-agent activity, or their combination was found (Supplementary Fig. S3A). Moreover, for these treatments, SLFN11 median did not significantly differ in the responder (CR/PR) and non-responder (SD/PD) groups (Supplementary Fig. S3B).

We next assessed SLFN11 protein by IHC in a second PDX cohort of breast cancers (*N* = 68), where patients had mainly received DDA and non-DDA-based chemotherapy treatment. For this cohort, patients’ clinical and histopathological features are available and summarised in Table 1. Interestingly, SLFN11 was significantly elevated in metastatic cancer (*P* = 0.04, Wilcoxon test), when compared to the primary cancer group. The frequency distribution in this cohort was similar to the one described earlier (Supplementary Fig. S3C). In the preclinical dataset (Supplementary Table S4 for treatment and response of PDXs) an H-score of 31 was defined as a good cut point to divide the PDX models into high and low SLFN11 subgroups (*P* < 0.0001, Wilcoxon test). With non-DDA monotherapies (Fig. 2d and Supplementary Table S4), no significant differences in median %TGI (tumour growth inhibition) were found between the two groups. By contrast, %TGI was significantly elevated in SLFN11-high PDXs treated with DDA monotherapies compared to the SLFN11-low subgroup (*P* = 0.0004, Wilcoxon test). We also classified the PDXs into responders (%TGI > 50%) or non-responders and evaluated whether SLFN11 H-scores can predict response. Indeed, with DDA, a cut point of 31 SLFN11 H-score had good predictive power with 85% sensitivity (AUC 0.74), whereas it did not predict response to non-DDA treatment (AUC 0.50, Fig. 2e). These results were translatable when the clinical responses were considered (Supplementary Fig. S3D): in patients that received DDA-based therapies, responders had higher median SLFN11 compared to those who did not respond (Supplementary Table S5 for treatment and response outcome of patients). Taken together, these findings indicate that SLFN11 is correlated with both preclinical and clinical response to DDA therapies in breast cancer and that SLFN11 low or absent tumours are less responsive to DDA treatment.

**Table 1:** Histopathological and clinical features of 66 breast cancer.

Characteristic	*N*	%	Median SLFN11 H-score	Statistics
*Type of graft*				
Primary BC	32	48	2	ns, *P* = 0.04* (Wilcoxon test)
Metastatic BC	34	52	46	
*Histology*				
Breast carcinoma NOS	21	32	4	ns, (one-way ANOVA with multiple comparison test)
Breast ductal carcinoma	39	59	36	
Breast intraductal carcinoma	4	6	15	
Metaplastic carcinoma	2	3	8	#
*Disease stage*				
I	1	2	34	ns, (one-way ANOVA with multiple comparison test)
II	11	17	28	
III	15	23	31	
IV	24	36	46	
N/A	15	23		
*Diagnosis*				
First diagnosis	17	26	37	ns, *P* = 0.1 (Wilcoxon test)
Recurrent	26	39	22	
N/A	23	35		
*Treatment history*				
Naive	12	18	24	ns, *P* = 0.9 (Wilcoxon test)
Pre-treated	43	65	27	
N/A	11	17		
*ER/PR/HER2 status*				
ER + /PR + /HER2− (luminal A-like)	6	9	24	ns, (one-way ANOVA with multiple comparison test)
ER−/PR−/HER2 + (HER2 “enriched)	5	8	1	
ER−/PR−/HER2− (TNBC)	39	59	5	
ER + /HER2 + (luminal B-like)	2	3	0	#
N/A	14	21		
*Mean age (*N *=* *60)*	52			

*BC* breast cancer, *NOS* not otherwise specified, *N/A* not applicable, *s* significant, *ns* not significant, # not included in statistical testing as *N* = < 3.

### Resistance to broad DDA due to absent SLFN11 can be reversed by combination with ATRi, WEE1i or CHK1i

**Fig. 3:**
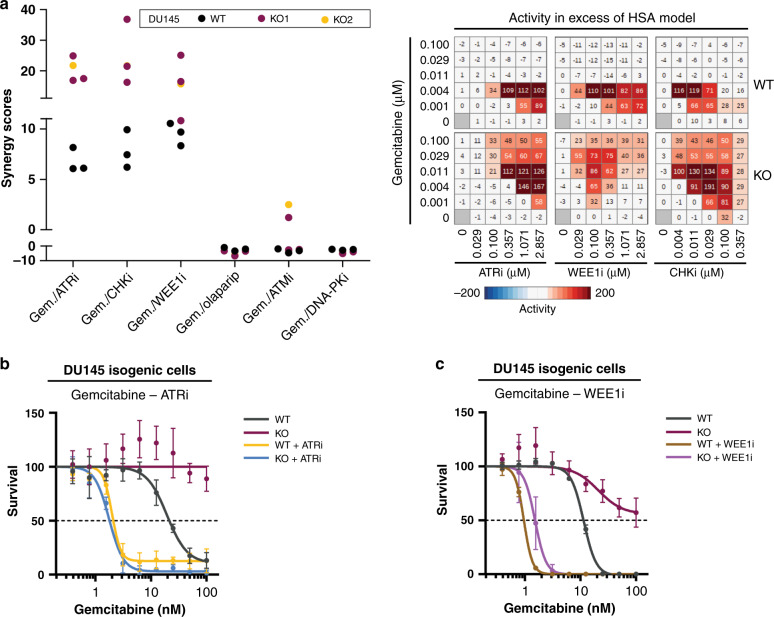
Resistance to DDA due to absent SLFN11 can be reversed by inhibition of ATR, WEE1 and CHK, but not other DDRi. **a** Left, HSA synergy scores of the tested gemcitabine-DDRi combinations in DU145 isogenic cells with the indicated drugs (in triplicates for WT and KO1; *n* = 1 for KO2). Right, representative heatmaps of combination activity in excess of the calculated HSA model. Prexasertib is the CHKi. **b**, **c** Response to gemcitabine in DU145 isogenic cells in the absence or presence of 1 µM ATRi (**b**) and 0.36 µM WEE1i (**c**) (continuous treatment for 72 h) as determined by CellTiter-Glo^®^ luminescent cell viability assays (*n* = 3). Data are presented as mean percentages ± s.d. (*n* = 3) of the DMSO and single-agent DDRi-treated conditions.

We aimed to investigate rationales to overcome resistance observed in SLFN11-negative models to different DDA. We started from gemcitabine, a nucleoside analogue that acts primarily during the S-phase of the cell cycle.^32^ The DU145 isogenic cell line pairs were used to test the response of gemcitabine combinations with each of the following DDRi: AZD0156 (ATMi), AZD1775 (WEE1i), AZD6738 (ATRi), AZD7648 (DNA-PKi), prexasertib (CHK inhibitor) and olaparib (PARPi) (Fig. 3a and Supplementary Fig. S4A). The degree of growth inhibition induced by the combinations and potential synergy (HSA synergy scores) were calculated with Genedata software. Interestingly, the combinations of gemcitabine with WEE1i, ATRi or CHK1i were found more synergistic, hence more effective, than the respective monotherapies in the SLFN11-negative setting. Similar results were confirmed in different assay formats and with broad DDA. Accordingly, SLFN11 KO cells were more resistant (over tenfold IC_50_ increase) to gemcitabine treatment and could be re-sensitised upon combinations with ATRi or WEE1i (Fig. 3b, c). Similar results were observed for etoposide, camptothecin, hydroxyurea or cisplatin. Thereby, SLFN11-deficient cells were resistant to all four DDA and could be re-sensitised through combinations with ATRi (Supplementary Fig. S4B, C), WEE1i (Supplementary Fig. S4B, D) or CHKi (Supplementary Fig. S4B, E). In contrast, no re-sensitisation was observed with combinations with other DDRi (Fig. 3a and Supplementary Fig. S4A, B), for a representative combination of ATMi with ETP or CPT (Supplementary Fig. S4F), implicating the specific relevance of the replication stress ATR/CHK1/WEE1 axis. Of note, the monotherapy treatments with ATRi (0.5–1 µM), WEE1i (0.36 µM) or CHK1i (0.005 µM) had no differential effects in wild-type versus SLFN11-deficient cells (Supplementary Fig. S2C, D) and the doses of DDA and DDRi, for which we observed benefit, are clinically relevant and used in different clinical trials.^38–41^ The combination results could be also confirmed by live-cell imaging (Supplementary Fig. S5A) and in 3D-cell assays (Supplementary Fig. S5B, C). Interestingly, we observed that effects, although still significant, were less enhanced with cisplatin and etoposide, DDA that induce DNA double-strand breaks (DSBs) also in phases of the cell cycle other than S-phase.

### SLFN11 protein levels are not decreased following chemotherapy treatment

**Fig. 4:**
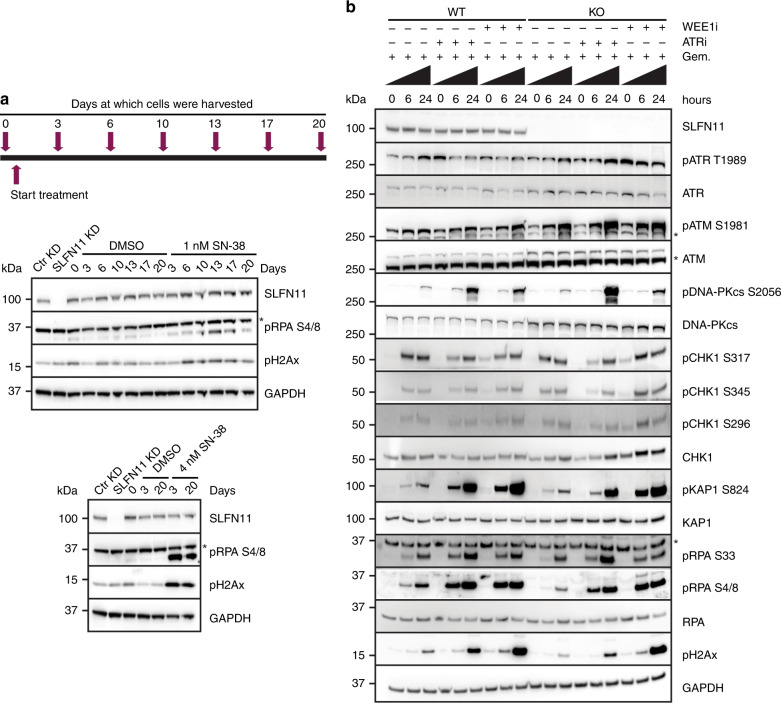
SLFN11 protein levels are not decreased following chemotherapy treatment; ATR or WEE1 inhibition restores DNA damage and replication stress in SLFN11-deficient cells. **a** Scheme for the protocol used to study SLFN11 protein evolvement following treatment with SN-38 in DU145 wild-type cells as further specified in “Methods”. Also shown are immunoblot analyses of SLFN11 and the indicated biomarkers. *, signal derives from a reprobing of GAPDH blots with pRPA S4/8. **b** Representative immunoblots of SLFN11 and the indicated biomarkers in DU145 isogenic pair treated for the indicated hours with gemcitabine monotherapy or gemcitabine/ATRi/WEE1i combinations. Similar results were obtained in a second, independent, experiment. *, aspecific bands.

We next sought to determine whether SLFN11 levels, as proposed by others,^5,9^ are decreased following chemotherapy treatment. To test this hypothesis, SLFN11-proficient cells were treated with clinically relevant doses of SN-38 (as described in M&M and by Mathijssen et al.^29^). Unexpectedly, we found no changes in SLFN11 protein levels with 1 nM or 4 nM SN-38 treatment over the three-week time period (Fig. 4a), although the treatments induced DNA damage (pH2AX) and replication stress (pRPA). In summary, these results show that SLFN11 protein levels do not decrease following chemotherapy treatment under the conditions tested.

### ATR or WEE1 inhibition induces DNA damage and replication stress in DDA-treated SLFN11-deficient cells

**Fig. 5:**
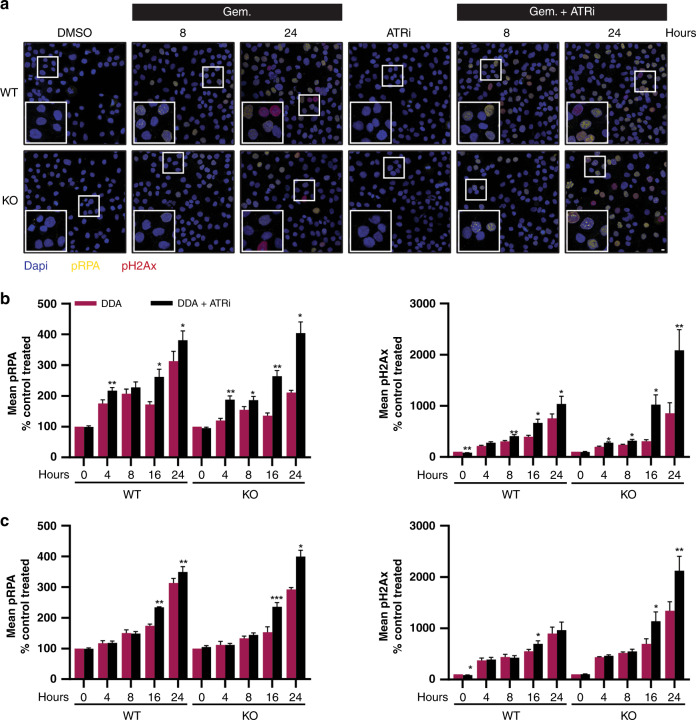
ATR or WEE1 inhibition induces DNA damage and replication stress in SLFN11-deficient gemcitabine or etoposide-treated cells. **a** Staining for DNA (DAPI), pRPA (S4/8) and pH2Ax in DU145 isogenic cells treated for the indicated time points with the designated compounds. The insets show a ×2 magnification. Scale bar, 10 µm. **b**, **c** pRPA (S4/8) and pH2Ax mean intensities in DU145 isogenic cells treated with gemcitabine, ATRi (**b**) or etoposide, ATRi (**c**) for the indicated time points. The data are presented as mean ± s.e.m. (*n* = 4) of the control-treated condition. *P < 0.05; **P < 0.01; ***P < 0.001 (paired Student’s *t* test).

We investigated the mechanism of re-sensitisation to broad DDA by inhibition of ATR or WEE1 in SLFN11-absent setting. We first analysed the DNA damage signalling and replication stress following gemcitabine, ATRi/WEE1i treatment by western blotting (Fig. 4b). In WT cells, ATRi and WEE1i abrogated the gemcitabine induced checkpoint activation, as visualised by decreased phosphorylation levels of ATR T1989 and CHK1 S317, S345 and S396 (at early 6-h time point). The phosphorylation of these sites was more pronounced in the KO cells, especially after WEE1i-gemcitabine treatments (Fig. 4b and Supplementary Fig. S6). ATR and WEE1 inhibition elicited compensatory pathway activation, as demonstrated by increased ATM S1981 and DNA-PK S2056 autophosphorylation and downstream signalling (increased KAP1 S824 phosphorylation,). We noted lower levels of pRPA (S4/8 and S33) and pH2Ax in KO cells following gemcitabine monotherapy treatment and the combinations restored these levels comparable to those in WT cells. We also analysed the cellular response to broad DDA (gemcitabine, etoposide and cisplatin) monotherapy or combination treatment with ATRi by high-content microscopy imaging. ATRi monotherapy treatment had no phenotypical effect in cells (Fig. 5a). We noted higher EdU incorporation in KO cells, which was further elevated by ATR inhibition, consistent with fork progression and an intra-S checkpoint override (Supplementary Fig. S7A, B). Combination treatment induced the accumulation of DNA damage (pH2Ax) and replication stress (pRPA) in SLFN11-deficient cells (Fig. 5b, c and Supplementary Fig. S7B). Finally, the combinations induced cytotoxicity in KO cells as observed by time-lapse imaging (Supplementary Fig. S5A). Taken together, these results suggest that combination treatment induces replication stress, DNA damage and subsequently cell death in SLFN11-deficient/absent cells.

### Resistance to DDA due to low or absent SLFN11 can be overcome by ATRi, WEE1i or CHK1i combinations in different cancer types

**Fig. 6:**
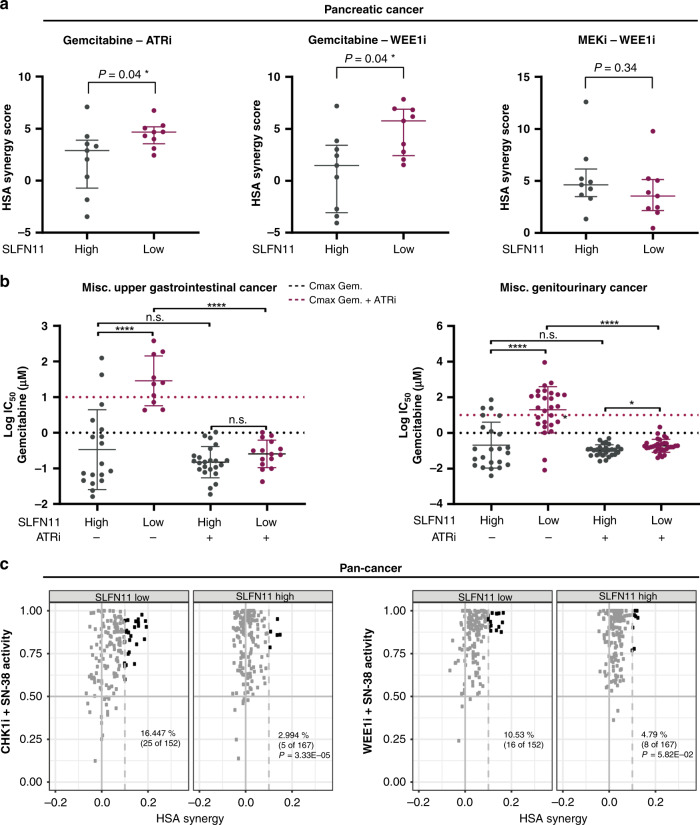
Resistance to broad DDA in SLFN11 absent/low cancers (different cancer types) can be overcome by inhibition of ATR, WEE1 or CHK1. **a** HSA synergy scores of the indicated combinations in SLFN11 high and low pancreatic cancer cell lines. Data are presented as medians with interquartile range (Wilcoxon test). *P < 0.05. **b** Log IC_50_ values of cell lines from the indicated cancers following treatments with gemcitabine (GDSC003 dataset) or gemcitabine/ATRi (additional screen, caveat). Data are presented as means ± s.d. *P < 0.05; ****P < 0.0001; ns not significant (one-way ANOVA with Dunnett’s T3 multiple comparisons test). The dashed lines indicate the maximum concentrations of gemcitabine or gemcitabine/ATRi used in the screen. Miscellaneous upper gastrointestinal cancer: HNSC head and neck squamous cell carcinoma, ESCA oesophageal carcinoma. Miscellaneous genitourinary cancer: PRAD prostate adenocarcinoma, BLCA bladder urothelial carcinoma, UCEC uterine corpus endometrial carcinoma, OV ovarian serous cystadenocarcinoma. **c** Scatter plots of SLFN11 low and high pan-cancer cell lines following 72-h combination treatments. The solid horizontal line indicates the threshold for the maximum activity of the combination and the dashed line the excess effect over the highest single agent (HSA). The black colour indicates cell lines that pass both thresholds and benefit from the combination treatment (indicated as percentages of total cell lines evaluated in the plots). *P* values derive from a two-sided Fisher’s exact test as further described in the “Methods”. SRA737 is the CHK1i in the left plot.

Finally, we verified whether the observations obtained in the DU145 isogenic pair were applicable more broadly. We first looked in pancreatic cancer where gemcitabine is used as a standard of care.^32^ Similarly, as in the DU145 isogenic cell line pair, synergy scores were significantly higher in SLFN11 low, than high, pancreatic cancer cell lines for combinations of gemcitabine with ATRi (*P* = 0.04, Wilcoxon test) or WEE1i (*P* = 0.04, Wilcoxon test) (Fig. 6a). By contrast, combinations with other DDRi (ATMi, olaparib) or other combination strategies (e.g., DDRi (ATRi/WEE1i) + non-DDAi (MEKi used as a representative example)) had the same effect in SLFN11 high and low pancreatic cancer cell lines (Fig. 6a and Supplementary Fig. S8A). In panels of miscellaneous upper gastrointestinal- and genitourinary cancers, SLFN11 low cell lines were significantly less responsive to gemcitabine monotherapy, but they could be re-sensitised by cotreatment with ATRi (Fig. 6b). Likewise, SLFN11 low pan-cancer cell lines globally benefitted from the combination of SN-38 with different inhibitors of the CHK kinase (SRA737, which targets preferentially CHK1, or prexasertib (a double CHK1/2i) (left panels of Fig. 6c and Supplementary Fig. S8B, respectively) or WEE1i (Fig. 6c, right), but not by combination with olaparib (Supplementary Fig. S8B, right). In summary, in all the analysed cancer models, SLFN11 status was linked to outcomes from DDA treatments and the resistant SLFN11 low population could be re-sensitised to DDA by combinations with ATR, WEE1 or CHK inhibitors.

## Discussion

In this study, we aimed to gain insights into the role of SLFN11 as a biomarker in cancer. In a multidisciplinary effort, combining different analyses in multiple cancer models and types, we demonstrate that SLFN11 represents an important biomarker for the stratification of DDA therapies, but not to DDRi monotherapies in breast cancer. Instead, we found that ATR, and for the first time WEE1i and CHKi could reverse resistance to broad DDA by targeting the replication stress response, inducing further DNA damage and ultimately leading to cell death in SLFN11-absent/low settings.

In work by others, SLFN11 staining was found to be present in the whole cell or preferentially localised in the cytosol of cancer cells.^5,8,11,22^ Here, we developed, validated and applied an in-house IHC assay for analysis of PDX models and demonstrate that, consistently with what was reported in cell lines, SLFN11 shows an almost exclusively nuclear localisation across a multitude of different cancer types tested.

In this study, SLFN11 protein levels were evaluated in different tumour models and compared to transcript levels in cell lines of the same tissue of origin (Fig. 1b, c). Despite the largely bimodal expression reported in cell lines, we only observed a bimodal protein pattern in some PDX models. Moreover, SLFN11 absent/low tumours were present across different cancer types tested (Fig. 1b), hinting that a significant percentage of tumours might be less responsive to DDA treatment due to absent/low SLFN11. In addition, we noted some differences between the overall expression in cell lines and protein levels in tissue. This discrepancy could be explained by in vitro to in vivo differences (e.g., cells from tumour tissues that grow in vitro might not completely recapitulate settings of the in vivo PDX models). Regardless, SLFN11 transcript and protein levels strongly correlated in the same breast cancer cohort (Fig. 2b), demonstrating that transcript and protein assessment equally reflects SLFN11 levels in xenograft tissues. Breast cancer cohorts provided some other interesting findings: they demonstrated elevated SLFN11 in cancers with worse prognosis. Recently it has been reported that the expression range of SLFN11 is greater in tumours than in normal tissue.^1,11^ Our data indicate that cancer cells might upregulate SLFN11 when they become more malignant.

Our in vitro studies indicate that SLFN11 is not modulated following chemotherapy treatment (Fig. 4a), which is different to what others have reported.^5,9^ Clearly, further investigations are required to shed lights into the modulation pattern of SLFN11 expression. Particularly relevant will be the analysis in paired biopsies in cohorts of patients across the development/evolution of the cancer, and with the progression of the treatment. It will be also important to determine the clinical relevance of SLFN11 levels in primary- versus acquired-resistance settings.

Our research shows that SLFN11 strongly correlates with the response to different DDA while the correlation was significantly lower for some DDRi and absent with non-DNA-damaging anticancer drugs. In particular, the significance of the correlation was highest for those DDA that selectively induce DNA damage in the S-phase and induce replication stress, such as gemcitabine, hydroxyurea and topoisomerase I inhibitors. For other agents that damage DNA during S-phase as well as during other phases of the cell cycle, such as etoposide or platinum, the differential response although still significant, was less pronounced. This observation confirms the hypothesis that SLFN11 has a key role in replication stress and accumulation of damage during the S-phase.^7,42^ ATRi, WEE1i and CHK1i have also been described to modulate the replication stress response leading to increased genomic instability in specific sub-population of cancer cells.^13,14,43^ However, only modest resistance to monotherapy treatments with ATRi or CHK1i were observed in the absence of SLFN11, possibly because these agents did not lead to significant accumulation of DNA damage in the conditions we tested. At clinically relevant concentrations,^39,40^ no significant correlation was found with treatments with WEE1i either. Future work is needed to investigate the mechanistic relationship between SLFN11 presence/absence and the response to ATRi, WEE1i and CHK1i monotherapies in clinical samples. We were also surprised by the limited impact of SLFN11 in our in vitro and in vivo models treated with the PARP inhibitor olaparib, particularly in light of other reports of a correlation of SLFN11 with PARPi.^3,4,6,22^ There could be several reasons for this. Firstly, we have used olaparib, while most of the published preclinical observations have been generated using talazoparib, an extreme PARP “trapper”, where even low concentrations are known to cause DNA damage.^44,45^ Second, the correlation between response to PARPi and SLFN11 have been to date mostly reported in lung, a tissue harbouring low levels BRCA1/2 mutations and homologous recombination deficiency,^5^ biomarkers of sensitivity to PARPi.^15^ In contrast, in this study we focused on breast cancers and the lack of association of SLFN11 to therapeutic response with PARPi in this indication may be due to the dominant role that BRCA plays in this setting as reported by others.^46^ Thus, we hypothesise that the biology of different cancer types might impact the relationship in different ways, which merits further investigations.

By focusing on DDA, we provide novel sensitisation strategies for SLFN11 low cancers, with evidence suggesting DDA combinations with WEE1i, ATRi or CHKi can overcome resistance arising from low or absent SLFN11. We show this at clinically relevant concentrations and doses of ATRi, WEE1i and CHK1i where their single-agent activity shows no differential effects in SLFN11-proficient versus SLFN11-deficient cancers, as well as at clinically relevant concentrations of DDA.^38–41,47^ We also demonstrate, the combinations restore/induce DNA damage and replication stress and ultimately induce cell death in SLFN11-deficient cells. Furthermore, we show that inhibitors of the key mediators of replication stress response pathways (ATRi, WEE1i, CHKi),^14,15^ but not inhibitors of factors involved in other DDR pathways (ATMi, DNA-PKi, PARPi), can reverse the SLFN11-mediated resistance (Fig. 3 and Supplementary Fig. S4). In summary, we propose the following model (Supplementary Fig. S8C): DDA treatment inflicts DNA damage and/or replication stress in cells. SLFN11-deficient cancer cells heavily rely on the S-G2/M checkpoints following DDA treatment which is sufficient to cope with DNA damage and replication stress to ultimately survive the chemotherapy. By contrast, when the checkpoint is abrogated by inhibition of ATR, CHK or WEE1, SLFN11-deficient cells progress faster through S-phase, accumulate DNA damage and replication stress and consequently undergo cell death. Of note, similar sensitisation strategies have been recently described for the combination of ATRi with camptothecin, trabectedin and irinotecan in some preclinical models.^7,18,19^ To our knowledge, however, this is the first time that sensitisation has been shown by combination of different DDAs with either CHK1i or WEE1i, and in a variety of cancer models. We believe that SLFN11 may become an essential predictive biomarker to DDA-based treatment regimens and that the combination of DDA with either WEE1i, CHKi or ATRi may be effective in treating cancers in which SLFN11 is absent or low and DDA are used as standard of care. For example, SLFN11 may be a predictive biomarker to gemcitabine-based treatment regiments: the combination of gemcitabine with either WEE1i or ATRi may be an effective combination strategy in ovarian or pancreatic cancer where most tumours show a low or absent expression of SLFN11 (Fig. 1b). Interestingly, in a randomised, phase II clinical trial, the combination of gemcitabine with the ATRi berzosertib was more efficacious than gemcitabine monotherapy treatment, in high-grade serous ovarian cancer (HGSOC) patients.^48^ Similarly, the combination of gemcitabine (and radiation) with WEE1i in pancreatic cancer showed efficacy in a recent phase I clinical trial.^49^ Future analysis of clinical cohorts will enable to ascertain whether the efficacy of the combinations may be attributed to low or absent SLFN11 protein in the tumour cells of these patients.

## Supplementary information

All supplementary information

## Data Availability

All the data supporting the findings of this study are available within the article and its Supplementary Information files and from the corresponding authors upon reasonable request. Researchers may obtain AZD1775, AZ7648, AZD6738 and AZD0156 with a material transfer agreement from AstraZeneca.
