# Supplementary material for: SLFN11 informs on standard of care and novel treatments in a wide range of cancer models
Source: Br J Cancer. 2020 Dec 18;124(5):951–62. doi: 10.1038/s41416-020-01199-4 (PMC7921667; doi:10.1038/s41416-020-01199-4)
Supplement: Supplementary file 1 — All supplementary information [file 41416_2020_1199_MOESM1_ESM.pdf]

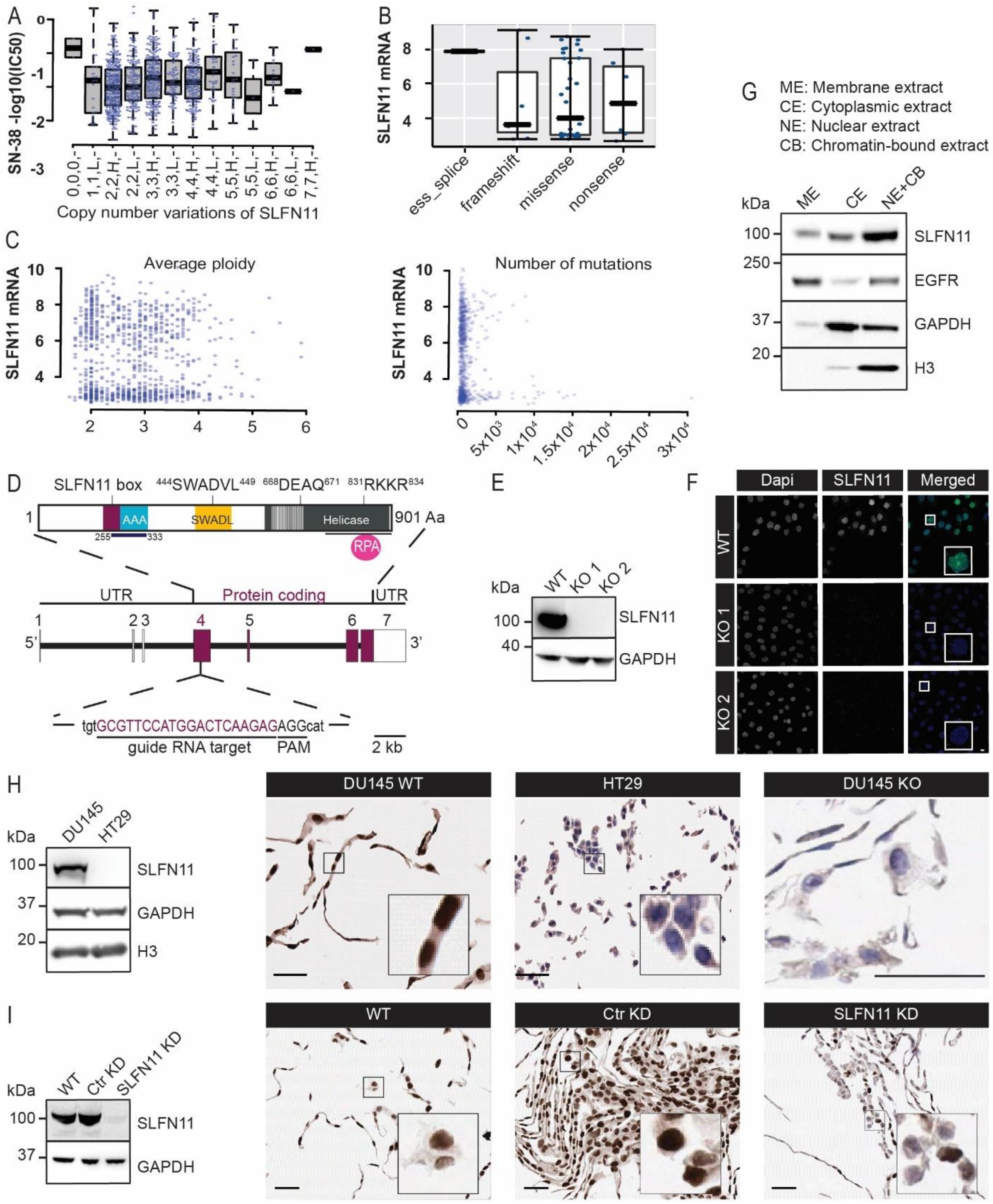

Fig. S1. SLFN11 is not associated with ploidy or mutational burden in cancer. The used SLFN11 IHC is accurate and specific.

(**A**) Copy number variations of SLFN11 in GDSC and correlation with response to the DDA TOP1 inhibitor SN-38. (**B**) Cell lines with SLFN11 mutations and SLFN11 RMA normalised gene expression in GDSC. ESS, splicing mutation. (**C**) Correlation of SLFN11 RMA normalised gene expression with ploidy and mutational burden in GDSC. (**D**) Domain structure of SLFN11 and scheme of the CRISPR CAS9-mediated SLFN11 KO in DU145 cells. UTR,

untranslated region; PAM, protospacer adjacent motif. The used IHC antibodies are directed against the indicated region in SLFN11 (blue solid line). (**E, F**) Validation of the KO of SLFN11 in DU145 cells by WB (**E**) and IF (scale bar, 10 µm) (**F**). (**G**) Subcellular fractionation of DU145 cells. Shown are immunoblots for the indicated proteins. EGFR served as loading control for the membrane fraction. **(H, I)** Left, Immunoblot for SLFN11 in the indicated whole total lysates. Right, IHC for SLFN11 in the indicated cell lines. The insets show a 4x magnification. Scale bars, 50 µM.

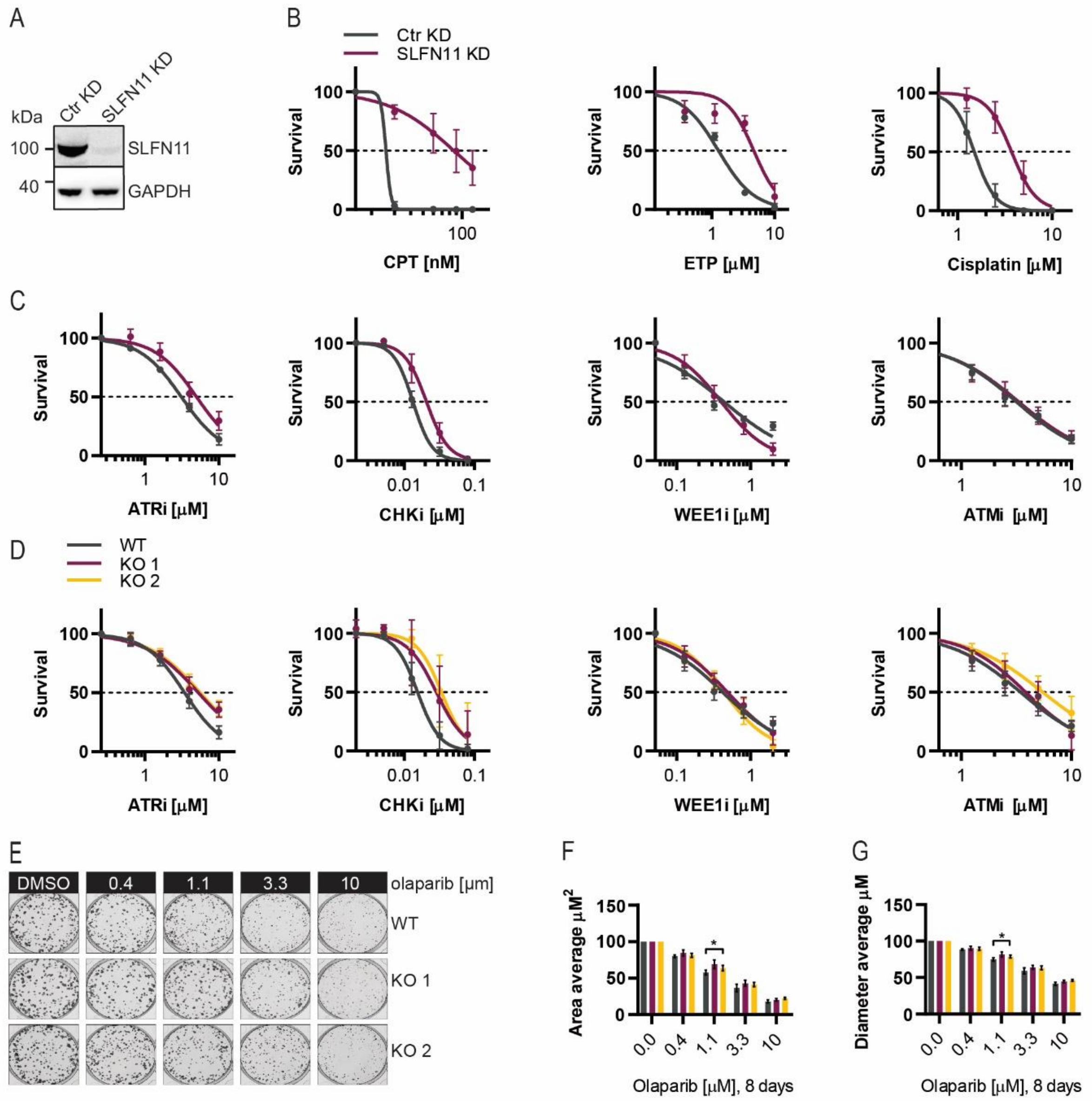

Fig. S2. SLFN11-deficient DU145 cells are resistant to DDA, but less to DDRi.

(**A**) Immunoblots for SLFN11 in representative lysates. (**B-C**) Response to camptothecin (CPT), etoposide (ETP) and cisplatin and the indicated DDRi in DU145 control knockdown (Ctr KD) and SLFN11 KD cells and (**D**) response to DDRi in isogenic DU145 cells as determined by CellTiter-Glo® luminescent cell viability assays. Data are presented as mean percentages ± s.d. (n=3-4) of the DMSO treated condition. (**E**) Representative pictures of the colony formation assay of the indicated DU145 cell lines following continuous treatment with olaparib for 8 days. (**F, G**) Quantification of area average colony size (**F**) and diameter average colony size (**G**) from experiments as shown in panel E. The bars represent mean percentages ± s.d. (n=3) of the DMSO treated condition. *P<0.05 (paired Student's t-test).

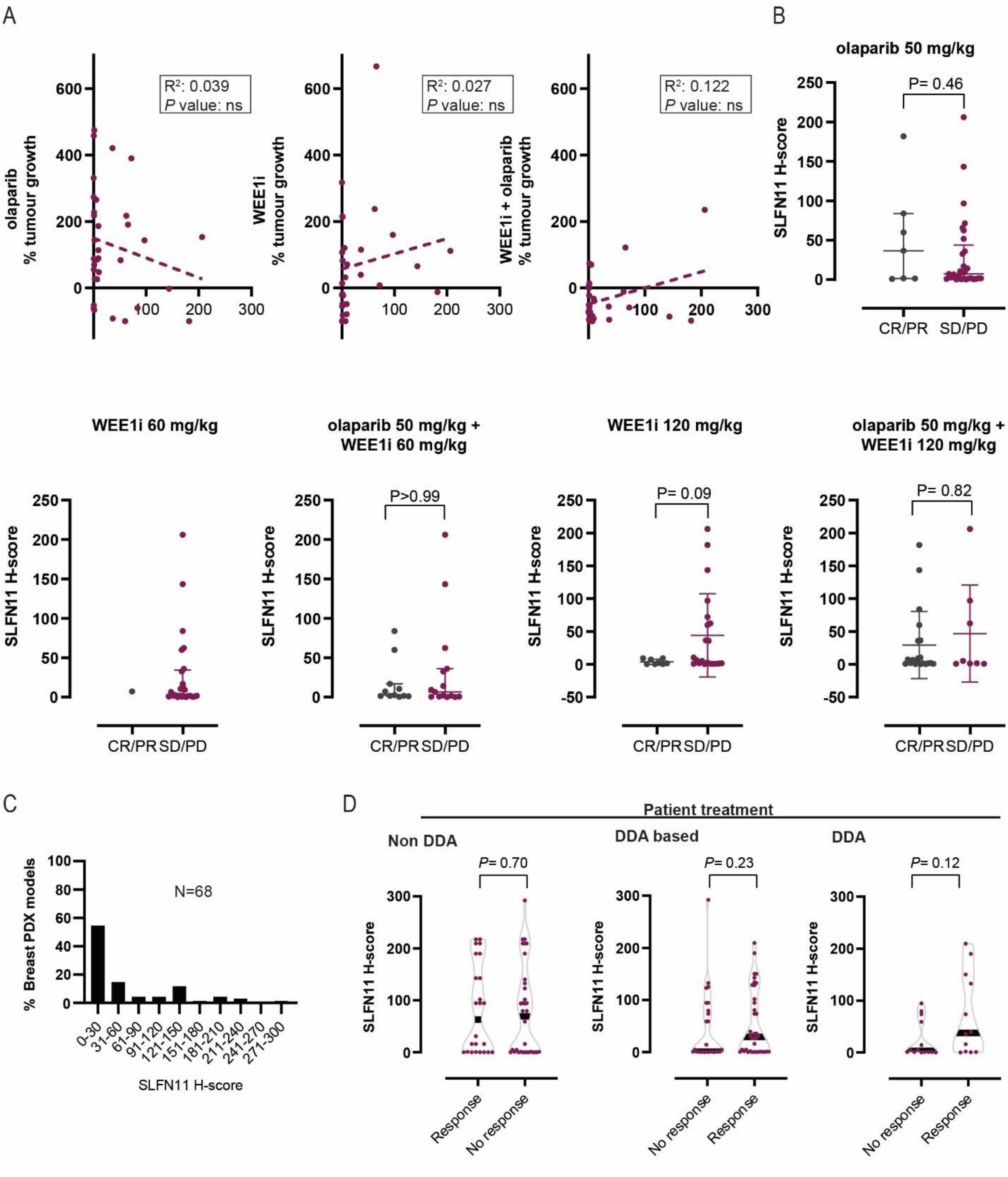

Fig. S3. SLFN11 is not associated with response to DDRi olaparib and WEE1i, but with DDA-based treatment in breast cancer patients.

(**A**) Correlation of SLFN11 (H-score) with % tumour growth in response to olaparib, WEE1i and the combination of olaparib and WEE1i treatment in 27 TNBC and 2 ovarian PDX models. The linear regression is shown by a dotted line. ns, not significant. (**B**) SLFN11 PDX models specified in A categorised as responder (CR/PR) and non-responder (SD/PD) to the indicated treatments. Data are presented as medians with interquartile range (Wilcoxon test). CR,

complete response; PR, partial response; SD, stable disease; PD, progressive disease. **(C)** Frequency distribution of SLFN11 H-scores in a second breast cancer cohort from Champions Oncology. **(D)** SLFN11 H-score measured in grafts from breast cancer patients that showed no response or response to non-DDA monotherapy or non-DDA combinations (left), DDA-based (combinations of DDA with DDA/non DDA, middle) and DDA monotherapy or DDA combination treatment, right. The violin plots represent medians (Wilcoxon test).

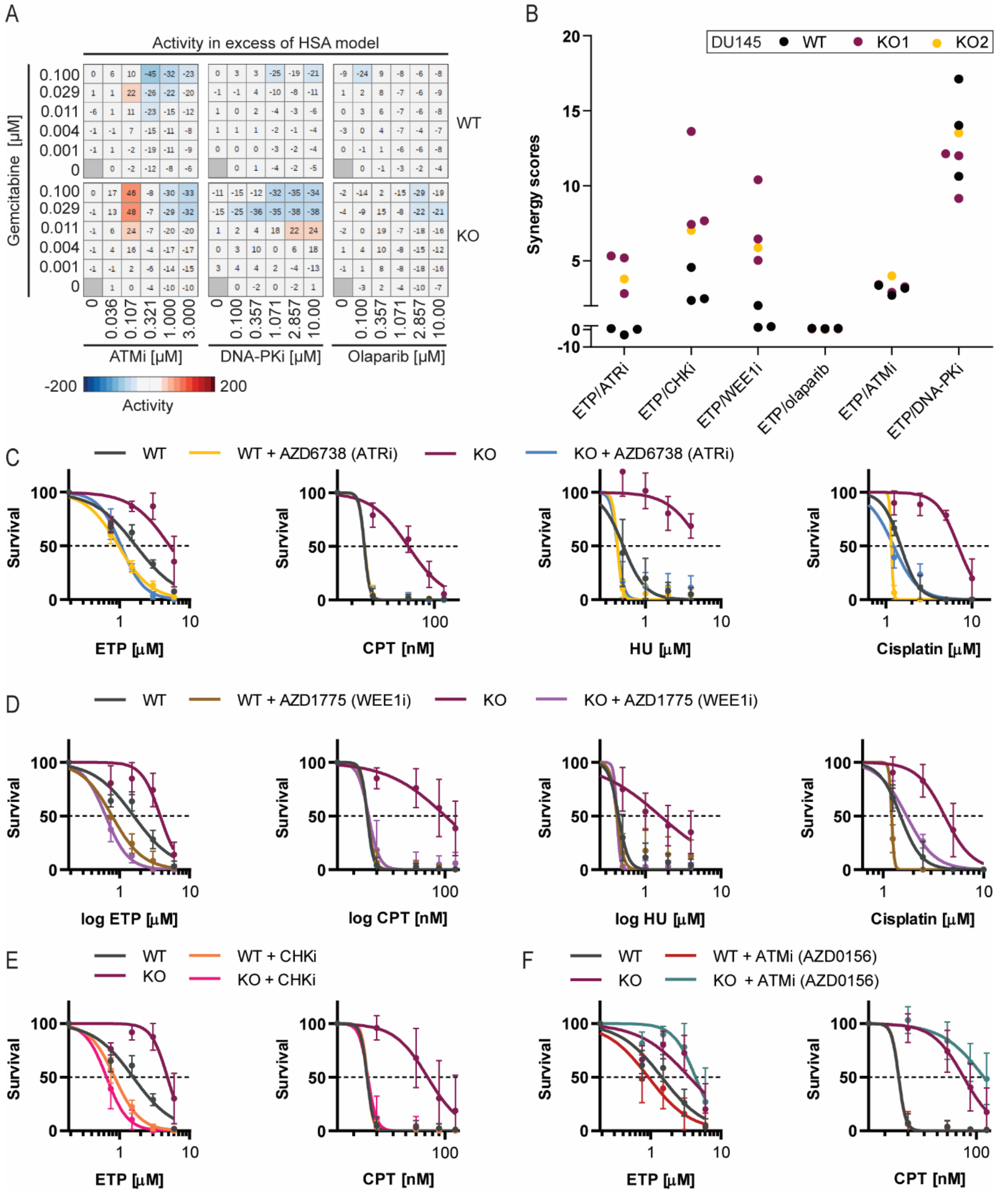

Fig. S4. Resistance to broad DDA in SLFN11-negative setting can be reversed by ATR, WEE1 and CHK inhibition, but not by ATMi, DNA-PKi or olaparib combinations.

(**A**) Representative heatmaps of the activity in excess of the HSA model shown for the indicated gemcitabine/DDRi combinations for the same conditions as in panel 3A. **(B)** HSA synergy scores of the tested etoposide-DDRi combinations in DU145 isogenic cells with the indicated drugs (in triplicates for WT and KO1; n=1 for KO2). (**C-F**) Response to different DDA in the absence or presence of 0.5 µM ATRi (**C**), 0.36 µM WEE1i (**D**), 0.005 µM CHK1i (**E**) and 1

μM ATMi (**F**) in DU145 isogenic cells as determined by CellTiter-Glo® luminescent cell viability assays. Data are presented as mean percentages ± s.d. (n=3-5) of the DMSO and single agent DDRi-treated conditions.

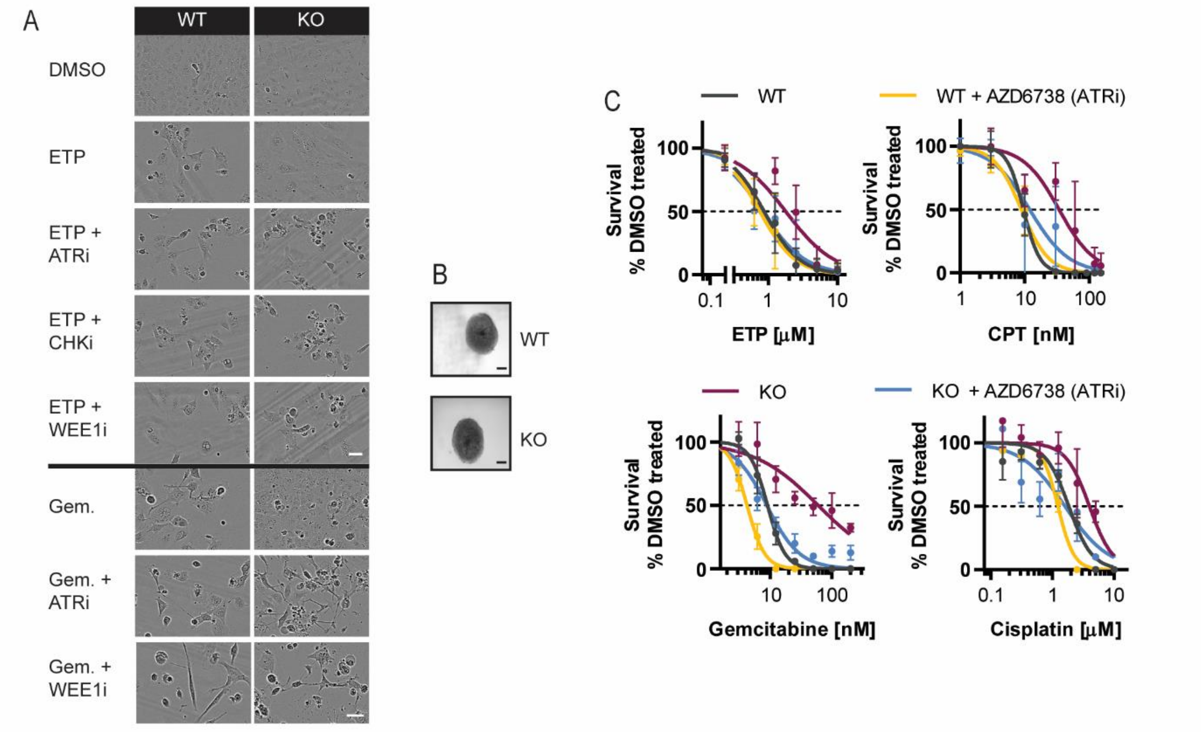

Fig. S5. Resistance to different DDA in SLFN11 low DU145 cells can be overcome by combinations with ATRi /WEE1i and CHK1i in 2D and 3D cell culture

(**A**) DIC pictures of DU145 isogenic cells following treatment with etoposide or gemcitabine in the absence or presence of the indicated DDRi (72 hour treatment). Scale bars, 10 µM. **(B)** Representative pictures of WT and SLFN11 KO spheroids in the absence of treatment. **(C)** Response to different DDA in the absence or presence of 0.5 µM ATRi in DU145 isogenic spheroids as determined by CellTiter-Glo® luminescent cell viability assays. Data are presented as mean percentages ± s.d. of the DMSO treated condition (n=4-5 for CPT and ETP treatment; n=1 for gemcitabine and cisplatin treatment).

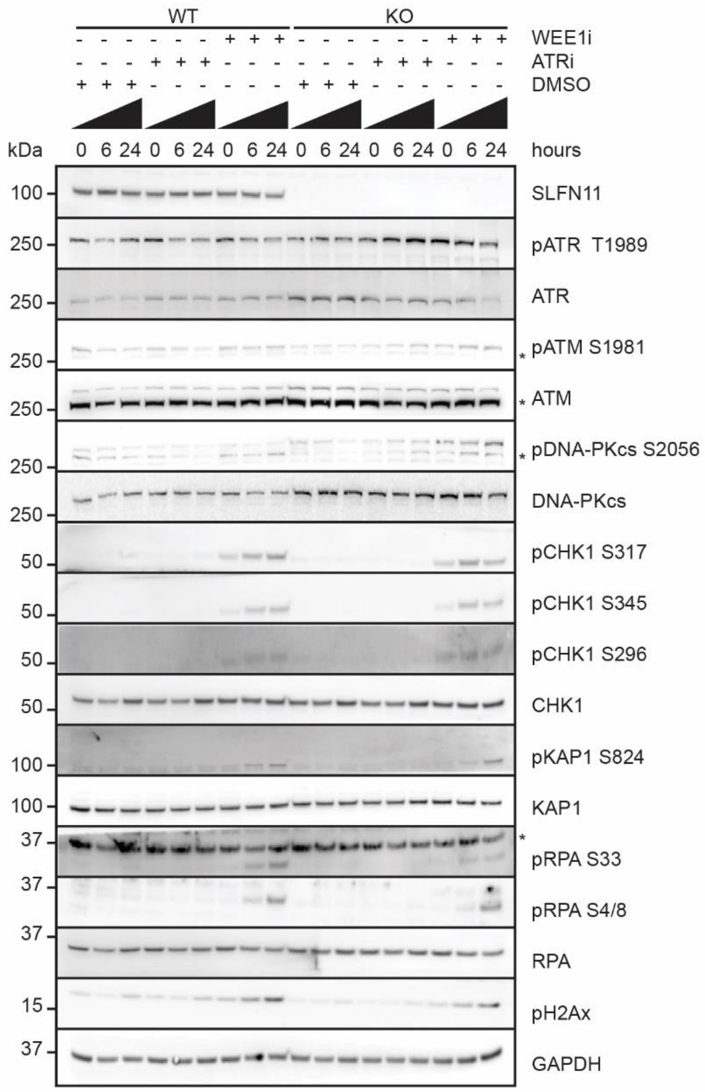

Fig. S6. WEE1i monotherapy induces some checkpoint activation, DNA damage and replication stress in cells.

Representative immunoblots of SLFN11 and the indicated biomarkers in DU145 isogenic pair treated for the indicated hours with DMSO, ATRi and WEE1i monotherapy. Similar results were obtained in a second, independent, experiment. *, aspecific bands.

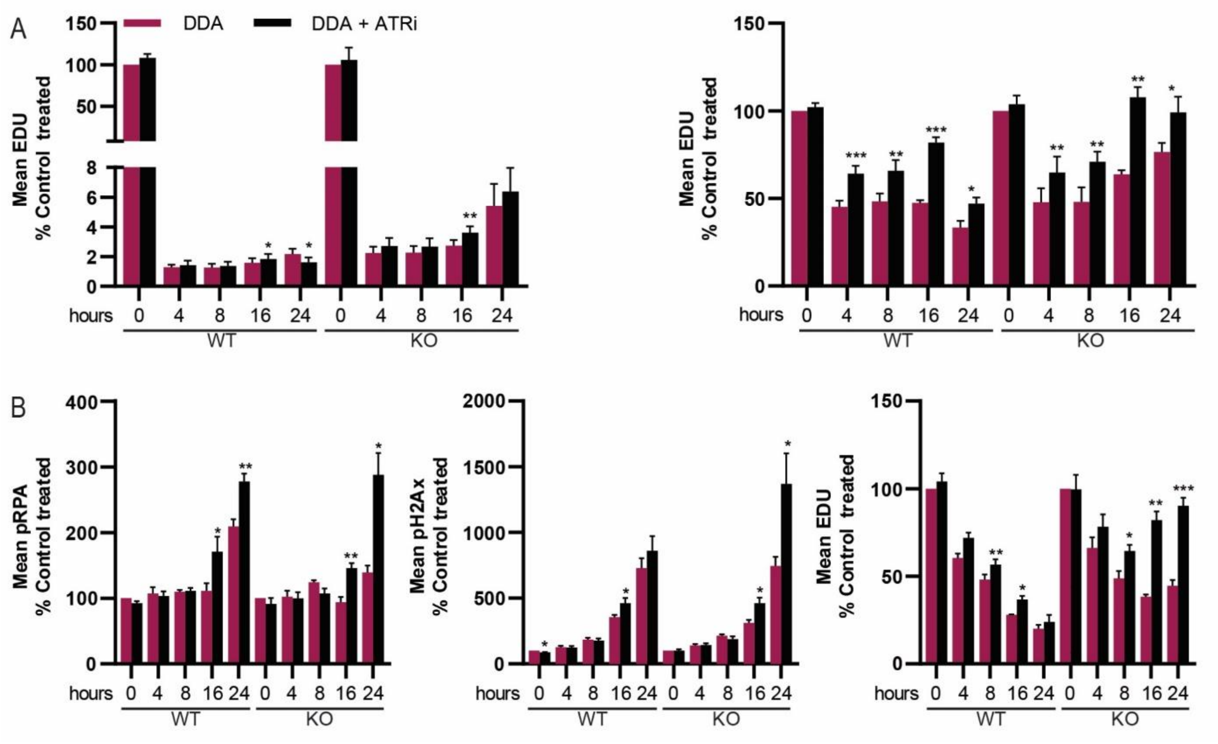

Fig. S7. ATR inhibition induces a S-phase checkpoint override, DNA damage and replication stress in SLFN11-deficient DDA-treated cells.

(A) EdU mean intensities in DU145 isogenic cells treated with gemcitabine, ATRi (left) or etoposide, ATRi (right) for the indicated time points. The data are presented as mean±s.e.m. (n=4) of the control treated condition. **P*<0.05; ***P*<0.01; ****P*<0.001 (paired Student's t-test). (**B**) pRPA (S4/8), pH2Ax and EdU mean intensities in DU145 isogenic cells treated with cisplatin, ATRi for the indicated time points. The data are presented as mean±s.e.m. (n=4) of the control treated condition. **P*<0.05; ***P*<0.01; ****P*<0.001 (paired Student's t-test).

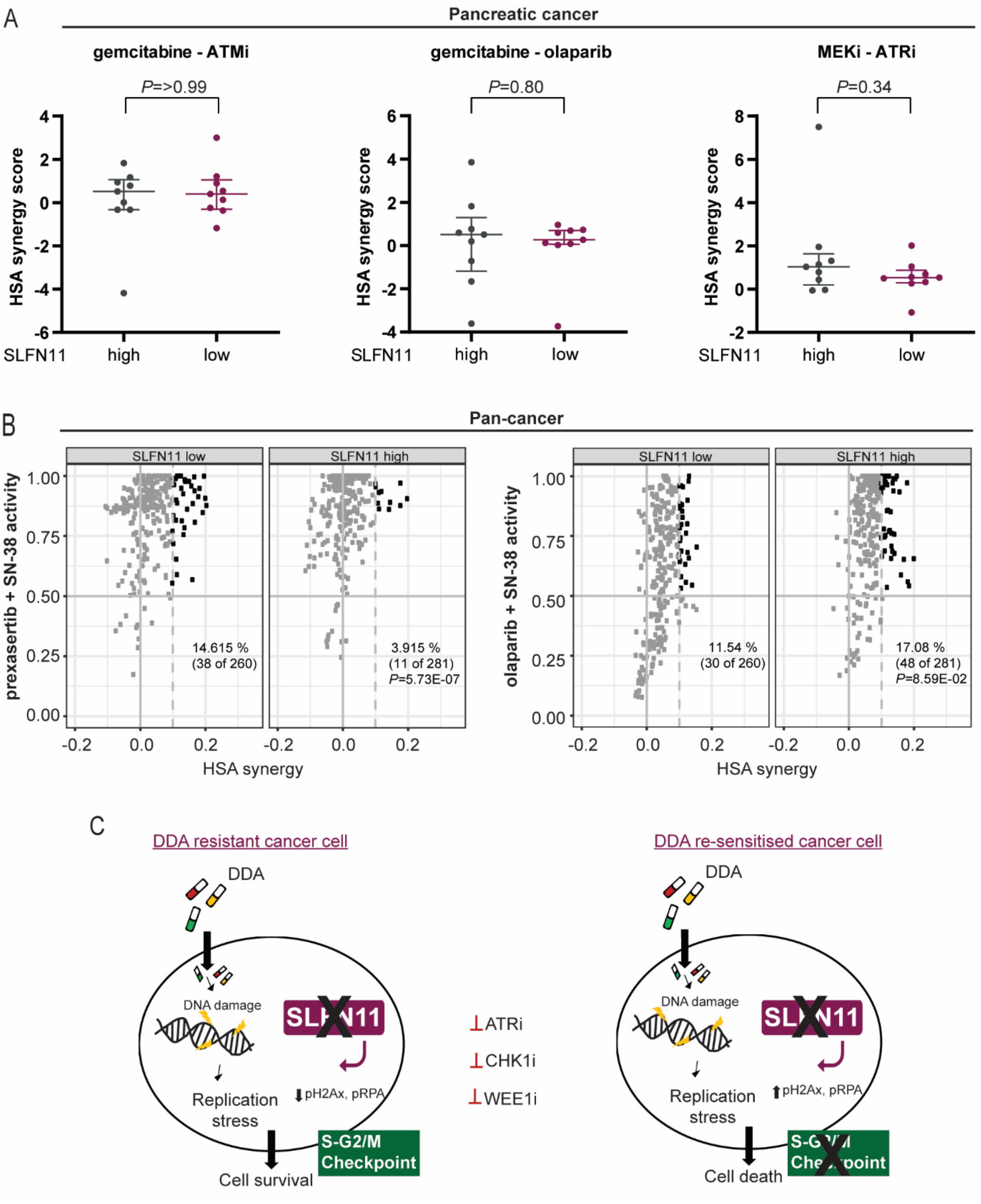

Fig. S8. Resistance to broad DDA in SLFN11 negative cancers (different cancer types) can be overcome by ATRi, WEE1i and CHKi, but not other DDRi, combinations and working model. **(A)** HSA synergy scores of the indicated combinations in SLFN11 high and low pancreatic cancer cell lines. Data are presented as medians with interquartile range (Wilcoxon test). **(B)** Scatter plots of SLFN11 low and high pan-cancer cell lines. The solid horizontal line indicates the threshold for the maximum activity of the combination and the dashed line the excess effect

over the highest single agent (HSA). The black color indicates cell lines that pass both thresholds and benefit from the combination treatment (indicated as percentages of total cell lines evaluated in the plots). *P*-values derive from a two-sided Fisher's exact test as further described in the material and methods. **(C)** Proposed working model. DDA treatment inflicts DNA damage and/or replication stress in cells. SLFN11-deficient cancer cells heavily rely on the S-G2/M checkpoints following DDA treatment which is sufficient to cope with DNA damage and replication stress to ultimately survive the chemotherapy. By contrast, when the checkpoint is abrogated by inhibition of ATRi, CHK1i or WEE1i, SLFN11-deficient cells progress faster through S-phase, accumulate DNA damage and replication stress and consequently undergo cell death.

Table S1: Summary table of all combination data.

	DU145 prostate cancer - HSA synergy scores						
	WT			KO1			KO2
Gem./ATRi	8.166	6.066	6.104	24.864	16.832	17.441	21.729
Gem./CHKi	9.928	6.194	7.433	36.844	16.210	21.486	21.673
Gem./WEE1i	9.685	8.324	10.545	25.066	10.816	16.454	15.710
Gem./olaparip	-1.003	-3.391	-2.065	-3.302	-6.676	-3.479	-5.894
Gem./ATMi	-3.129	-1.999	-4.574	1.214	-2.635	-2.298	2.492
Gem./DNA-PKi	-2.399	-2.925	-2.248	-3.879	-5.174	-2.172	-3.872
ETP/ATRi	0.081	0.593	-2.981	2.814	5.195	5.317	3.793
ETP/CHKi	2.360	4.559	2.477	7.674	13.617	7.420	7.022
ETP/WEE1i	2.027	1.709	1.251	5.028	6.463	10.402	5.866
ETP/olaparib	0.713	0.550	0.687	0.385	0.388	0.342	0.050
ETP/ATMi	3.174	3.373	2.710	2.902	3.273	3.410	4.002
ETP/DNA-PKi	14.020	10.634	17.121	9.158	12.124	12.001	13.516
LOG2 RPKM SLFN11	17.752			N/A			N/A

	Pancreatic cancer - HSA synergy scores						
	**AsPC-1**	**BxPC-3**	**Capan-1**	**Capan-2**	**CFPAC-1**	**HPAC**	**HPAF-II**
Gem./ATRi	4.309	3.310	4.254	5.309	-1.831	5.053	4.690
Gem./WEE1i	6.932	-3.413	3.832	7.852	-4.056	1.530	5.751
Gem./olaparip	0.691	3.856	0.512	0.724	-3.611	0.028	0.960
Gem./ATMi	2.999	0.520	-0.329	-0.361	-0.328	0.132	1.204
MEKi/WEE1i	2.459	4.624	12.588	9.781	3.655	0.469	3.888
MEKi/ATRi	-1.071	1.958	7.496	1.047	-0.052	0.692	2.012
LOG2 RPKM SLFN11	0.1285	2.4612	11.4846	0.0129	6.6666	0.1129	0.0439
	**Hs 766T**	**HuP-T4**	**KP-4**	**MIA PaCa-2**	**Panc 02.03**	**Panc 03.27**	**Panc 04.03**
Gem./ATRi	3.089	2.887	7.078	4.662	6.736	0.375	3.523
Gem./WEE1i	6.170	1.476	7.189	6.838	3.530	0.345	3.018
Gem./olaparip	0.264	-1.663	0.197	0.591	0.122	0.761	0.599
Gem./ATMi	0.897	0.010	1.821	0.398	0.548	0.782	0.949
MEKi/WEE1i	5.218	5.195	4.232	1.959	2.353	4.900	3.319
MEKi/ATRi	0.563	1.307	0.788	0.505	0.266	1.038	1.145
LOG2 RPKM SLFN11	0.0606	10.4555	11.4078	0.0364	0.0387	12.037	3.2811
	**Panc 08.13**	**Panc 10.05**	**PSN1**	**YAPC**			
Gem./ATRi	2.438	2.061	4.000	-3.461			
Gem./WEE1i	2.784	2.418	2.049	-2.724			
Gem./olaparip	0.089	1.822	-3.729	-0.710			
Gem./ATMi	-0.249	1.168	-1.178	-4.177			
MEKi/WEE1i	3.550	1.343	5.037	7.102			
MEKi/ATRi	0.540	0.439	0.334	-0.037			
LOG2 RPKM SLFN11	0.334	8.8112	1.8165	18.6292			

	Misc. upper gastrointestinal cancer						
	**Cell line**	**A253**	**BICR22**	**CAL27**	**CAL33**	**COLO680N**	**DETROIT562**
	log IC_{50} μM	-1.347	-1.180	-1.339	-1.425	1.626	-0.108
	TCGA	N/A	HNSC	HNSC	HNSC	ESCA	HNSC
	LOG2 RPKM SLFN11	6.807	2.278	10.235	10.828	12.396	3.539
	**Cell line**	**FADU**	**HSC2**	**HSC3**	**HSC4**	**KYSE140**	**KYSE180**
	log IC_{50} μM	-1.789	-1.621	0.348	-1.141	0.859	0.632
	TCGA	HNSC	HNSC	HNSC	HNSC	ESCA	ESCA
	LOG2 RPKM SLFN11	24.581	10.674	8.134	16.046	0.008	0.029
	**Cell line**	**KYSE270**	**KYSE410**	**KYSE450**	**KYSE520**	**KYSE70**	**OE21**
	log IC_{50} μM	-1.184	2.204	1.402	2.579	2.276	1.050
	TCGA	ESCA	ESCA	ESCA	ESCA	ESCA	ESCA
	LOG2 RPKM SLFN11	9.004	0.029	0.030	0.015	0.012	1.489
	**Cell line**	**OE33**	**SCC15**	**SCC4**	**SCC9**	**TE1**	**TE15**
	log IC_{50} μM	-0.297	-0.115	0.792	1.369	1.546	-1.077
	TCGA	ESCA	HNSC	HNSC	HNSC	ESCA	ESCA
	LOG2 RPKM SLFN11	3.866	5.084	2.551	0.054	0.031	12.801
	**Cell line**	**TE4**	**TE5**	**TE6**	**TE9**		
	log IC_{50} μM	2.099	-0.867	0.645	0.088		
	TCGA	ESCA	ESCA	ESCA	ESCA		
Gem.	LOG2 RPKM SLFN11	2.237	18.923	0.191	5.880		
	**Cell line**	**A253**	**BHY**	**BICR22**	**CAL27**	**CAL33**	**COLO680N**
	log IC_{50} μM	-0.906	-1.114	-1.449	-0.781	-0.791	0.017
	TCGA	N/A	HNSC	HNSC	HNSC	HNSC	ESCA
	LOG2 RPKM SLFN11	6.807	8.309	2.278	10.235	10.828	12.396
	**Cell line**	**DETROIT562**	**ECGI10**	**FADU**	**HSC2**	**HSC3**	**HSC4**
	log IC_{50} μM	-1.149	-0.883	-0.848	-0.956	-1.029	-1.027
	TCGA	HNSC	ESCA	HNSC	HNSC	HNSC	HNSC
	LOG2 RPKM SLFN11	3.539	0.026	24.581	10.674	8.134	16.046
	**Cell line**	**KYSE150**	**KYSE180**	**KYSE270**	**KYSE30**	**KYSE410**	**KYSE450**
	log IC_{50} μM	-0.497	-1.032	-1.558	-1.373	-0.458	-0.601
	TCGA	ESCA	ESCA	ESCA	ESCA	ESCA	ESCA
	LOG2 RPKM SLFN11	0.027	0.029	9.004	0.011	0.029	0.030
	**Cell line**	**KYSE510**	**KYSE520**	**KYSE70**	**OE19**	**OE21**	**OE33**
	log IC_{50} μM	-1.729	-0.121	-0.741	-0.733	-1.004	-0.777
	TCGA	ESCA	ESCA	ESCA	ESCA	ESCA	ESCA
	LOG2 RPKM SLFN11	15.703	0.015	0.012	0.036	1.489	3.866
	**Cell line**	**PECAPJ15**	**SCC15**	**SCC25**	**SCC4**	**SCC9**	**TE1**
	log IC_{50} μM	-0.968	-0.648	-0.142	-0.328	-0.082	0.008
	TCGA	HNSC	HNSC	HNSC	HNSC	HNSC	ESCA
	LOG2 RPKM SLFN11	9.517	5.084	6.175	2.551	0.054	0.031
	**Cell line**	**TE10**	**TE11**	**TE15**	**TE4**	**TE5**	**TE6**
	log IC_{50} μM	-0.696	-0.502	-0.083	-0.796	-0.822	-0.248
	TCGA	ESCA	ESCA	ESCA	ESCA	ESCA	ESCA
	LOG2 RPKM SLFN11	3.294	0.282	12.801	2.237	18.923	0.191
	**Cell line**	**TE8**	**TE9**				
	log IC_{50} μM	-0.621	-0.447				
	TCGA	ESCA	ESCA				
Gem./ATRi	LOG2 RPKM SLFN11	1.268	5.880				

	Misc. genitourinary cancer						
	**Cell line**	**22RV1**	**639V**	**647V**	**A2780**	**AN3CA**	**BFTC905**
	log IC_{50} μM	-0.898	-1.960	-1.474	-2.092	2.353	-2.403
	TCGA	PRAD	BLCA	BLCA	N/A	UCEC	BLCA
	LOG2 RPKM SLFN11	10.422	25.461	23.710	0.009	0.015	2.904
	**Cell line**	**CAL29**	**CAOV4**	**COLO684**	**DU145**	**EFO21**	**EN**
	log IC_{50} μM	-1.375	0.512	1.242	-0.829	0.341	2.053
	TCGA	BLCA	OV	UCEC	PRAD	OV	UCEC
	LOG2 RPKM SLFN11	5.555	0.774	0.068	17.752	0.495	0.024
	**Cell line**	**ES2**	**ESS1**	**FUOV1**	**HT1197**	**HT1376**	**IGROV1**

	log IC_{50} μM	-1.951	-1.821	3.956	2.124	2.371	0.086
	TCGA	N/A	N/A	OV	BLCA	BLCA	OV
	LOG2 RPKM SLFN11	10.888	26.160	0.076	0.022	0.743	0.028
	**Cell line**	**J82**	**KLE**	**KU1919**	**KURAMOCHI**	**NCAPCLONEFG**	**MFE280**
	log IC_{50} μM	-0.071	2.802	-1.120	2.660	0.010	1.949
	TCGA	BLCA	UCEC	BLCA	N/A	PRAD	UCEC
	LOG2 RPKM SLFN11	9.187	0.016	3.851	0.035	0.042	0.070
	**Cell line**	**MFE296**	**MFE319**	**OAW28**	**OC314**	**OV56**	**OV90**
	log IC_{50} μM	1.396	2.082	0.991	-1.786	0.087	0.317
	TCGA	UCEC	UCEC	OV	OV	OV	OV
	LOG2 RPKM SLFN11	16.162	0.008	3.814	42.933	15.365	3.165
	**Cell line**	**OVCAR4**	**OVCAR8**	**OVISE**	**OVTOKO**	**PC3**	**RKN**
	log IC_{50} μM	2.146	-1.665	1.487	1.859	1.382	2.236
	TCGA	OV	OV	OV	OV	PRAD	N/A
	LOG2 RPKM SLFN11	0.053	3.892	0.496	11.970	3.459	1.263
	**Cell line**	**RL952**	**RMGI**	**RT112**	**RT4**	**SKOV3**	**SW1710**
	log IC_{50} μM	0.981	1.926	0.602	-2.015	0.975	1.441
	TCGA	N/A	N/A	BLCA	BLCA	OV	BLCA
	LOG2 RPKM SLFN11	0.025	0.026	0.024	4.572	10.693	0.095
	**Cell line**	**SW780**	**TCCSUP**	**TOV112D**	**TOV21G**	**TYKNU**	**UMUC3**
	log IC_{50} μM	0.871	1.414	-1.631	0.627	-0.886	-0.918
	TCGA	BLCA	BLCA	OV	OV	N/A	BLCA
	LOG2 RPKM SLFN11	1.226	1.053	18.397	0.050	24.518	23.601
	**Cell line**	**VCAP**	**VMCUB1**				
	log IC_{50} μM	0.441	-1.525				
	TCGA	PRAD	BLCA				
Gem.	LOG2 RPKM SLFN11	0.025	0.339				
	**Cell line**	**22RV1**	**5637.000**	**639V**	**647V**	**A2780**	**AN3CA**
	log IC_{50} μM	-0.306	-0.897	-0.771	-1.257	-0.900	-0.850
	TCGA	PRAD	BLCA	BLCA	BLCA	N/A	UCEC
	LOG2 RPKM SLFN11	10.422	1.607	25.461	23.710	0.009	0.015
	**Cell line**	**BFTC905**	**CAL29**	**CAOV4**	**COLO684**	**DU145**	**EFO21**
	log IC_{50} μM	-1.593	-1.545	-0.944	-0.598	-1.224	-0.930
	TCGA	BLCA	BLCA	OV	UCEC	PRAD	OV
	LOG2 RPKM SLFN11	2.904	5.555	0.774	0.068	17.752	0.495
	**Cell line**	**EFO27**	**EN**	**ES2**	**ESS1**	**FUOV1**	**HT1197**
	log IC_{50} μM	-0.802	-0.033	-1.114	-0.956	-0.181	-0.387
	TCGA	N/A	UCEC	N/A	N/A	OV	BLCA
	LOG2 RPKM SLFN11	0.381	0.024	10.888	26.160	0.076	0.022
	**Cell line**	**HT1376**	**IGROV1**	**HIKAWAHERAKLIO02E**	**J82**	**JHOS2**	**JHOS4**
	log IC_{50} μM	-0.828	-0.798	-0.626	-0.827	-0.522	-0.566
	TCGA	BLCA	OV	UCEC	BLCA	OV	OV
	LOG2 RPKM SLFN11	0.743	0.028	0.019	9.187	10.861	2.560
	**Cell line**	**KLE**	**KU1919**	**KURAMOCHI**	**NCAPCLONEFG**	**MESSA**	**MFE280**
	log IC_{50} μM	-0.333	-1.352	-0.272	-0.572	-0.575	0.326
	TCGA	UCEC	BLCA	N/A	PRAD	N/A	UCEC
	LOG2 RPKM SLFN11	0.016	3.851	0.035	0.042	0.013	0.070
	**Cell line**	**MFE296**	**MFE319**	**OAW28**	**OAW42**	**OC314**	**OV56**
	log IC_{50} μM	-0.951	-0.709	-1.246	-1.100	-0.969	-0.946
	TCGA	UCEC	UCEC	OV	OV	OV	OV
	LOG2 RPKM SLFN11	16.162	0.008	3.814	22.315	42.933	15.365
	**Cell line**	**OV7**	**OV90**	**OVCAR4**	**OVCAR8**	**OVISE**	**OVK18**
	log IC_{50} μM	-0.912	-0.407	-0.742	-0.902	-0.617	-0.720
	TCGA	OV	OV	OV	OV	OV	OV
	LOG2 RPKM SLFN11	14.146	3.165	0.053	3.892	0.496	0.032
	**Cell line**	**OVTOKO**	**PC3**	**RKN**	**RL952**	**RMGI**	**RT112**
	log IC_{50} μM	-0.625	-0.931	-0.791	-0.737	-0.767	-1.344
	TCGA	OV	PRAD	N/A	N/A	N/A	BLCA
	LOG2 RPKM SLFN11	11.970	3.459	1.263	0.025	0.026	0.024
	**Cell line**	**RT4**	**SCABER**	**SKOV3**	**SNGM**	**SW1710**	**SW780**
	log IC_{50} μM	-0.980	-0.969	-1.122	-1.266	-0.795	-1.229
	TCGA	BLCA	BLCA	OV	UCEC	BLCA	BLCA
	LOG2 RPKM SLFN11	4.572	0.350	10.693	0.037	0.095	1.226
	**Cell line**	**T24**	**TCCSUP**	**TOV112D**	**TOV21G**	**TYKNU**	**UMUC3**
	log IC_{50} μM	-0.926	-1.396	-0.847	-1.050	-1.236	-1.267
	TCGA	BLCA	BLCA	OV	OV	N/A	BLCA
	LOG2 RPKM SLFN11	15.078	1.053	18.397	0.050	24.518	23.601
	**Cell line**	**VCAP**					
	log IC_{50} μM	-0.675					
	TCGA	PRAD					
Gem./ATRi	LOG2 RPKM SLFN11	0.025					

**Pan cancer**							
**COSMIC_ID**	**CELL_LINE_NAME**	**TISSUE**	**CANCER_TYPE**	**combination**	**MaxE**	**HSA**	**SLFN11 log2(TPM+1)**
684052.000	A673	Bone	Ewing's Sarcoma	SRA737 + SN-38	0.945	0.001	6.406
684052.000	A673	Bone	Ewing's Sarcoma	AZD1775 + SN-38	0.973	0.044	6.406
684052.000	A673	Bone	Ewing's Sarcoma	Prexasertib + SN-38	1.000	0.040	6.406
684052.000	A673	Bone	Ewing's Sarcoma	SN-38 + Olaparib	1.000	0.117	6.406
684072.000	SK-ES-1	Bone	Ewing's Sarcoma	Prexasertib + SN-38	1.000	0.012	6.497
684072.000	SK-ES-1	Bone	Ewing's Sarcoma	SN-38 + Olaparib	1.000	0.084	6.497
687448.000	COLO-829	Skin	Melanoma	Prexasertib + SN-38	0.573	0.018	3.686
687448.000	COLO-829	Skin	Melanoma	SN-38 + Olaparib	0.663	0.003	3.686
687455.000	RT4	Bladder	Bladder Carcinoma	SN-38 + Olaparib	0.831	0.082	3.529
687455.000	RT4	Bladder	Bladder Carcinoma	Prexasertib + SN-38	0.831	0.005	3.529
687459.000	TCCSUP	Bladder	Bladder Carcinoma	Prexasertib + SN-38	0.890	0.147	2.160
687459.000	TCCSUP	Bladder	Bladder Carcinoma	SN-38 + Olaparib	0.665	0.104	2.160
687505.000	C-33-A	Cervix	Cervical Carcinoma	SRA737 + SN-38	0.943	0.073	3.828
687505.000	C-33-A	Cervix	Cervical Carcinoma	SN-38 + Olaparib	0.747	0.069	3.828
687505.000	C-33-A	Cervix	Cervical Carcinoma	Prexasertib + SN-38	1.000	0.081	3.828
687505.000	C-33-A	Cervix	Cervical Carcinoma	AZD1775 + SN-38	1.000	0.122	3.828
687506.000	C-4-I	Cervix	Cervical Carcinoma	SN-38 + Olaparib	0.928	0.069	3.370
687506.000	C-4-I	Cervix	Cervical Carcinoma	Prexasertib + SN-38	0.998	0.019	3.370
687561.000	42-MG-BA	Central Nervous System	Glioblastoma	SN-38 + Olaparib	1.000	0.095	5.689
687561.000	42-MG-BA	Central Nervous System	Glioblastoma	Prexasertib + SN-38	1.000	0.052	5.689
687562.000	8-MG-BA	Central Nervous System	Glioblastoma	Prexasertib + SN-38	0.982	0.004	6.453
687562.000	8-MG-BA	Central Nervous System	Glioblastoma	SN-38 + Olaparib	0.986	0.078	6.453
687563.000	A172	Central Nervous System	Glioblastoma	Prexasertib + SN-38	0.798	0.011	5.063
687563.000	A172	Central Nervous System	Glioblastoma	SN-38 + Olaparib	0.842	0.087	5.063
687568.000	GB-1	Central Nervous System	Glioblastoma	Prexasertib + SN-38	0.741	-0.018	5.613
687568.000	GB-1	Central Nervous System	Glioblastoma	SN-38 + Olaparib	0.781	0.075	5.613
687586.000	T98G	Central Nervous System	Glioblastoma	SN-38 + Olaparib	0.632	0.121	0.070
687586.000	T98G	Central Nervous System	Glioblastoma	Prexasertib + SN-38	0.899	0.031	0.070

687586.000	T98G	Central Nervous System	Glioblastoma	SRA737 + SN-38	0.922	0.069	0.070
687586.000	T98G	Central Nervous System	Glioblastoma	AZD1775 + SN-38	0.957	0.071	0.070
687777.000	Calu-3	Lung	Lung Adenocarcinoma	Prexasertib + SN-38	0.582	0.007	4.355
687777.000	Calu-3	Lung	Lung Adenocarcinoma	SRA737 + SN-38	0.674	-0.019	4.355
687777.000	Calu-3	Lung	Lung Adenocarcinoma	AZD1775 + SN-38	0.804	0.057	4.355
687777.000	Calu-3	Lung	Lung Adenocarcinoma	SN-38 + Olaparib	0.398	0.100	4.355
687780.000	COR-L23	Lung	Non-Small Cell Lung Carcinoma	SN-38 + Olaparib	0.675	0.048	1.036
687780.000	COR-L23	Lung	Non-Small Cell Lung Carcinoma	Prexasertib + SN-38	0.811	0.086	1.036
687787.000	LK-2	Lung	Squamous Cell Lung Carcinoma	SRA737 + SN-38	0.906	0.195	1.029
687787.000	LK-2	Lung	Squamous Cell Lung Carcinoma	AZD1775 + SN-38	0.987	0.119	1.029
687787.000	LK-2	Lung	Squamous Cell Lung Carcinoma	Prexasertib + SN-38	0.925	0.134	1.029
687787.000	LK-2	Lung	Squamous Cell Lung Carcinoma	SN-38 + Olaparib	0.456	0.146	1.029
687799.000	NCI-H1648	Lung	Lung Adenocarcinoma	AZD1775 + SN-38	0.861	0.051	1.157
687799.000	NCI-H1648	Lung	Lung Adenocarcinoma	Prexasertib + SN-38	0.949	0.040	1.157
687799.000	NCI-H1648	Lung	Lung Adenocarcinoma	SN-38 + Olaparib	0.915	0.025	1.157
687799.000	NCI-H1648	Lung	Lung Adenocarcinoma	SRA737 + SN-38	0.849	0.044	1.157
687800.000	NCI-H1650	Lung	Lung Adenocarcinoma	Prexasertib + SN-38	0.714	-0.004	0.757
687800.000	NCI-H1650	Lung	Lung Adenocarcinoma	SN-38 + Olaparib	0.575	0.036	0.757
687812.000	NCI-H2085	Lung	Non-Small Cell Lung Carcinoma	Prexasertib + SN-38	0.906	-0.002	0.084
687812.000	NCI-H2085	Lung	Non-Small Cell Lung Carcinoma	SN-38 + Olaparib	0.132	-0.012	0.084
687812.000	NCI-H2085	Lung	Non-Small Cell Lung Carcinoma	AZD1775 + SN-38	0.776	0.014	0.084
687812.000	NCI-H2085	Lung	Non-Small Cell Lung Carcinoma	SRA737 + SN-38	0.665	0.021	0.084
687815.000	NCI-H2170	Lung	Squamous Cell Lung Carcinoma	SN-38 + Olaparib	0.969	0.093	3.995
687815.000	NCI-H2170	Lung	Squamous Cell Lung Carcinoma	Prexasertib + SN-38	0.969	0.011	3.995
687819.000	NCI-H2342	Lung	Lung Adenocarcinoma	Prexasertib + SN-38	0.338	-0.042	4.066
687819.000	NCI-H2342	Lung	Lung Adenocarcinoma	SRA737 + SN-38	0.740	0.098	4.066
687819.000	NCI-H2342	Lung	Lung Adenocarcinoma	SN-38 + Olaparib	0.236	0.031	4.066
687819.000	NCI-H2342	Lung	Lung Adenocarcinoma	AZD1775 + SN-38	0.943	0.067	4.066
687821.000	NCI-H2405	Lung	Lung Adenocarcinoma	SN-38 + Olaparib	0.472	0.029	5.098
687821.000	NCI-H2405	Lung	Lung Adenocarcinoma	Prexasertib + SN-38	0.579	-0.003	5.098
687829.000	NCI-H661	Lung	Non-Small Cell Lung Carcinoma	Prexasertib + SN-38	0.886	0.110	3.908
687829.000	NCI-H661	Lung	Non-Small Cell Lung Carcinoma	SN-38 + Olaparib	0.548	0.079	3.908
687983.000	DMS-114	Lung	Small Cell Lung Carcinoma	Prexasertib + SN-38	0.698	0.053	3.502
687983.000	DMS-114	Lung	Small Cell Lung Carcinoma	AZD1775 + SN-38	0.718	0.064	3.502
687983.000	DMS-114	Lung	Small Cell Lung Carcinoma	SN-38 + Olaparib	0.491	0.085	3.502
687983.000	DMS-114	Lung	Small Cell Lung Carcinoma	SRA737 + SN-38	0.608	0.012	3.502
687985.000	DMS-273	Lung	Small Cell Lung Carcinoma	Prexasertib + SN-38	0.994	0.051	4.135
687985.000	DMS-273	Lung	Small Cell Lung Carcinoma	SN-38 + Olaparib	0.799	0.032	4.135
687995.000	NCI-H1048	Lung	Small Cell Lung Carcinoma	Prexasertib + SN-38	1.000	0.059	5.156
687995.000	NCI-H1048	Lung	Small Cell Lung Carcinoma	SN-38 + Olaparib	1.000	0.120	5.156
687995.000	NCI-H1048	Lung	Small Cell Lung Carcinoma	SN-38 + Olaparib	0.974	0.116	5.156
687995.000	NCI-H1048	Lung	Small Cell Lung Carcinoma	Prexasertib + SN-38	1.000	0.073	5.156
687997.000	NCI-H1092	Lung	Small Cell Lung Carcinoma	Prexasertib + SN-38	0.854	0.035	5.743
687997.000	NCI-H1092	Lung	Small Cell Lung Carcinoma	SN-38 + Olaparib	0.540	0.007	5.743
688010.000	NCI-H1963	Lung	Small Cell Lung Carcinoma	Prexasertib + SN-38	1.000	0.030	0.163
688010.000	NCI-H1963	Lung	Small Cell Lung Carcinoma	SN-38 + Olaparib	0.854	0.111	0.163
688023.000	NCI-H446	Lung	Small Cell Lung Carcinoma	Prexasertib + SN-38	1.000	0.117	0.057
688023.000	NCI-H446	Lung	Small Cell Lung Carcinoma	SN-38 + Olaparib	0.923	0.080	0.057
688023.000	NCI-H446	Lung	Small Cell Lung Carcinoma	Prexasertib + SN-38	0.998	0.115	0.057
688023.000	NCI-H446	Lung	Small Cell Lung Carcinoma	SN-38 + Olaparib	0.886	0.057	0.057
688025.000	NCI-H526	Lung	Small Cell Lung Carcinoma	SN-38 + Olaparib	1.000	0.043	5.584
688025.000	NCI-H526	Lung	Small Cell Lung Carcinoma	Prexasertib + SN-38	1.000	-0.006	5.584
688025.000	NCI-H526	Lung	Small Cell Lung Carcinoma	SRA737 + SN-38	0.993	-0.033	5.584
688025.000	NCI-H526	Lung	Small Cell Lung Carcinoma	AZD1775 + SN-38	1.000	-0.004	5.584
688027.000	NCI-H69	Lung	Small Cell Lung Carcinoma	AZD1775 + SN-38	0.932	0.015	0.934
688027.000	NCI-H69	Lung	Small Cell Lung Carcinoma	SRA737 + SN-38	0.819	0.102	0.934
688027.000	NCI-H69	Lung	Small Cell Lung Carcinoma	Prexasertib + SN-38	0.907	0.202	0.934
688027.000	NCI-H69	Lung	Small Cell Lung Carcinoma	SN-38 + Olaparib	0.692	0.048	0.934
688031.000	NCI-H82	Lung	Small Cell Lung Carcinoma	SRA737 + SN-38	0.967	0.081	0.163
688031.000	NCI-H82	Lung	Small Cell Lung Carcinoma	AZD1775 + SN-38	0.988	0.022	0.163
688031.000	NCI-H82	Lung	Small Cell Lung Carcinoma	Prexasertib + SN-38	1.000	0.091	0.163
688031.000	NCI-H82	Lung	Small Cell Lung Carcinoma	SN-38 + Olaparib	0.787	0.009	0.163
688087.000	SK-N-FI	Peripheral Nervous System	Neuroblastoma	Prexasertib + SN-38	0.882	0.038	0.084
688087.000	SK-N-FI	Peripheral Nervous System	Neuroblastoma	SN-38 + Olaparib	0.518	0.054	0.084
688087.000	SK-N-FI	Peripheral Nervous System	Neuroblastoma	SRA737 + SN-38	0.606	-0.040	0.084
688087.000	SK-N-FI	Peripheral Nervous System	Neuroblastoma	AZD1775 + SN-38	0.790	0.044	0.084
688121.000	VA-ES-BJ	Soft Tissue	Other Solid Carcinomas	Prexasertib + SN-38	0.862	0.002	5.666
688121.000	VA-ES-BJ	Soft Tissue	Other Solid Carcinomas	SN-38 + Olaparib	0.818	0.003	5.666
717431.000	SK-N-SH	Peripheral Nervous System	Neuroblastoma	AZD1775 + SN-38	0.957	0.029	0.098
717431.000	SK-N-SH	Peripheral Nervous System	Neuroblastoma	SRA737 + SN-38	0.892	-0.025	0.098
717431.000	SK-N-SH	Peripheral Nervous System	Neuroblastoma	Prexasertib + SN-38	0.943	-0.030	0.098
717431.000	SK-N-SH	Peripheral Nervous System	Neuroblastoma	SN-38 + Olaparib	0.959	0.103	0.098
724825.000	NCI-H929	Haematopoietic and Lymphoid	Plasma Cell Myeloma	SN-38 + Olaparib	1.000	0.054	3.252
724825.000	NCI-H929	Haematopoietic and Lymphoid	Plasma Cell Myeloma	Prexasertib + SN-38	0.876	-0.019	3.252
724825.000	NCI-H929	Haematopoietic and Lymphoid	Plasma Cell Myeloma	SRA737 + SN-38	0.900	-0.051	3.252
724825.000	NCI-H929	Haematopoietic and Lymphoid	Plasma Cell Myeloma	SN-38 + Olaparib	0.900	0.086	3.252
724825.000	NCI-H929	Haematopoietic and Lymphoid	Plasma Cell Myeloma	Prexasertib + SN-38	0.834	-0.055	3.252
724825.000	NCI-H929	Haematopoietic and Lymphoid	Plasma Cell Myeloma	AZD1775 + SN-38	0.964	0.009	3.252
724828.000	SK-N-AS	Peripheral Nervous System	Neuroblastoma	Prexasertib + SN-38	1.000	0.052	0.070
724828.000	SK-N-AS	Peripheral Nervous System	Neuroblastoma	AZD1775 + SN-38	0.848	0.082	0.070
724828.000	SK-N-AS	Peripheral Nervous System	Neuroblastoma	SRA737 + SN-38	0.763	0.084	0.070
724828.000	SK-N-AS	Peripheral Nervous System	Neuroblastoma	SN-38 + Olaparib	0.760	0.100	0.070
724831.000	NCI-H1299	Lung	Non-Small Cell Lung Carcinoma	Prexasertib + SN-38	0.831	0.025	0.433
724831.000	NCI-H1299	Lung	Non-Small Cell Lung Carcinoma	SRA737 + SN-38	0.559	0.046	0.433
724831.000	NCI-H1299	Lung	Non-Small Cell Lung Carcinoma	SN-38 + Olaparib	0.544	0.142	0.433
724831.000	NCI-H1299	Lung	Non-Small Cell Lung Carcinoma	AZD1775 + SN-38	0.913	0.078	0.433
724834.000	NCI-H2087	Lung	Lung Adenocarcinoma	AZD1775 + SN-38	0.960	0.063	4.416
724834.000	NCI-H2087	Lung	Lung Adenocarcinoma	SRA737 + SN-38	0.918	0.055	4.416
724834.000	NCI-H2087	Lung	Lung Adenocarcinoma	Prexasertib + SN-38	0.939	0.023	4.416
724834.000	NCI-H2087	Lung	Lung Adenocarcinoma	SN-38 + Olaparib	0.909	0.025	4.416
724838.000	UM-UC-3	Bladder	Bladder Carcinoma	SN-38 + Olaparib	0.984	0.056	5.949
724838.000	UM-UC-3	Bladder	Bladder Carcinoma	AZD1775 + SN-38	0.967	0.033	5.949
724838.000	UM-UC-3	Bladder	Bladder Carcinoma	Prexasertib + SN-38	0.989	0.036	5.949
724838.000	UM-UC-3	Bladder	Bladder Carcinoma	SRA737 + SN-38	0.967	0.011	5.949
724839.000	SW756	Cervix	Cervical Carcinoma	SN-38 + Olaparib	0.766	0.006	5.641
724839.000	SW756	Cervix	Cervical Carcinoma	Prexasertib + SN-38	0.782	0.002	5.641
724855.000	NCI-H727	Lung	Other Solid Carcinomas	AZD1775 + SN-38	0.581	0.030	2.348
724855.000	NCI-H727	Lung	Other Solid Carcinomas	SN-38 + Olaparib	0.256	0.012	2.348
724855.000	NCI-H727	Lung	Other Solid Carcinomas	SRA737 + SN-38	0.609	0.082	2.348
724855.000	NCI-H727	Lung	Other Solid Carcinomas	Prexasertib + SN-38	0.679	0.001	2.348
724859.000	Calu-6	Lung	Non-Small Cell Lung Carcinoma	SN-38 + Olaparib	0.776	0.032	0.029
724859.000	Calu-6	Lung	Non-Small Cell Lung Carcinoma	Prexasertib + SN-38	0.813	0.062	0.029
724863.000	LU-65	Lung	Non-Small Cell Lung Carcinoma	SN-38 + Olaparib	0.870	0.046	0.163
724863.000	LU-65	Lung	Non-Small Cell Lung Carcinoma	Prexasertib + SN-38	0.937	0.024	0.163
724863.000	LU-65	Lung	Non-Small Cell Lung Carcinoma	SRA737 + SN-38	0.906	0.060	0.163

724863.000	LU-65	Lung	Non-Small Cell Lung Carcinoma	AZD1775 + SN-38	0.964	0.085	0.163
724866.000	NCI-H1355	Lung	Lung Adenocarcinoma	AZD1775 + SN-38	0.739	0.008	0.124
724866.000	NCI-H1355	Lung	Lung Adenocarcinoma	Prexasertib + SN-38	0.772	0.060	0.124
724866.000	NCI-H1355	Lung	Lung Adenocarcinoma	SRA737 + SN-38	0.599	0.103	0.124
724866.000	NCI-H1355	Lung	Lung Adenocarcinoma	SN-38 + Olaparib	0.366	0.040	0.124
724868.000	NCI-H1792	Lung	Lung Adenocarcinoma	AZD1775 + SN-38	0.961	0.054	1.714
724868.000	NCI-H1792	Lung	Lung Adenocarcinoma	SN-38 + Olaparib	0.575	0.052	1.714
724868.000	NCI-H1792	Lung	Lung Adenocarcinoma	SRA737 + SN-38	0.889	0.087	1.714
724868.000	NCI-H1792	Lung	Lung Adenocarcinoma	Prexasertib + SN-38	0.923	0.063	1.714
724869.000	HPAF-II	Pancreas	Pancreatic Carcinoma	AZD1775 + SN-38	0.692	0.056	0.163
724869.000	HPAF-II	Pancreas	Pancreatic Carcinoma	SRA737 + SN-38	0.548	0.033	0.163
724870.000	MIA-PaCa-2	Pancreas	Pancreatic Carcinoma	SRA737 + SN-38	0.942	0.103	0.214
724870.000	MIA-PaCa-2	Pancreas	Pancreatic Carcinoma	AZD1775 + SN-38	0.983	0.140	0.214
724870.000	MIA-PaCa-2	Pancreas	Pancreatic Carcinoma	SN-38 + Olaparib	0.935	0.081	0.214
724870.000	MIA-PaCa-2	Pancreas	Pancreatic Carcinoma	Prexasertib + SN-38	1.000	0.071	0.214
724872.000	SHP-77	Lung	Small Cell Lung Carcinoma	SN-38 + Olaparib	0.567	0.048	0.084
724872.000	SHP-77	Lung	Small Cell Lung Carcinoma	Prexasertib + SN-38	0.984	0.138	0.084
724872.000	SHP-77	Lung	Small Cell Lung Carcinoma	SRA737 + SN-38	0.912	0.161	0.084
724872.000	SHP-77	Lung	Small Cell Lung Carcinoma	AZD1775 + SN-38	0.979	0.022	0.084
724873.000	NCI-H2009	Lung	Lung Adenocarcinoma	Prexasertib + SN-38	0.885	0.163	0.872
724873.000	NCI-H2009	Lung	Lung Adenocarcinoma	SRA737 + SN-38	0.768	0.082	0.872
724873.000	NCI-H2009	Lung	Lung Adenocarcinoma	SN-38 + Olaparib	0.442	0.155	0.872
724873.000	NCI-H2009	Lung	Lung Adenocarcinoma	AZD1775 + SN-38	0.988	0.167	0.872
735784.000	TE-5	Esophagus	Esophageal Squamous Cell Carcinoma	SN-38 + Olaparib	0.758	0.069	4.818
735784.000	TE-5	Esophagus	Esophageal Squamous Cell Carcinoma	Prexasertib + SN-38	0.824	0.014	4.818
735784.000	TE-5	Esophagus	Esophageal Squamous Cell Carcinoma	SRA737 + SN-38	0.851	-0.034	4.818
735784.000	TE-5	Esophagus	Esophageal Squamous Cell Carcinoma	AZD1775 + SN-38	0.876	-0.006	4.818
749711.000	HCC1187	Breast	Breast Carcinoma	SN-38 + Olaparib	0.467	0.056	2.901
749711.000	HCC1187	Breast	Breast Carcinoma	Prexasertib + SN-38	0.877	0.179	2.901
749712.000	HCC1395	Breast	Breast Carcinoma	SN-38 + Olaparib	0.500	0.096	0.111
749712.000	HCC1395	Breast	Breast Carcinoma	SN-38 + Olaparib	0.536	0.025	0.111
749712.000	HCC1395	Breast	Breast Carcinoma	Prexasertib + SN-38	0.907	0.025	0.111
749712.000	HCC1395	Breast	Breast Carcinoma	SRA737 + SN-38	0.766	0.076	0.111
749712.000	HCC1395	Breast	Breast Carcinoma	Prexasertib + SN-38	0.898	0.018	0.111
749712.000	HCC1395	Breast	Breast Carcinoma	AZD1775 + SN-38	0.832	0.058	0.111
749717.000	HCC38	Breast	Breast Carcinoma	SRA737 + SN-38	0.682	0.018	5.301
749717.000	HCC38	Breast	Breast Carcinoma	Prexasertib + SN-38	0.784	0.053	5.301
749717.000	HCC38	Breast	Breast Carcinoma	SN-38 + Olaparib	0.682	0.050	5.301
749717.000	HCC38	Breast	Breast Carcinoma	AZD1775 + SN-38	0.751	0.030	5.301
753539.000	CADO-ES1	Bone	Ewing's Sarcoma	SN-38 + Olaparib	0.987	0.030	5.087
753539.000	CADO-ES1	Bone	Ewing's Sarcoma	Prexasertib + SN-38	0.992	-0.010	5.087
753541.000	CAL-33	Head and Neck	Oral Cavity Carcinoma	Prexasertib + SN-38	0.942	-0.004	5.019
753541.000	CAL-33	Head and Neck	Oral Cavity Carcinoma	SN-38 + Olaparib	0.962	0.064	5.019
753556.000	EPLC-272H	Lung	Other Solid Carcinomas	SN-38 + Olaparib	0.856	0.075	4.910
753556.000	EPLC-272H	Lung	Other Solid Carcinomas	Prexasertib + SN-38	0.932	0.010	4.910
753562.000	HSC-2	Head and Neck	Oral Cavity Carcinoma	SRA737 + SN-38	0.960	-0.041	3.842
753562.000	HSC-2	Head and Neck	Oral Cavity Carcinoma	SN-38 + Olaparib	0.977	0.109	3.842
753562.000	HSC-2	Head and Neck	Oral Cavity Carcinoma	AZD1775 + SN-38	0.952	0.008	3.842
753562.000	HSC-2	Head and Neck	Oral Cavity Carcinoma	Prexasertib + SN-38	0.985	-0.015	3.842
753566.000	J82	Bladder	Bladder Carcinoma	Prexasertib + SN-38	0.814	0.027	4.695
753566.000	J82	Bladder	Bladder Carcinoma	SN-38 + Olaparib	0.861	0.078	4.695
753569.000	KNS-62	Lung	Squamous Cell Lung Carcinoma	Prexasertib + SN-38	0.895	0.007	2.838
753569.000	KNS-62	Lung	Squamous Cell Lung Carcinoma	AZD1775 + SN-38	0.866	0.046	2.838
753569.000	KNS-62	Lung	Squamous Cell Lung Carcinoma	SN-38 + Olaparib	0.905	0.066	2.838
753569.000	KNS-62	Lung	Squamous Cell Lung Carcinoma	SRA737 + SN-38	0.866	-0.007	2.838
753572.000	KP-4	Pancreas	Pancreatic Carcinoma	AZD1775 + SN-38	0.961	0.057	4.904
753572.000	KP-4	Pancreas	Pancreatic Carcinoma	SN-38 + Olaparib	0.817	0.090	4.904
753572.000	KP-4	Pancreas	Pancreatic Carcinoma	Prexasertib + SN-38	0.902	0.062	4.904
753572.000	KP-4	Pancreas	Pancreatic Carcinoma	SRA737 + SN-38	0.925	-0.019	4.904
753574.000	KYSE-410	Esophagus	Esophageal Squamous Cell Carcinoma	Prexasertib + SN-38	0.556	0.017	0.070
753574.000	KYSE-410	Esophagus	Esophageal Squamous Cell Carcinoma	SN-38 + Olaparib	0.423	0.015	0.070
753576.000	KYSE-70	Esophagus	Esophageal Squamous Cell Carcinoma	SN-38 + Olaparib	0.305	0.048	0.029
753576.000	KYSE-70	Esophagus	Esophageal Squamous Cell Carcinoma	SRA737 + SN-38	0.683	0.108	0.029
753576.000	KYSE-70	Esophagus	Esophageal Squamous Cell Carcinoma	AZD1775 + SN-38	0.835	0.023	0.029
753576.000	KYSE-70	Esophagus	Esophageal Squamous Cell Carcinoma	Prexasertib + SN-38	0.879	0.092	0.029
753586.000	LCLC-103H	Lung	Non-Small Cell Lung Carcinoma	AZD1775 + SN-38	0.896	0.072	3.575
753586.000	LCLC-103H	Lung	Non-Small Cell Lung Carcinoma	SRA737 + SN-38	0.817	0.087	3.575
753586.000	LCLC-103H	Lung	Non-Small Cell Lung Carcinoma	SN-38 + Olaparib	0.540	0.057	3.575
753586.000	LCLC-103H	Lung	Non-Small Cell Lung Carcinoma	Prexasertib + SN-38	0.955	0.103	3.575
753608.000	PC-14	Lung	Lung Adenocarcinoma	Prexasertib + SN-38	0.908	0.039	0.390
753608.000	PC-14	Lung	Lung Adenocarcinoma	AZD1775 + SN-38	0.972	0.077	0.390
753608.000	PC-14	Lung	Lung Adenocarcinoma	SRA737 + SN-38	0.807	0.046	0.390
753608.000	PC-14	Lung	Lung Adenocarcinoma	AZD1775 + SN-38	0.967	0.001	0.390
753608.000	PC-14	Lung	Lung Adenocarcinoma	AZD1775 + SN-38	0.954	0.060	0.390
753608.000	PC-14	Lung	Lung Adenocarcinoma	SN-38 + Olaparib	0.619	0.045	0.390
753608.000	PC-14	Lung	Lung Adenocarcinoma	SRA737 + SN-38	0.816	0.082	0.390
753608.000	PC-14	Lung	Lung Adenocarcinoma	SN-38 + Olaparib	0.622	0.069	0.390
753608.000	PC-14	Lung	Lung Adenocarcinoma	Prexasertib + SN-38	0.936	0.033	0.390
753608.000	PC-14	Lung	Lung Adenocarcinoma	SN-38 + Olaparib	0.777	0.083	0.390
753608.000	PC-14	Lung	Lung Adenocarcinoma	SN-38 + Olaparib	0.742	0.106	0.390
753608.000	PC-14	Lung	Lung Adenocarcinoma	Prexasertib + SN-38	0.900	0.001	0.390
753608.000	PC-14	Lung	Lung Adenocarcinoma	SN-38 + Olaparib	0.652	0.058	0.390
753608.000	PC-14	Lung	Lung Adenocarcinoma	SN-38 + Olaparib	0.712	0.094	0.390
753608.000	PC-14	Lung	Lung Adenocarcinoma	Prexasertib + SN-38	0.885	0.018	0.390
753608.000	PC-14	Lung	Lung Adenocarcinoma	AZD1775 + SN-38	0.980	0.051	0.390
753608.000	PC-14	Lung	Lung Adenocarcinoma	Prexasertib + SN-38	0.893	0.002	0.390
753608.000	PC-14	Lung	Lung Adenocarcinoma	Prexasertib + SN-38	0.912	0.012	0.390
753608.000	PC-14	Lung	Lung Adenocarcinoma	SN-38 + Olaparib	0.706	0.098	0.390
753608.000	PC-14	Lung	Lung Adenocarcinoma	Prexasertib + SN-38	0.933	0.051	0.390
753608.000	PC-14	Lung	Lung Adenocarcinoma	SRA737 + SN-38	0.889	0.065	0.390
753608.000	PC-14	Lung	Lung Adenocarcinoma	SRA737 + SN-38	0.878	0.074	0.390
753608.000	PC-14	Lung	Lung Adenocarcinoma	SN-38 + Olaparib	0.706	0.045	0.390
753608.000	PC-14	Lung	Lung Adenocarcinoma	AZD1775 + SN-38	0.985	0.022	0.390
753608.000	PC-14	Lung	Lung Adenocarcinoma	AZD1775 + SN-38	0.933	0.021	0.390
753608.000	PC-14	Lung	Lung Adenocarcinoma	Prexasertib + SN-38	0.882	0.037	0.390
753608.000	PC-14	Lung	Lung Adenocarcinoma	SRA737 + SN-38	0.826	0.078	0.390
753608.000	PC-14	Lung	Lung Adenocarcinoma	Prexasertib + SN-38	0.898	-0.012	0.390
753608.000	PC-14	Lung	Lung Adenocarcinoma	Prexasertib + SN-38	0.881	0.013	0.390
753608.000	PC-14	Lung	Lung Adenocarcinoma	AZD1775 + SN-38	0.964	0.051	0.390
753608.000	PC-14	Lung	Lung Adenocarcinoma	SN-38 + Olaparib	0.658	0.132	0.390
753608.000	PC-14	Lung	Lung Adenocarcinoma	SN-38 + Olaparib	0.696	0.049	0.390
753608.000	PC-14	Lung	Lung Adenocarcinoma	SRA737 + SN-38	0.793	0.026	0.390
753608.000	PC-14	Lung	Lung Adenocarcinoma	Prexasertib + SN-38	0.883	-0.013	0.390
753608.000	PC-14	Lung	Lung Adenocarcinoma	AZD1775 + SN-38	0.946	0.034	0.390
753608.000	PC-14	Lung	Lung Adenocarcinoma	SN-38 + Olaparib	0.767	0.045	0.390

753608.000	PC-14	Lung	Lung Adenocarcinoma	SN-38 + Olaparib	0.634	0.086	0.390
753608.000	PC-14	Lung	Lung Adenocarcinoma	Prexasertib + SN-38	0.928	0.040	0.390
753608.000	PC-14	Lung	Lung Adenocarcinoma	SRA737 + SN-38	0.796	0.056	0.390
753608.000	PC-14	Lung	Lung Adenocarcinoma	SRA737 + SN-38	0.897	0.069	0.390
753608.000	PC-14	Lung	Lung Adenocarcinoma	Prexasertib + SN-38	0.869	-0.015	0.390
753608.000	PC-14	Lung	Lung Adenocarcinoma	SN-38 + Olaparib	0.643	0.103	0.390
753608.000	PC-14	Lung	Lung Adenocarcinoma	SRA737 + SN-38	0.755	0.089	0.390
753608.000	PC-14	Lung	Lung Adenocarcinoma	AZD1775 + SN-38	0.945	0.023	0.390
753608.000	PC-14	Lung	Lung Adenocarcinoma	SN-38 + Olaparib	0.602	0.092	0.390
753608.000	PC-14	Lung	Lung Adenocarcinoma	Prexasertib + SN-38	0.876	0.009	0.390
753608.000	PC-14	Lung	Lung Adenocarcinoma	Prexasertib + SN-38	0.860	0.026	0.390
753608.000	PC-14	Lung	Lung Adenocarcinoma	SN-38 + Olaparib	0.723	0.044	0.390
753612.000	SK-MM-2	Haematopoietic and Lymphoid	Plasma Cell Myeloma	SRA737 + SN-38	0.776	0.027	4.464
753612.000	SK-MM-2	Haematopoietic and Lymphoid	Plasma Cell Myeloma	Prexasertib + SN-38	0.876	-0.012	4.464
753612.000	SK-MM-2	Haematopoietic and Lymphoid	Plasma Cell Myeloma	SN-38 + Olaparib	0.890	0.006	4.464
753612.000	SK-MM-2	Haematopoietic and Lymphoid	Plasma Cell Myeloma	AZD1775 + SN-38	0.794	0.055	4.464
753614.000	TE-15	Esophagus	Esophageal Squamous Cell Carcinoma	SRA737 + SN-38	0.931	-0.021	4.886
753614.000	TE-15	Esophagus	Esophageal Squamous Cell Carcinoma	Prexasertib + SN-38	0.936	0.052	4.886
753614.000	TE-15	Esophagus	Esophageal Squamous Cell Carcinoma	SN-38 + Olaparib	0.811	0.043	4.886
753614.000	TE-15	Esophagus	Esophageal Squamous Cell Carcinoma	AZD1775 + SN-38	0.939	0.050	4.886
753615.000	U-266	Haematopoietic and Lymphoid	Plasma Cell Myeloma	SN-38 + Olaparib	0.367	0.066	2.278
753615.000	U-266	Haematopoietic and Lymphoid	Plasma Cell Myeloma	SRA737 + SN-38	0.576	0.086	2.278
753615.000	U-266	Haematopoietic and Lymphoid	Plasma Cell Myeloma	Prexasertib + SN-38	0.713	-0.019	2.278
753615.000	U-266	Haematopoietic and Lymphoid	Plasma Cell Myeloma	AZD1775 + SN-38	0.914	0.017	2.278
753618.000	KELLY	Peripheral Nervous System	Neuroblastoma	AZD1775 + SN-38	1.000	0.006	0.029
753618.000	KELLY	Peripheral Nervous System	Neuroblastoma	SRA737 + SN-38	1.000	0.004	0.029
753618.000	KELLY	Peripheral Nervous System	Neuroblastoma	Prexasertib + SN-38	1.000	0.012	0.029
753618.000	KELLY	Peripheral Nervous System	Neuroblastoma	SN-38 + Olaparib	0.884	0.058	0.029
753622.000	TE-10	Esophagus	Esophageal Squamous Cell Carcinoma	Prexasertib + SN-38	0.933	0.035	2.531
753622.000	TE-10	Esophagus	Esophageal Squamous Cell Carcinoma	Prexasertib + SN-38	0.973	-0.010	2.531
753622.000	TE-10	Esophagus	Esophageal Squamous Cell Carcinoma	SN-38 + Olaparib	0.769	0.125	2.531
753622.000	TE-10	Esophagus	Esophageal Squamous Cell Carcinoma	Prexasertib + SN-38	0.945	0.010	2.531
753622.000	TE-10	Esophagus	Esophageal Squamous Cell Carcinoma	SN-38 + Olaparib	0.757	0.090	2.531
753622.000	TE-10	Esophagus	Esophageal Squamous Cell Carcinoma	Prexasertib + SN-38	0.943	0.009	2.531
753622.000	TE-10	Esophagus	Esophageal Squamous Cell Carcinoma	SN-38 + Olaparib	0.752	0.079	2.531
753622.000	TE-10	Esophagus	Esophageal Squamous Cell Carcinoma	SN-38 + Olaparib	0.743	0.101	2.531
753622.000	TE-10	Esophagus	Esophageal Squamous Cell Carcinoma	Prexasertib + SN-38	0.934	0.007	2.531
753622.000	TE-10	Esophagus	Esophageal Squamous Cell Carcinoma	SN-38 + Olaparib	0.746	0.112	2.531
753623.000	TE-8	Esophagus	Esophageal Squamous Cell Carcinoma	SRA737 + SN-38	0.838	0.081	1.709
753623.000	TE-8	Esophagus	Esophageal Squamous Cell Carcinoma	AZD1775 + SN-38	0.932	0.163	1.709
753623.000	TE-8	Esophagus	Esophageal Squamous Cell Carcinoma	SN-38 + Olaparib	0.575	0.036	1.709
753623.000	TE-8	Esophagus	Esophageal Squamous Cell Carcinoma	Prexasertib + SN-38	0.972	0.085	1.709
753624.000	CAPAN-1	Pancreas	Pancreatic Carcinoma	SN-38 + Olaparib	0.818	0.043	4.552
753624.000	CAPAN-1	Pancreas	Pancreatic Carcinoma	SRA737 + SN-38	0.758	-0.017	4.552
753624.000	CAPAN-1	Pancreas	Pancreatic Carcinoma	AZD1775 + SN-38	0.825	0.048	4.552
753624.000	CAPAN-1	Pancreas	Pancreatic Carcinoma	Prexasertib + SN-38	0.844	-0.009	4.552
905933.000	OVCAR-3	Ovary	Ovarian Carcinoma	Prexasertib + SN-38	0.948	0.185	1.411
905933.000	OVCAR-3	Ovary	Ovarian Carcinoma	SN-38 + Olaparib	0.687	0.109	1.411
905935.000	DU-145	Prostate	Prostate Carcinoma	AZD1775 + SN-38	0.735	0.002	5.501
905935.000	DU-145	Prostate	Prostate Carcinoma	SRA737 + SN-38	0.628	-0.010	5.501
905935.000	DU-145	Prostate	Prostate Carcinoma	SN-38 + Olaparib	0.813	0.041	5.501
905935.000	DU-145	Prostate	Prostate Carcinoma	Prexasertib + SN-38	0.821	0.013	5.501
905936.000	HCT-116	Large Intestine	Colorectal Carcinoma	SN-38 + Olaparib	0.900	0.128	0.070
905936.000	HCT-116	Large Intestine	Colorectal Carcinoma	Prexasertib + SN-38	0.905	0.042	0.070
905936.000	HCT-116	Large Intestine	Colorectal Carcinoma	AZD1775 + SN-38	0.945	0.051	0.070
905936.000	HCT-116	Large Intestine	Colorectal Carcinoma	SRA737 + SN-38	0.818	0.000	0.070
905937.000	HCT-15	Large Intestine	Colorectal Carcinoma	SN-38 + Olaparib	0.851	0.083	0.084
905937.000	HCT-15	Large Intestine	Colorectal Carcinoma	Prexasertib + SN-38	0.997	0.045	0.084
905939.000	HT-29	Large Intestine	Colorectal Carcinoma	Prexasertib + SN-38	0.944	0.001	0.043
905939.000	HT-29	Large Intestine	Colorectal Carcinoma	SRA737 + SN-38	0.877	0.109	0.043
905939.000	HT-29	Large Intestine	Colorectal Carcinoma	SN-38 + Olaparib	0.984	0.061	0.043
905939.000	HT-29	Large Intestine	Colorectal Carcinoma	SN-38 + Olaparib	0.936	0.068	0.043
905939.000	HT-29	Large Intestine	Colorectal Carcinoma	Prexasertib + SN-38	0.900	0.063	0.043
905939.000	HT-29	Large Intestine	Colorectal Carcinoma	Prexasertib + SN-38	0.964	0.018	0.043
905939.000	HT-29	Large Intestine	Colorectal Carcinoma	AZD1775 + SN-38	0.905	0.091	0.043
905939.000	HT-29	Large Intestine	Colorectal Carcinoma	Prexasertib + SN-38	0.936	0.024	0.043
905939.000	HT-29	Large Intestine	Colorectal Carcinoma	SN-38 + Olaparib	0.961	0.046	0.043
905939.000	HT-29	Large Intestine	Colorectal Carcinoma	SN-38 + Olaparib	0.953	0.020	0.043
905939.000	HT-29	Large Intestine	Colorectal Carcinoma	SRA737 + SN-38	0.853	0.128	0.043
905939.000	HT-29	Large Intestine	Colorectal Carcinoma	AZD1775 + SN-38	0.930	0.062	0.043
905939.000	HT-29	Large Intestine	Colorectal Carcinoma	SRA737 + SN-38	0.884	0.110	0.043
905939.000	HT-29	Large Intestine	Colorectal Carcinoma	SN-38 + Olaparib	0.881	0.020	0.043
905939.000	HT-29	Large Intestine	Colorectal Carcinoma	SN-38 + Olaparib	0.988	0.067	0.043
905939.000	HT-29	Large Intestine	Colorectal Carcinoma	AZD1775 + SN-38	0.905	0.017	0.043
905939.000	HT-29	Large Intestine	Colorectal Carcinoma	SN-38 + Olaparib	0.905	0.035	0.043
905939.000	HT-29	Large Intestine	Colorectal Carcinoma	SN-38 + Olaparib	0.853	0.066	0.043
905939.000	HT-29	Large Intestine	Colorectal Carcinoma	Prexasertib + SN-38	0.910	0.002	0.043
905939.000	HT-29	Large Intestine	Colorectal Carcinoma	Prexasertib + SN-38	0.909	0.050	0.043
905939.000	HT-29	Large Intestine	Colorectal Carcinoma	Prexasertib + SN-38	0.901	0.009	0.043
905939.000	HT-29	Large Intestine	Colorectal Carcinoma	SN-38 + Olaparib	0.953	0.051	0.043
905939.000	HT-29	Large Intestine	Colorectal Carcinoma	Prexasertib + SN-38	0.915	0.003	0.043
905939.000	HT-29	Large Intestine	Colorectal Carcinoma	SN-38 + Olaparib	0.956	0.077	0.043
905939.000	HT-29	Large Intestine	Colorectal Carcinoma	Prexasertib + SN-38	0.945	0.020	0.043
905939.000	HT-29	Large Intestine	Colorectal Carcinoma	Prexasertib + SN-38	0.881	0.013	0.043
905939.000	HT-29	Large Intestine	Colorectal Carcinoma	Prexasertib + SN-38	0.962	0.017	0.043
905939.000	HT-29	Large Intestine	Colorectal Carcinoma	SN-38 + Olaparib	0.843	0.027	0.043
905939.000	HT-29	Large Intestine	Colorectal Carcinoma	Prexasertib + SN-38	0.887	0.012	0.043
905939.000	HT-29	Large Intestine	Colorectal Carcinoma	SN-38 + Olaparib	0.874	0.036	0.043
905939.000	HT-29	Large Intestine	Colorectal Carcinoma	Prexasertib + SN-38	0.877	0.015	0.043
905939.000	HT-29	Large Intestine	Colorectal Carcinoma	SN-38 + Olaparib	0.872	0.030	0.043
905939.000	HT-29	Large Intestine	Colorectal Carcinoma	Prexasertib + SN-38	0.967	0.004	0.043
905939.000	HT-29	Large Intestine	Colorectal Carcinoma	Prexasertib + SN-38	0.911	-0.020	0.043
905939.000	HT-29	Large Intestine	Colorectal Carcinoma	SN-38 + Olaparib	0.889	0.070	0.043
905939.000	HT-29	Large Intestine	Colorectal Carcinoma	SN-38 + Olaparib	0.955	0.114	0.043
905940.000	K-562	Haematopoietic and Lymphoid	Chronic Myelogenous Leukemia	SRA737 + SN-38	0.977	0.176	0.043
905940.000	K-562	Haematopoietic and Lymphoid	Chronic Myelogenous Leukemia	Prexasertib + SN-38	0.991	0.095	0.043
905940.000	K-562	Haematopoietic and Lymphoid	Chronic Myelogenous Leukemia	AZD1775 + SN-38	0.966	0.102	0.043
905940.000	K-562	Haematopoietic and Lymphoid	Chronic Myelogenous Leukemia	SN-38 + Olaparib	0.672	0.032	0.043
905941.000	NCI-H226	Lung	Mesothelioma	Prexasertib + SN-38	0.459	-0.017	4.445
905941.000	NCI-H226	Lung	Mesothelioma	SN-38 + Olaparib	0.168	-0.027	4.445
905941.000	NCI-H226	Lung	Mesothelioma	AZD1775 + SN-38	0.622	-0.013	4.445
905941.000	NCI-H226	Lung	Mesothelioma	SRA737 + SN-38	0.248	-0.038	4.445
905944.000	NCI-H522	Lung	Lung Adenocarcinoma	Prexasertib + SN-38	0.807	0.170	0.124
905944.000	NCI-H522	Lung	Lung Adenocarcinoma	SRA737 + SN-38	0.635	0.056	0.124
905944.000	NCI-H522	Lung	Lung Adenocarcinoma	AZD1775 + SN-38	0.906	0.148	0.124

905944.000	NCI-H522	Lung	Lung Adenocarcinoma	SN-38 + Olaparib	0.389	0.102	0.124
905945.000	T47D	Breast	Breast Carcinoma	Prexasertib + SN-38	0.696	0.005	4.485
905945.000	T47D	Breast	Breast Carcinoma	SN-38 + Olaparib	0.625	0.085	4.485
905946.000	MCF7	Breast	Breast Carcinoma	SN-38 + Olaparib	0.655	0.028	0.111
905946.000	MCF7	Breast	Breast Carcinoma	Prexasertib + SN-38	0.693	0.023	0.111
905947.000	786-0	Kidney	Kidney Carcinoma	SN-38 + Olaparib	0.931	0.102	5.715
905947.000	786-0	Kidney	Kidney Carcinoma	Prexasertib + SN-38	0.942	-0.022	5.715
905948.000	A498	Kidney	Kidney Carcinoma	SN-38 + Olaparib	0.598	0.059	0.163
905948.000	A498	Kidney	Kidney Carcinoma	Prexasertib + SN-38	0.598	-0.044	0.163
905949.000	A549	Lung	Lung Adenocarcinoma	SN-38 + Olaparib	0.817	0.022	3.801
905949.000	A549	Lung	Lung Adenocarcinoma	Prexasertib + SN-38	0.786	-0.001	3.801
905950.000	ACHN	Kidney	Kidney Carcinoma	Prexasertib + SN-38	0.949	-0.020	6.279
905950.000	ACHN	Kidney	Kidney Carcinoma	AZD1775 + SN-38	0.953	0.013	6.279
905950.000	ACHN	Kidney	Kidney Carcinoma	SRA737 + SN-38	0.893	-0.008	6.279
905950.000	ACHN	Kidney	Kidney Carcinoma	SN-38 + Olaparib	0.912	0.029	6.279
905951.000	BT-549	Breast	Breast Carcinoma	SN-38 + Olaparib	0.636	0.080	4.355
905951.000	BT-549	Breast	Breast Carcinoma	AZD1775 + SN-38	0.875	0.056	4.355
905951.000	BT-549	Breast	Breast Carcinoma	Prexasertib + SN-38	0.849	0.019	4.355
905951.000	BT-549	Breast	Breast Carcinoma	SRA737 + SN-38	0.750	0.023	4.355
905955.000	SK-MEL-2	Skin	Melanoma	SRA737 + SN-38	0.693	0.101	0.000
905955.000	SK-MEL-2	Skin	Melanoma	AZD1775 + SN-38	0.898	0.072	0.000
905957.000	Hs-578-T	Breast	Breast Carcinoma	AZD1775 + SN-38	0.883	0.113	0.098
905957.000	Hs-578-T	Breast	Breast Carcinoma	SN-38 + Olaparib	0.709	0.098	0.098
905957.000	Hs-578-T	Breast	Breast Carcinoma	Prexasertib + SN-38	0.989	0.110	0.098
905957.000	Hs-578-T	Breast	Breast Carcinoma	SRA737 + SN-38	0.804	0.153	0.098
905959.000	SK-OV-3	Ovary	Ovarian Carcinoma	Prexasertib + SN-38	0.856	0.003	5.153
905959.000	SK-OV-3	Ovary	Ovarian Carcinoma	SN-38 + Olaparib	0.535	0.076	5.153
905959.000	SK-OV-3	Ovary	Ovarian Carcinoma	SRA737 + SN-38	0.542	0.026	5.153
905959.000	SK-OV-3	Ovary	Ovarian Carcinoma	AZD1775 + SN-38	0.594	0.064	5.153
905960.000	MDA-MB-231	Breast	Breast Carcinoma	SRA737 + SN-38	0.908	0.012	0.163
905960.000	MDA-MB-231	Breast	Breast Carcinoma	AZD1775 + SN-38	0.943	0.091	0.163
905960.000	MDA-MB-231	Breast	Breast Carcinoma	Prexasertib + SN-38	0.976	0.090	0.163
905960.000	MDA-MB-231	Breast	Breast Carcinoma	SN-38 + Olaparib	0.681	0.128	0.163
905962.000	SW620	Large Intestine	Colorectal Carcinoma	SN-38 + Olaparib	0.763	0.076	0.057
905962.000	SW620	Large Intestine	Colorectal Carcinoma	SRA737 + SN-38	0.964	0.084	0.057
905962.000	SW620	Large Intestine	Colorectal Carcinoma	SRA737 + SN-38	0.964	0.076	0.057
905962.000	SW620	Large Intestine	Colorectal Carcinoma	SRA737 + SN-38	0.940	0.028	0.057
905962.000	SW620	Large Intestine	Colorectal Carcinoma	AZD1775 + SN-38	1.000	0.080	0.057
905962.000	SW620	Large Intestine	Colorectal Carcinoma	Prexasertib + SN-38	0.990	0.112	0.057
905962.000	SW620	Large Intestine	Colorectal Carcinoma	Prexasertib + SN-38	0.962	0.097	0.057
905962.000	SW620	Large Intestine	Colorectal Carcinoma	SN-38 + Olaparib	0.823	0.063	0.057
905962.000	SW620	Large Intestine	Colorectal Carcinoma	AZD1775 + SN-38	0.995	0.064	0.057
905962.000	SW620	Large Intestine	Colorectal Carcinoma	SN-38 + Olaparib	0.840	0.029	0.057
905962.000	SW620	Large Intestine	Colorectal Carcinoma	Prexasertib + SN-38	0.982	0.100	0.057
905962.000	SW620	Large Intestine	Colorectal Carcinoma	SRA737 + SN-38	0.958	0.062	0.057
905962.000	SW620	Large Intestine	Colorectal Carcinoma	SN-38 + Olaparib	0.822	0.043	0.057
905962.000	SW620	Large Intestine	Colorectal Carcinoma	Prexasertib + SN-38	0.981	0.102	0.057
905962.000	SW620	Large Intestine	Colorectal Carcinoma	Prexasertib + SN-38	0.997	0.038	0.057
905962.000	SW620	Large Intestine	Colorectal Carcinoma	SN-38 + Olaparib	0.762	0.044	0.057
905962.000	SW620	Large Intestine	Colorectal Carcinoma	AZD1775 + SN-38	0.994	0.093	0.057
905962.000	SW620	Large Intestine	Colorectal Carcinoma	Prexasertib + SN-38	0.971	0.097	0.057
905962.000	SW620	Large Intestine	Colorectal Carcinoma	SN-38 + Olaparib	0.843	0.028	0.057
905962.000	SW620	Large Intestine	Colorectal Carcinoma	Prexasertib + SN-38	0.974	0.095	0.057
905962.000	SW620	Large Intestine	Colorectal Carcinoma	SN-38 + Olaparib	0.745	0.083	0.057
905962.000	SW620	Large Intestine	Colorectal Carcinoma	SN-38 + Olaparib	0.880	0.110	0.057
905962.000	SW620	Large Intestine	Colorectal Carcinoma	SRA737 + SN-38	0.952	0.086	0.057
905962.000	SW620	Large Intestine	Colorectal Carcinoma	SN-38 + Olaparib	0.849	0.044	0.057
905962.000	SW620	Large Intestine	Colorectal Carcinoma	Prexasertib + SN-38	0.959	0.079	0.057
905962.000	SW620	Large Intestine	Colorectal Carcinoma	Prexasertib + SN-38	0.949	0.128	0.057
905962.000	SW620	Large Intestine	Colorectal Carcinoma	SN-38 + Olaparib	0.705	0.049	0.057
905962.000	SW620	Large Intestine	Colorectal Carcinoma	Prexasertib + SN-38	0.944	0.075	0.057
905962.000	SW620	Large Intestine	Colorectal Carcinoma	AZD1775 + SN-38	0.992	0.097	0.057
905962.000	SW620	Large Intestine	Colorectal Carcinoma	SN-38 + Olaparib	0.788	0.051	0.057
905962.000	SW620	Large Intestine	Colorectal Carcinoma	SN-38 + Olaparib	0.866	0.105	0.057
905962.000	SW620	Large Intestine	Colorectal Carcinoma	Prexasertib + SN-38	0.976	0.055	0.057
905962.000	SW620	Large Intestine	Colorectal Carcinoma	SN-38 + Olaparib	0.846	0.078	0.057
905962.000	SW620	Large Intestine	Colorectal Carcinoma	AZD1775 + SN-38	1.000	0.076	0.057
905962.000	SW620	Large Intestine	Colorectal Carcinoma	Prexasertib + SN-38	0.963	0.107	0.057
905962.000	SW620	Large Intestine	Colorectal Carcinoma	SN-38 + Olaparib	0.798	0.109	0.057
905962.000	SW620	Large Intestine	Colorectal Carcinoma	AZD1775 + SN-38	1.000	0.073	0.057
905962.000	SW620	Large Intestine	Colorectal Carcinoma	SN-38 + Olaparib	0.731	0.105	0.057
905962.000	SW620	Large Intestine	Colorectal Carcinoma	SRA737 + SN-38	0.942	0.043	0.057
905962.000	SW620	Large Intestine	Colorectal Carcinoma	Prexasertib + SN-38	0.977	0.140	0.057
905962.000	SW620	Large Intestine	Colorectal Carcinoma	Prexasertib + SN-38	1.000	0.054	0.057
905962.000	SW620	Large Intestine	Colorectal Carcinoma	Prexasertib + SN-38	0.967	0.089	0.057
905962.000	SW620	Large Intestine	Colorectal Carcinoma	Prexasertib + SN-38	0.954	0.091	0.057
905962.000	SW620	Large Intestine	Colorectal Carcinoma	SN-38 + Olaparib	0.829	0.064	0.057
905962.000	SW620	Large Intestine	Colorectal Carcinoma	AZD1775 + SN-38	0.988	0.084	0.057
905962.000	SW620	Large Intestine	Colorectal Carcinoma	SRA737 + SN-38	0.970	0.058	0.057
905962.000	SW620	Large Intestine	Colorectal Carcinoma	AZD1775 + SN-38	0.993	0.059	0.057
905962.000	SW620	Large Intestine	Colorectal Carcinoma	SRA737 + SN-38	0.932	0.097	0.057
905962.000	SW620	Large Intestine	Colorectal Carcinoma	Prexasertib + SN-38	0.972	0.090	0.057
905962.000	SW620	Large Intestine	Colorectal Carcinoma	SN-38 + Olaparib	0.732	0.074	0.057
905965.000	SR	Haematopoietic and Lymphoid	Other Blood Carcinomas	Prexasertib + SN-38	0.996	-0.024	5.147
905965.000	SR	Haematopoietic and Lymphoid	Other Blood Carcinomas	SN-38 + Olaparib	0.982	0.072	5.147
905965.000	SR	Haematopoietic and Lymphoid	Other Blood Carcinomas	AZD1775 + SN-38	1.000	0.042	5.147
905965.000	SR	Haematopoietic and Lymphoid	Other Blood Carcinomas	SRA737 + SN-38	1.000	0.031	5.147
905970.000	EKVX	Lung	Non-Small Cell Lung Carcinoma	SN-38 + Olaparib	0.293	0.007	0.322
905970.000	EKVX	Lung	Non-Small Cell Lung Carcinoma	SN-38 + Olaparib	0.306	0.023	0.322
905970.000	EKVX	Lung	Non-Small Cell Lung Carcinoma	Prexasertib + SN-38	0.382	-0.058	0.322
905970.000	EKVX	Lung	Non-Small Cell Lung Carcinoma	Prexasertib + SN-38	0.379	-0.006	0.322
905972.000	HOP-62	Lung	Lung Adenocarcinoma	SN-38 + Olaparib	0.672	0.072	5.581
905972.000	HOP-62	Lung	Lung Adenocarcinoma	Prexasertib + SN-38	0.819	0.068	5.581
905974.000	LOXIMVI	Skin	Melanoma	SN-38 + Olaparib	0.784	0.048	4.250
905974.000	LOXIMVI	Skin	Melanoma	Prexasertib + SN-38	0.868	-0.001	4.250
905974.000	LOXIMVI	Skin	Melanoma	AZD1775 + SN-38	0.863	-0.007	4.250
905974.000	LOXIMVI	Skin	Melanoma	SRA737 + SN-38	0.859	0.024	4.250
905976.000	UACC-62	Skin	Melanoma	Prexasertib + SN-38	0.867	-0.040	3.783
905976.000	UACC-62	Skin	Melanoma	AZD1775 + SN-38	0.745	0.024	3.783
905976.000	UACC-62	Skin	Melanoma	SN-38 + Olaparib	0.843	0.039	3.783
905976.000	UACC-62	Skin	Melanoma	SRA737 + SN-38	0.664	0.090	3.783
905977.000	UACC-257	Skin	Melanoma	Prexasertib + SN-38	0.327	-0.044	4.061
905977.000	UACC-257	Skin	Melanoma	SN-38 + Olaparib	0.342	0.034	4.061
905984.000	SF539	Central Nervous System	Glioblastoma	AZD1775 + SN-38	1.000	0.055	5.305
905984.000	SF539	Central Nervous System	Glioblastoma	SRA737 + SN-38	0.971	0.039	5.305

905984.000	SF539	Central Nervous System	Glioblastoma	SN-38 + Olaparib	0.990	0.082	5.305
905984.000	SF539	Central Nervous System	Glioblastoma	Prexasertib + SN-38	1.000	0.030	5.305
905989.000	KM12	Large Intestine	Colorectal Carcinoma	Prexasertib + SN-38	0.928	0.033	0.124
905989.000	KM12	Large Intestine	Colorectal Carcinoma	SN-38 + Olaparib	0.681	0.003	0.124
905990.000	OVCAR-4	Ovary	Ovarian Carcinoma	SN-38 + Olaparib	0.240	-0.007	0.189
905990.000	OVCAR-4	Ovary	Ovarian Carcinoma	Prexasertib + SN-38	0.875	0.054	0.189
906693.000	BxPC-3	Pancreas	Pancreatic Carcinoma	AZD1775 + SN-38	0.797	0.003	2.678
906693.000	BxPC-3	Pancreas	Pancreatic Carcinoma	SRA737 + SN-38	0.671	0.037	2.678
906693.000	BxPC-3	Pancreas	Pancreatic Carcinoma	SN-38 + Olaparib	0.590	0.015	2.678
906693.000	BxPC-3	Pancreas	Pancreatic Carcinoma	Prexasertib + SN-38	0.812	0.042	2.678
906746.000	Becker	Central Nervous System	Low Grade Glioma	AZD1775 + SN-38	0.903	0.026	3.785
906746.000	Becker	Central Nervous System	Low Grade Glioma	SN-38 + Olaparib	0.818	0.094	3.785
906746.000	Becker	Central Nervous System	Low Grade Glioma	Prexasertib + SN-38	0.837	-0.009	3.785
906746.000	Becker	Central Nervous System	Low Grade Glioma	SRA737 + SN-38	0.865	0.024	3.785
906790.000	AGS	Stomach	Gastric Carcinoma	Prexasertib + SN-38	0.860	-0.034	0.138
906790.000	AGS	Stomach	Gastric Carcinoma	SN-38 + Olaparib	0.884	0.105	0.138
906792.000	A2058	Skin	Melanoma	SN-38 + Olaparib	0.219	0.045	0.111
906792.000	A2058	Skin	Melanoma	Prexasertib + SN-38	0.847	0.194	0.111
906793.000	A375	Skin	Melanoma	AZD1775 + SN-38	0.972	0.027	4.037
906793.000	A375	Skin	Melanoma	AZD1775 + SN-38	0.961	0.023	4.037
906793.000	A375	Skin	Melanoma	SN-38 + Olaparib	0.974	0.081	4.037
906793.000	A375	Skin	Melanoma	SN-38 + Olaparib	0.912	0.057	4.037
906793.000	A375	Skin	Melanoma	SRA737 + SN-38	0.931	0.017	4.037
906793.000	A375	Skin	Melanoma	SN-38 + Olaparib	0.990	0.014	4.037
906793.000	A375	Skin	Melanoma	Prexasertib + SN-38	0.989	0.016	4.037
906793.000	A375	Skin	Melanoma	SRA737 + SN-38	0.944	0.011	4.037
906793.000	A375	Skin	Melanoma	AZD1775 + SN-38	0.951	0.053	4.037
906793.000	A375	Skin	Melanoma	AZD1775 + SN-38	0.975	0.025	4.037
906793.000	A375	Skin	Melanoma	Prexasertib + SN-38	0.964	-0.054	4.037
906793.000	A375	Skin	Melanoma	SRA737 + SN-38	0.970	0.022	4.037
906793.000	A375	Skin	Melanoma	Prexasertib + SN-38	0.981	0.012	4.037
906793.000	A375	Skin	Melanoma	Prexasertib + SN-38	0.981	0.031	4.037
906793.000	A375	Skin	Melanoma	SRA737 + SN-38	0.943	0.013	4.037
906793.000	A375	Skin	Melanoma	SN-38 + Olaparib	0.937	0.072	4.037
906793.000	A375	Skin	Melanoma	AZD1775 + SN-38	0.976	0.039	4.037
906793.000	A375	Skin	Melanoma	SRA737 + SN-38	0.949	0.030	4.037
906793.000	A375	Skin	Melanoma	Prexasertib + SN-38	0.982	0.009	4.037
906793.000	A375	Skin	Melanoma	AZD1775 + SN-38	0.994	0.058	4.037
906793.000	A375	Skin	Melanoma	SN-38 + Olaparib	0.963	0.080	4.037
906793.000	A375	Skin	Melanoma	Prexasertib + SN-38	0.989	-0.014	4.037
906793.000	A375	Skin	Melanoma	SRA737 + SN-38	0.922	-0.006	4.037
906793.000	A375	Skin	Melanoma	Prexasertib + SN-38	0.972	0.016	4.037
906793.000	A375	Skin	Melanoma	SN-38 + Olaparib	0.979	0.088	4.037
906793.000	A375	Skin	Melanoma	SRA737 + SN-38	0.886	-0.038	4.037
906793.000	A375	Skin	Melanoma	SN-38 + Olaparib	0.942	0.049	4.037
906793.000	A375	Skin	Melanoma	Prexasertib + SN-38	0.962	-0.019	4.037
906793.000	A375	Skin	Melanoma	SN-38 + Olaparib	0.856	0.039	4.037
906793.000	A375	Skin	Melanoma	AZD1775 + SN-38	0.946	0.045	4.037
906793.000	A375	Skin	Melanoma	SN-38 + Olaparib	0.945	0.082	4.037
906793.000	A375	Skin	Melanoma	SN-38 + Olaparib	0.959	0.068	4.037
906793.000	A375	Skin	Melanoma	SN-38 + Olaparib	0.931	0.097	4.037
906793.000	A375	Skin	Melanoma	Prexasertib + SN-38	0.956	0.005	4.037
906793.000	A375	Skin	Melanoma	Prexasertib + SN-38	0.982	0.013	4.037
906793.000	A375	Skin	Melanoma	AZD1775 + SN-38	0.958	0.042	4.037
906793.000	A375	Skin	Melanoma	Prexasertib + SN-38	0.962	-0.018	4.037
906793.000	A375	Skin	Melanoma	SRA737 + SN-38	0.912	-0.016	4.037
906793.000	A375	Skin	Melanoma	Prexasertib + SN-38	0.958	-0.006	4.037
906793.000	A375	Skin	Melanoma	SRA737 + SN-38	0.925	-0.003	4.037
906793.000	A375	Skin	Melanoma	SRA737 + SN-38	0.893	-0.026	4.037
906793.000	A375	Skin	Melanoma	AZD1775 + SN-38	0.936	0.010	4.037
906793.000	A375	Skin	Melanoma	AZD1775 + SN-38	0.941	0.047	4.037
906793.000	A375	Skin	Melanoma	AZD1775 + SN-38	0.901	0.002	4.037
906793.000	A375	Skin	Melanoma	SN-38 + Olaparib	0.969	0.058	4.037
906793.000	A375	Skin	Melanoma	Prexasertib + SN-38	0.963	-0.006	4.037
906793.000	A375	Skin	Melanoma	SRA737 + SN-38	0.882	-0.003	4.037
906793.000	A375	Skin	Melanoma	AZD1775 + SN-38	0.972	0.032	4.037
906793.000	A375	Skin	Melanoma	SN-38 + Olaparib	0.953	0.099	4.037
906793.000	A375	Skin	Melanoma	AZD1775 + SN-38	0.939	0.025	4.037
906793.000	A375	Skin	Melanoma	SN-38 + Olaparib	0.838	0.075	4.037
906793.000	A375	Skin	Melanoma	SRA737 + SN-38	0.877	-0.015	4.037
906793.000	A375	Skin	Melanoma	SRA737 + SN-38	0.899	-0.033	4.037
906793.000	A375	Skin	Melanoma	SRA737 + SN-38	0.970	0.000	4.037
906793.000	A375	Skin	Melanoma	AZD1775 + SN-38	0.926	0.006	4.037
906793.000	A375	Skin	Melanoma	Prexasertib + SN-38	0.939	-0.017	4.037
906794.000	A253	Head and Neck	Head and Neck Carcinoma	Prexasertib + SN-38	0.967	0.086	3.950
906794.000	A253	Head and Neck	Head and Neck Carcinoma	SN-38 + Olaparib	0.938	0.033	3.950
906795.000	8305C	Thyroid	Thyroid Gland Carcinoma	SN-38 + Olaparib	0.627	0.067	2.590
906795.000	8305C	Thyroid	Thyroid Gland Carcinoma	SRA737 + SN-38	0.615	0.023	2.590
906795.000	8305C	Thyroid	Thyroid Gland Carcinoma	Prexasertib + SN-38	0.803	0.023	2.590
906795.000	8305C	Thyroid	Thyroid Gland Carcinoma	AZD1775 + SN-38	0.679	0.037	2.590
906798.000	639-V	Bladder	Bladder Carcinoma	SN-38 + Olaparib	1.000	0.095	6.229
906798.000	639-V	Bladder	Bladder Carcinoma	Prexasertib + SN-38	1.000	-0.059	6.229
906800.000	697.000	Haematopoietic and Lymphoid	B-Lymphoblastic Leukemia	SN-38 + Olaparib	1.000	0.112	5.910
906800.000	697.000	Haematopoietic and Lymphoid	B-Lymphoblastic Leukemia	Prexasertib + SN-38	1.000	0.022	5.910
906801.000	BT-20	Breast	Breast Carcinoma	SN-38 + Olaparib	0.855	0.119	3.203
906801.000	BT-20	Breast	Breast Carcinoma	Prexasertib + SN-38	0.931	0.113	3.203
906804.000	A2780	Ovary	Ovarian Carcinoma	SN-38 + Olaparib	0.777	0.091	0.057
906804.000	A2780	Ovary	Ovarian Carcinoma	Prexasertib + SN-38	0.843	-0.013	0.057
906804.000	A2780	Ovary	Ovarian Carcinoma	SRA737 + SN-38	0.850	-0.010	0.057
906804.000	A2780	Ovary	Ovarian Carcinoma	AZD1775 + SN-38	0.991	0.027	0.057
906805.000	COR-L105	Lung	Lung Adenocarcinoma	AZD1775 + SN-38	0.563	0.036	0.163
906805.000	COR-L105	Lung	Lung Adenocarcinoma	Prexasertib + SN-38	0.507	-0.016	0.163
906805.000	COR-L105	Lung	Lung Adenocarcinoma	SRA737 + SN-38	0.380	0.001	0.163
906805.000	COR-L105	Lung	Lung Adenocarcinoma	SN-38 + Olaparib	0.401	-0.001	0.163
906808.000	COR-L88	Lung	Small Cell Lung Carcinoma	AZD1775 + SN-38	0.842	0.046	2.331
906808.000	COR-L88	Lung	Small Cell Lung Carcinoma	SRA737 + SN-38	0.645	0.040	2.331
906808.000	COR-L88	Lung	Small Cell Lung Carcinoma	Prexasertib + SN-38	0.860	0.047	2.331
906808.000	COR-L88	Lung	Small Cell Lung Carcinoma	SN-38 + Olaparib	0.535	0.119	2.331
906814.000	COLO-792	Skin	Melanoma	Prexasertib + SN-38	0.554	0.101	0.029
906814.000	COLO-792	Skin	Melanoma	SN-38 + Olaparib	0.484	0.041	0.029
906817.000	COLO-680N	Esophagus	Esophageal Squamous Cell Carcinoma	SRA737 + SN-38	0.552	0.054	5.157
906817.000	COLO-680N	Esophagus	Esophageal Squamous Cell Carcinoma	SN-38 + Olaparib	0.386	0.017	5.157
906817.000	COLO-680N	Esophagus	Esophageal Squamous Cell Carcinoma	AZD1775 + SN-38	0.616	0.055	5.157
906817.000	COLO-680N	Esophagus	Esophageal Squamous Cell Carcinoma	Prexasertib + SN-38	0.733	0.071	5.157
906818.000	COLO-679	Skin	Melanoma	AZD1775 + SN-38	0.651	-0.063	1.618

906818.000	COLO-679	Skin	Melanoma	Prexasertib + SN-38	0.803	-0.026	1.618
906818.000	COLO-679	Skin	Melanoma	SN-38 + Olaparib	0.743	0.021	1.618
906818.000	COLO-679	Skin	Melanoma	SRA737 + SN-38	0.723	-0.021	1.618
906821.000	CFPAC-1	Pancreas	Pancreatic Carcinoma	Prexasertib + SN-38	0.837	-0.043	3.721
906821.000	CFPAC-1	Pancreas	Pancreatic Carcinoma	SN-38 + Olaparib	0.830	0.079	3.721
906824.000	Ca-Ski	Cervix	Cervical Carcinoma	SN-38 + Olaparib	0.997	0.053	2.646
906824.000	Ca-Ski	Cervix	Cervical Carcinoma	SRA737 + SN-38	0.992	0.069	2.646
906824.000	Ca-Ski	Cervix	Cervical Carcinoma	Prexasertib + SN-38	1.000	0.037	2.646
906824.000	Ca-Ski	Cervix	Cervical Carcinoma	AZD1775 + SN-38	1.000	0.115	2.646
906825.000	Caov-3	Ovary	Ovarian Carcinoma	SN-38 + Olaparib	0.989	0.145	5.114
906825.000	Caov-3	Ovary	Ovarian Carcinoma	SN-38 + Olaparib	0.979	0.085	5.114
906825.000	Caov-3	Ovary	Ovarian Carcinoma	Prexasertib + SN-38	0.985	-0.006	5.114
906825.000	Caov-3	Ovary	Ovarian Carcinoma	Prexasertib + SN-38	0.983	0.003	5.114
906826.000	CAL-120	Breast	Breast Carcinoma	SN-38 + Olaparib	0.699	0.052	0.124
906826.000	CAL-120	Breast	Breast Carcinoma	Prexasertib + SN-38	0.770	0.101	0.124
906827.000	CAL-72	Bone	Osteosarcoma	AZD1775 + SN-38	0.902	0.104	4.172
906827.000	CAL-72	Bone	Osteosarcoma	SRA737 + SN-38	0.910	0.082	4.172
906827.000	CAL-72	Bone	Osteosarcoma	Prexasertib + SN-38	0.916	0.031	4.172
906827.000	CAL-72	Bone	Osteosarcoma	SN-38 + Olaparib	0.708	0.133	4.172
906828.000	CAL-62	Thyroid	Thyroid Gland Carcinoma	AZD1775 + SN-38	0.905	0.014	5.598
906828.000	CAL-62	Thyroid	Thyroid Gland Carcinoma	Prexasertib + SN-38	0.884	-0.002	5.598
906828.000	CAL-62	Thyroid	Thyroid Gland Carcinoma	SN-38 + Olaparib	0.906	0.024	5.598
906828.000	CAL-62	Thyroid	Thyroid Gland Carcinoma	SN-38 + Olaparib	0.916	0.024	5.598
906828.000	CAL-62	Thyroid	Thyroid Gland Carcinoma	SRA737 + SN-38	0.865	-0.008	5.598
906828.000	CAL-62	Thyroid	Thyroid Gland Carcinoma	Prexasertib + SN-38	0.869	-0.008	5.598
906830.000	C32	Skin	Melanoma	Prexasertib + SN-38	0.671	-0.066	4.991
906830.000	C32	Skin	Melanoma	SN-38 + Olaparib	0.712	0.070	4.991
906830.000	C32	Skin	Melanoma	SN-38 + Olaparib	0.714	0.052	4.991
906830.000	C32	Skin	Melanoma	AZD1775 + SN-38	0.748	0.056	4.991
906830.000	C32	Skin	Melanoma	SN-38 + Olaparib	0.542	0.187	4.991
906830.000	C32	Skin	Melanoma	Prexasertib + SN-38	0.623	-0.102	4.991
906830.000	C32	Skin	Melanoma	Prexasertib + SN-38	0.785	-0.097	4.991
906830.000	C32	Skin	Melanoma	Prexasertib + SN-38	0.669	-0.020	4.991
906830.000	C32	Skin	Melanoma	SN-38 + Olaparib	0.656	0.132	4.991
906830.000	C32	Skin	Melanoma	AZD1775 + SN-38	0.729	0.023	4.991
906830.000	C32	Skin	Melanoma	Prexasertib + SN-38	0.606	-0.112	4.991
906830.000	C32	Skin	Melanoma	AZD1775 + SN-38	0.715	0.029	4.991
906830.000	C32	Skin	Melanoma	SRA737 + SN-38	0.626	-0.004	4.991
906830.000	C32	Skin	Melanoma	SN-38 + Olaparib	0.678	0.078	4.991
906830.000	C32	Skin	Melanoma	SN-38 + Olaparib	0.587	0.162	4.991
906830.000	C32	Skin	Melanoma	Prexasertib + SN-38	0.715	-0.059	4.991
906830.000	C32	Skin	Melanoma	SRA737 + SN-38	0.650	-0.021	4.991
906830.000	C32	Skin	Melanoma	Prexasertib + SN-38	0.728	-0.086	4.991
906830.000	C32	Skin	Melanoma	Prexasertib + SN-38	0.623	-0.086	4.991
906830.000	C32	Skin	Melanoma	SN-38 + Olaparib	0.673	0.066	4.991
906830.000	C32	Skin	Melanoma	SN-38 + Olaparib	0.559	0.183	4.991
906830.000	C32	Skin	Melanoma	SRA737 + SN-38	0.518	0.001	4.991
906830.000	C32	Skin	Melanoma	Prexasertib + SN-38	0.665	-0.099	4.991
906830.000	C32	Skin	Melanoma	SRA737 + SN-38	0.484	-0.004	4.991
906830.000	C32	Skin	Melanoma	SN-38 + Olaparib	0.653	0.171	4.991
906830.000	C32	Skin	Melanoma	SN-38 + Olaparib	0.677	0.142	4.991
906830.000	C32	Skin	Melanoma	AZD1775 + SN-38	0.622	0.031	4.991
906830.000	C32	Skin	Melanoma	AZD1775 + SN-38	0.585	0.011	4.991
906830.000	C32	Skin	Melanoma	AZD1775 + SN-38	0.708	0.042	4.991
906830.000	C32	Skin	Melanoma	Prexasertib + SN-38	0.730	-0.044	4.991
906830.000	C32	Skin	Melanoma	Prexasertib + SN-38	0.712	-0.108	4.991
906830.000	C32	Skin	Melanoma	SRA737 + SN-38	0.633	0.022	4.991
906830.000	C32	Skin	Melanoma	SN-38 + Olaparib	0.684	0.060	4.991
906830.000	C32	Skin	Melanoma	SN-38 + Olaparib	0.686	0.136	4.991
906830.000	C32	Skin	Melanoma	Prexasertib + SN-38	0.755	-0.073	4.991
906830.000	C32	Skin	Melanoma	SN-38 + Olaparib	0.655	0.202	4.991
906830.000	C32	Skin	Melanoma	SRA737 + SN-38	0.511	0.034	4.991
906830.000	C32	Skin	Melanoma	Prexasertib + SN-38	0.723	-0.053	4.991
906830.000	C32	Skin	Melanoma	AZD1775 + SN-38	0.600	0.033	4.991
906830.000	C32	Skin	Melanoma	Prexasertib + SN-38	0.659	-0.085	4.991
906830.000	C32	Skin	Melanoma	SN-38 + Olaparib	0.650	0.088	4.991
906830.000	C32	Skin	Melanoma	SRA737 + SN-38	0.651	-0.032	4.991
906831.000	Daudi	Haematopoietic and Lymphoid	Burkitt's Lymphoma	SN-38 + Olaparib	0.979	0.131	0.202
906831.000	Daudi	Haematopoietic and Lymphoid	Burkitt's Lymphoma	Prexasertib + SN-38	0.982	0.003	0.202
906832.000	DB	Haematopoietic and Lymphoid	B-Cell Non-Hodgkin's Lymphoma	SN-38 + Olaparib	0.979	0.070	0.151
906832.000	DB	Haematopoietic and Lymphoid	B-Cell Non-Hodgkin's Lymphoma	Prexasertib + SN-38	0.976	0.017	0.151
906832.000	DB	Haematopoietic and Lymphoid	B-Cell Non-Hodgkin's Lymphoma	SRA737 + SN-38	0.988	-0.032	0.151
906832.000	DB	Haematopoietic and Lymphoid	B-Cell Non-Hodgkin's Lymphoma	AZD1775 + SN-38	1.000	0.010	0.151
906832.000	DB	Haematopoietic and Lymphoid	B-Cell Non-Hodgkin's Lymphoma	SN-38 + Olaparib	0.940	0.087	0.151
906832.000	DB	Haematopoietic and Lymphoid	B-Cell Non-Hodgkin's Lymphoma	Prexasertib + SN-38	0.983	0.025	0.151
906833.000	Daoy	Central Nervous System	Other Solid Carcinomas	SN-38 + Olaparib	0.971	0.137	5.755
906833.000	Daoy	Central Nervous System	Other Solid Carcinomas	Prexasertib + SN-38	0.998	0.037	5.755
906834.000	D-283MED	Central Nervous System	Other Solid Carcinomas	SRA737 + SN-38	0.994	0.002	0.151
906834.000	D-283MED	Central Nervous System	Other Solid Carcinomas	SN-38 + Olaparib	0.947	0.043	0.151
906834.000	D-283MED	Central Nervous System	Other Solid Carcinomas	Prexasertib + SN-38	0.981	0.022	0.151
906834.000	D-283MED	Central Nervous System	Other Solid Carcinomas	AZD1775 + SN-38	1.000	0.021	0.151
906835.000	DBTRG-05MG	Central Nervous System	Glioblastoma	Prexasertib + SN-38	0.440	-0.020	3.741
906835.000	DBTRG-05MG	Central Nervous System	Glioblastoma	SN-38 + Olaparib	0.407	0.027	3.741
906836.000	DEL	Haematopoietic and Lymphoid	T-Cell Non-Hodgkin's Lymphoma	SN-38 + Olaparib	1.000	0.108	4.938
906836.000	DEL	Haematopoietic and Lymphoid	T-Cell Non-Hodgkin's Lymphoma	Prexasertib + SN-38	1.000	0.003	4.938
906839.000	DK-MG	Central Nervous System	Glioblastoma	SN-38 + Olaparib	0.259	0.023	4.166
906839.000	DK-MG	Central Nervous System	Glioblastoma	Prexasertib + SN-38	0.245	-0.003	4.166
906843.000	DoTc2-4510	Cervix	Cervical Carcinoma	AZD1775 + SN-38	0.948	0.102	1.967
906843.000	DoTc2-4510	Cervix	Cervical Carcinoma	Prexasertib + SN-38	1.000	0.055	1.967
906843.000	DoTc2-4510	Cervix	Cervical Carcinoma	SN-38 + Olaparib	0.988	0.057	1.967
906843.000	DoTc2-4510	Cervix	Cervical Carcinoma	SRA737 + SN-38	0.936	0.061	1.967
906844.000	DU-4475	Breast	Breast Carcinoma	SN-38 + Olaparib	0.942	-0.006	0.070
906844.000	DU-4475	Breast	Breast Carcinoma	Prexasertib + SN-38	1.000	-0.003	0.070
906848.000	ECC10	Stomach	Gastric Carcinoma	Prexasertib + SN-38	1.000	0.045	0.098
906848.000	ECC10	Stomach	Gastric Carcinoma	SN-38 + Olaparib	1.000	0.133	0.098
906848.000	ECC10	Stomach	Gastric Carcinoma	AZD1775 + SN-38	1.000	-0.004	0.098
906848.000	ECC10	Stomach	Gastric Carcinoma	SRA737 + SN-38	0.972	0.008	0.098
906851.000	EFM-19	Breast	Breast Carcinoma	AZD1775 + SN-38	0.743	0.037	0.057
906851.000	EFM-19	Breast	Breast Carcinoma	SRA737 + SN-38	0.689	0.144	0.057
906851.000	EFM-19	Breast	Breast Carcinoma	Prexasertib + SN-38	0.861	0.146	0.057
906851.000	EFM-19	Breast	Breast Carcinoma	SN-38 + Olaparib	0.566	0.072	0.057
906853.000	EGI-1	Biliary Tract	Biliary Tract Carcinoma	SN-38 + Olaparib	0.444	0.045	0.000
906853.000	EGI-1	Biliary Tract	Biliary Tract Carcinoma	Prexasertib + SN-38	0.849	0.073	0.000
906855.000	EM-2	Haematopoietic and Lymphoid	Chronic Myelogenous Leukemia	SN-38 + Olaparib	1.000	0.069	6.735
906855.000	EM-2	Haematopoietic and Lymphoid	Chronic Myelogenous Leukemia	Prexasertib + SN-38	0.995	-0.022	6.735

906856.000	EoL-1-cell	Haematopoietic and Lymphoid	Acute Myeloid Leukemia	SN-38 + Olaparib	0.990	0.139	5.618
906856.000	EoL-1-cell	Haematopoietic and Lymphoid	Acute Myeloid Leukemia	Prexasertib + SN-38	0.993	-0.032	5.618
906856.000	EoL-1-cell	Haematopoietic and Lymphoid	Acute Myeloid Leukemia	SRA737 + SN-38	0.994	-0.002	5.618
906856.000	EoL-1-cell	Haematopoietic and Lymphoid	Acute Myeloid Leukemia	AZD1775 + SN-38	1.000	0.054	5.618
906863.000	FADU	Head and Neck	Head and Neck Carcinoma	SN-38 + Olaparib	0.996	0.093	6.185
906863.000	FADU	Head and Neck	Head and Neck Carcinoma	Prexasertib + SN-38	0.996	-0.039	6.185
906865.000	G-361	Skin	Melanoma	Prexasertib + SN-38	0.665	-0.002	0.084
906865.000	G-361	Skin	Melanoma	SN-38 + Olaparib	0.459	0.041	0.084
906868.000	GAMG	Central Nervous System	Glioblastoma	Prexasertib + SN-38	1.000	0.066	3.758
906868.000	GAMG	Central Nervous System	Glioblastoma	SN-38 + Olaparib	0.762	0.133	3.758
906868.000	GAMG	Central Nervous System	Glioblastoma	SRA737 + SN-38	0.878	0.110	3.758
906868.000	GAMG	Central Nervous System	Glioblastoma	AZD1775 + SN-38	0.974	0.093	3.758
906869.000	GCIY	Stomach	Gastric Carcinoma	SRA737 + SN-38	0.850	0.027	4.505
906869.000	GCIY	Stomach	Gastric Carcinoma	SN-38 + Olaparib	0.779	0.110	4.505
906869.000	GCIY	Stomach	Gastric Carcinoma	Prexasertib + SN-38	0.916	0.040	4.505
906869.000	GCIY	Stomach	Gastric Carcinoma	AZD1775 + SN-38	0.895	0.048	4.505
906871.000	GI-1	Central Nervous System	Glioblastoma	AZD1775 + SN-38	1.000	0.017	4.282
906871.000	GI-1	Central Nervous System	Glioblastoma	SRA737 + SN-38	0.973	0.058	4.282
906871.000	GI-1	Central Nervous System	Glioblastoma	SN-38 + Olaparib	0.954	0.069	4.282
906871.000	GI-1	Central Nervous System	Glioblastoma	Prexasertib + SN-38	1.000	0.035	4.282
906872.000	GI-ME-N	Peripheral Nervous System	Neuroblastoma	Prexasertib + SN-38	0.564	0.002	0.043
906872.000	GI-ME-N	Peripheral Nervous System	Neuroblastoma	SN-38 + Olaparib	0.494	0.080	0.043
906999.000	GCT	Soft Tissue	Other Solid Carcinomas	Prexasertib + SN-38	0.850	0.046	3.888
906999.000	GCT	Soft Tissue	Other Solid Carcinomas	SN-38 + Olaparib	0.753	0.115	3.888
907000.000	ESS-1	Endometrium	Other Solid Carcinomas	Prexasertib + SN-38	1.000	0.020	6.047
907000.000	ESS-1	Endometrium	Other Solid Carcinomas	SRA737 + SN-38	1.000	0.022	6.047
907000.000	ESS-1	Endometrium	Other Solid Carcinomas	AZD1775 + SN-38	1.000	0.027	6.047
907000.000	ESS-1	Endometrium	Other Solid Carcinomas	SN-38 + Olaparib	1.000	0.088	6.047
907042.000	H4	Central Nervous System	Low Grade Glioma	AZD1775 + SN-38	0.944	0.057	5.139
907042.000	H4	Central Nervous System	Low Grade Glioma	Prexasertib + SN-38	0.949	-0.009	5.139
907042.000	H4	Central Nervous System	Low Grade Glioma	SRA737 + SN-38	0.832	0.032	5.139
907042.000	H4	Central Nervous System	Low Grade Glioma	SN-38 + Olaparib	0.938	0.139	5.139
907047.000	HCC1806	Breast	Breast Carcinoma	SN-38 + Olaparib	0.974	0.057	3.715
907047.000	HCC1806	Breast	Breast Carcinoma	Prexasertib + SN-38	0.993	0.011	3.715
907048.000	HCC70	Breast	Breast Carcinoma	SN-38 + Olaparib	0.455	0.057	0.239
907048.000	HCC70	Breast	Breast Carcinoma	SRA737 + SN-38	0.674	0.028	0.239
907048.000	HCC70	Breast	Breast Carcinoma	Prexasertib + SN-38	0.970	-0.010	0.239
907048.000	HCC70	Breast	Breast Carcinoma	AZD1775 + SN-38	0.779	-0.021	0.239
907050.000	HD-MY-Z	Haematopoietic and Lymphoid	Hodgkin's Lymphoma	Prexasertib + SN-38	0.692	0.016	5.454
907050.000	HD-MY-Z	Haematopoietic and Lymphoid	Hodgkin's Lymphoma	SN-38 + Olaparib	0.533	0.070	5.454
907055.000	HGC-27	Stomach	Gastric Carcinoma	Prexasertib + SN-38	0.988	0.068	0.084
907055.000	HGC-27	Stomach	Gastric Carcinoma	SN-38 + Olaparib	0.852	-0.022	0.084
907056.000	HH	Haematopoietic and Lymphoid	T-Cell Non-Hodgkin's Lymphoma	AZD1775 + SN-38	0.697	-0.038	1.820
907056.000	HH	Haematopoietic and Lymphoid	T-Cell Non-Hodgkin's Lymphoma	SRA737 + SN-38	0.452	0.023	1.820
907056.000	HH	Haematopoietic and Lymphoid	T-Cell Non-Hodgkin's Lymphoma	SN-38 + Olaparib	0.103	-0.021	1.820
907056.000	HH	Haematopoietic and Lymphoid	T-Cell Non-Hodgkin's Lymphoma	Prexasertib + SN-38	0.821	0.055	1.820
907060.000	HOS	Bone	Osteosarcoma	SRA737 + SN-38	0.849	0.054	4.760
907060.000	HOS	Bone	Osteosarcoma	AZD1775 + SN-38	0.872	0.000	4.760
907060.000	HOS	Bone	Osteosarcoma	SN-38 + Olaparib	0.960	0.093	4.760
907060.000	HOS	Bone	Osteosarcoma	Prexasertib + SN-38	0.989	0.073	4.760
907061.000	HSC-3	Head and Neck	Oral Cavity Carcinoma	SN-38 + Olaparib	0.847	0.033	4.309
907061.000	HSC-3	Head and Neck	Oral Cavity Carcinoma	Prexasertib + SN-38	0.884	-0.040	4.309
907064.000	HT-1080	Soft Tissue	Other Solid Carcinomas	Prexasertib + SN-38	0.526	0.001	2.401
907064.000	HT-1080	Soft Tissue	Other Solid Carcinomas	SN-38 + Olaparib	0.489	0.105	2.401
907065.000	HT-1197	Bladder	Bladder Carcinoma	SN-38 + Olaparib	0.350	0.055	0.029
907065.000	HT-1197	Bladder	Bladder Carcinoma	Prexasertib + SN-38	0.563	0.014	0.029
907066.000	HT-1376	Bladder	Bladder Carcinoma	SN-38 + Olaparib	0.083	-0.032	0.807
907066.000	HT-1376	Bladder	Bladder Carcinoma	AZD1775 + SN-38	0.775	0.015	0.807
907066.000	HT-1376	Bladder	Bladder Carcinoma	Prexasertib + SN-38	0.553	-0.023	0.807
907066.000	HT-1376	Bladder	Bladder Carcinoma	SRA737 + SN-38	0.566	0.023	0.807
907069.000	HuCCT1	Biliary Tract	Biliary Tract Carcinoma	SRA737 + SN-38	0.847	0.098	0.057
907069.000	HuCCT1	Biliary Tract	Biliary Tract Carcinoma	AZD1775 + SN-38	0.896	0.048	0.057
907069.000	HuCCT1	Biliary Tract	Biliary Tract Carcinoma	SN-38 + Olaparib	0.662	0.012	0.057
907069.000	HuCCT1	Biliary Tract	Biliary Tract Carcinoma	Prexasertib + SN-38	0.905	0.032	0.057
907072.000	HuO9	Bone	Osteosarcoma	Prexasertib + SN-38	0.995	-0.009	4.383
907072.000	HuO9	Bone	Osteosarcoma	SN-38 + Olaparib	0.996	0.077	4.383
907169.000	IGR-1	Skin	Melanoma	SN-38 + Olaparib	0.634	0.041	0.057
907169.000	IGR-1	Skin	Melanoma	Prexasertib + SN-38	0.815	0.112	0.057
907173.000	IST-MES1	Lung	Mesothelioma	Prexasertib + SN-38	0.848	-0.004	5.738
907173.000	IST-MES1	Lung	Mesothelioma	SN-38 + Olaparib	0.630	-0.030	5.738
907173.000	IST-MES1	Lung	Mesothelioma	SRA737 + SN-38	0.759	0.025	5.738
907173.000	IST-MES1	Lung	Mesothelioma	AZD1775 + SN-38	0.778	0.110	5.738
907269.000	JVM-2	Haematopoietic and Lymphoid	B-Cell Non-Hodgkin's Lymphoma	SN-38 + Olaparib	0.935	0.158	3.248
907269.000	JVM-2	Haematopoietic and Lymphoid	B-Cell Non-Hodgkin's Lymphoma	Prexasertib + SN-38	0.970	-0.023	3.248
907270.000	JVM-3	Haematopoietic and Lymphoid	B-Cell Non-Hodgkin's Lymphoma	SRA737 + SN-38	0.960	-0.040	4.957
907270.000	JVM-3	Haematopoietic and Lymphoid	B-Cell Non-Hodgkin's Lymphoma	SN-38 + Olaparib	0.995	0.054	4.957
907270.000	JVM-3	Haematopoietic and Lymphoid	B-Cell Non-Hodgkin's Lymphoma	Prexasertib + SN-38	0.990	-0.001	4.957
907270.000	JVM-3	Haematopoietic and Lymphoid	B-Cell Non-Hodgkin's Lymphoma	AZD1775 + SN-38	0.979	0.000	4.957
907270.000	JVM-3	Haematopoietic and Lymphoid	B-Cell Non-Hodgkin's Lymphoma	Prexasertib + SN-38	0.998	0.011	4.957
907270.000	JVM-3	Haematopoietic and Lymphoid	B-Cell Non-Hodgkin's Lymphoma	SN-38 + Olaparib	1.000	0.049	4.957
907271.000	KALS-1	Central Nervous System	Glioblastoma	AZD1775 + SN-38	0.983	0.138	1.526
907271.000	KALS-1	Central Nervous System	Glioblastoma	SRA737 + SN-38	0.929	0.161	1.526
907271.000	KALS-1	Central Nervous System	Glioblastoma	Prexasertib + SN-38	0.927	0.023	1.526
907271.000	KALS-1	Central Nervous System	Glioblastoma	SN-38 + Olaparib	0.616	0.080	1.526
907273.000	KARPAS-299	Haematopoietic and Lymphoid	T-Cell Non-Hodgkin's Lymphoma	SRA737 + SN-38	0.999	0.053	0.070
907273.000	KARPAS-299	Haematopoietic and Lymphoid	T-Cell Non-Hodgkin's Lymphoma	AZD1775 + SN-38	1.000	0.006	0.070
907273.000	KARPAS-299	Haematopoietic and Lymphoid	T-Cell Non-Hodgkin's Lymphoma	Prexasertib + SN-38	0.990	0.006	0.070
907273.000	KARPAS-299	Haematopoietic and Lymphoid	T-Cell Non-Hodgkin's Lymphoma	SN-38 + Olaparib	0.896	0.049	0.070
907274.000	KARPAS-422	Haematopoietic and Lymphoid	B-Cell Non-Hodgkin's Lymphoma	AZD1775 + SN-38	0.961	0.024	0.189
907274.000	KARPAS-422	Haematopoietic and Lymphoid	B-Cell Non-Hodgkin's Lymphoma	SRA737 + SN-38	0.910	0.061	0.189
907274.000	KARPAS-422	Haematopoietic and Lymphoid	B-Cell Non-Hodgkin's Lymphoma	SN-38 + Olaparib	0.957	0.043	0.189
907274.000	KARPAS-422	Haematopoietic and Lymphoid	B-Cell Non-Hodgkin's Lymphoma	Prexasertib + SN-38	1.000	0.079	0.189
907276.000	KATOIII	Stomach	Gastric Carcinoma	Prexasertib + SN-38	0.582	-0.008	0.356
907276.000	KATOIII	Stomach	Gastric Carcinoma	SRA737 + SN-38	0.543	0.066	0.356
907276.000	KATOIII	Stomach	Gastric Carcinoma	AZD1775 + SN-38	0.516	-0.013	0.356
907276.000	KATOIII	Stomach	Gastric Carcinoma	SN-38 + Olaparib	0.251	-0.031	0.356
907277.000	KE-37	Haematopoietic and Lymphoid	T-Lymphoblastic Leukemia	Prexasertib + SN-38	1.000	-0.002	6.257
907277.000	KE-37	Haematopoietic and Lymphoid	T-Lymphoblastic Leukemia	SN-38 + Olaparib	1.000	0.046	6.257
907285.000	HuP-T3	Pancreas	Pancreatic Carcinoma	SN-38 + Olaparib	0.683	-0.025	0.705
907285.000	HuP-T3	Pancreas	Pancreatic Carcinoma	Prexasertib + SN-38	0.834	0.105	0.705
907286.000	HuP-T4	Pancreas	Pancreatic Carcinoma	SN-38 + Olaparib	0.948	0.102	4.217
907286.000	HuP-T4	Pancreas	Pancreatic Carcinoma	Prexasertib + SN-38	0.948	-0.005	4.217
907287.000	HT55	Large Intestine	Colorectal Carcinoma	SN-38 + Olaparib	0.489	0.113	0.111
907287.000	HT55	Large Intestine	Colorectal Carcinoma	Prexasertib + SN-38	0.762	0.087	0.111
907291.000	GP5d	Large Intestine	Colorectal Carcinoma	SN-38 + Olaparib	0.704	0.156	0.014

907291.000	GP5d	Large Intestine	Colorectal Carcinoma	Prexasertib + SN-38	0.970	0.032	0.014
907295.000	DMS-53	Lung	Small Cell Lung Carcinoma	Prexasertib + SN-38	0.771	0.000	4.716
907295.000	DMS-53	Lung	Small Cell Lung Carcinoma	SN-38 + Olaparib	0.735	0.065	4.716
907298.000	G-402	Soft Tissue	Other Solid Carcinomas	SN-38 + Olaparib	0.828	0.113	0.098
907298.000	G-402	Soft Tissue	Other Solid Carcinomas	SN-38 + Olaparib	0.830	0.099	0.098
907298.000	G-402	Soft Tissue	Other Solid Carcinomas	Prexasertib + SN-38	0.805	-0.021	0.098
907298.000	G-402	Soft Tissue	Other Solid Carcinomas	Prexasertib + SN-38	0.869	0.040	0.098
907311.000	KU812	Haematopoietic and Lymphoid	Chronic Myelogenous Leukemia	Prexasertib + SN-38	0.809	-0.102	5.936
907311.000	KU812	Haematopoietic and Lymphoid	Chronic Myelogenous Leukemia	SN-38 + Olaparib	0.756	0.033	5.936
907311.000	KU812	Haematopoietic and Lymphoid	Chronic Myelogenous Leukemia	AZD1775 + SN-38	0.989	0.042	5.936
907311.000	KU812	Haematopoietic and Lymphoid	Chronic Myelogenous Leukemia	SRA737 + SN-38	0.799	-0.047	5.936
907313.000	KS-1	Central Nervous System	Glioblastoma	Prexasertib + SN-38	0.503	-0.028	0.202
907313.000	KS-1	Central Nervous System	Glioblastoma	SRA737 + SN-38	0.458	-0.058	0.202
907313.000	KS-1	Central Nervous System	Glioblastoma	AZD1775 + SN-38	0.571	-0.001	0.202
907313.000	KS-1	Central Nervous System	Glioblastoma	SN-38 + Olaparib	0.556	0.026	0.202
907314.000	KP-N-YN	Peripheral Nervous System	Neuroblastoma	SN-38 + Olaparib	0.491	0.026	0.098
907314.000	KP-N-YN	Peripheral Nervous System	Neuroblastoma	AZD1775 + SN-38	0.936	0.052	0.098
907314.000	KP-N-YN	Peripheral Nervous System	Neuroblastoma	SRA737 + SN-38	0.928	0.083	0.098
907314.000	KP-N-YN	Peripheral Nervous System	Neuroblastoma	Prexasertib + SN-38	0.989	0.037	0.098
907318.000	KYSE-180	Esophagus	Esophageal Squamous Cell Carcinoma	Prexasertib + SN-38	1.000	0.069	0.043
907318.000	KYSE-180	Esophagus	Esophageal Squamous Cell Carcinoma	SN-38 + Olaparib	0.527	0.049	0.043
907319.000	KYSE-270	Esophagus	Esophageal Squamous Cell Carcinoma	AZD1775 + SN-38	1.000	0.076	3.751
907319.000	KYSE-270	Esophagus	Esophageal Squamous Cell Carcinoma	SRA737 + SN-38	1.000	0.037	3.751
907319.000	KYSE-270	Esophagus	Esophageal Squamous Cell Carcinoma	SN-38 + Olaparib	0.949	0.032	3.751
907319.000	KYSE-270	Esophagus	Esophageal Squamous Cell Carcinoma	Prexasertib + SN-38	1.000	0.044	3.751
907320.000	KYSE-450	Esophagus	Esophageal Squamous Cell Carcinoma	SN-38 + Olaparib	0.671	0.102	0.070
907320.000	KYSE-450	Esophagus	Esophageal Squamous Cell Carcinoma	SRA737 + SN-38	0.940	0.191	0.070
907320.000	KYSE-450	Esophagus	Esophageal Squamous Cell Carcinoma	Prexasertib + SN-38	0.999	0.198	0.070
907320.000	KYSE-450	Esophagus	Esophageal Squamous Cell Carcinoma	AZD1775 + SN-38	0.973	0.101	0.070
907322.000	L-428	Haematopoietic and Lymphoid	Hodgkin's Lymphoma	Prexasertib + SN-38	0.958	-0.024	4.616
907322.000	L-428	Haematopoietic and Lymphoid	Hodgkin's Lymphoma	SN-38 + Olaparib	0.940	0.040	4.616
907323.000	L-540	Haematopoietic and Lymphoid	Hodgkin's Lymphoma	SN-38 + Olaparib	0.965	0.035	5.833
907323.000	L-540	Haematopoietic and Lymphoid	Hodgkin's Lymphoma	Prexasertib + SN-38	0.946	-0.040	5.833
907323.000	L-540	Haematopoietic and Lymphoid	Hodgkin's Lymphoma	SRA737 + SN-38	0.938	-0.003	5.833
907323.000	L-540	Haematopoietic and Lymphoid	Hodgkin's Lymphoma	AZD1775 + SN-38	0.956	-0.021	5.833
907783.000	LAMA-84	Haematopoietic and Lymphoid	Chronic Myelogenous Leukemia	AZD1775 + SN-38	0.995	0.023	5.543
907783.000	LAMA-84	Haematopoietic and Lymphoid	Chronic Myelogenous Leukemia	Prexasertib + SN-38	0.994	-0.010	5.543
907783.000	LAMA-84	Haematopoietic and Lymphoid	Chronic Myelogenous Leukemia	SN-38 + Olaparib	0.948	0.088	5.543
907783.000	LAMA-84	Haematopoietic and Lymphoid	Chronic Myelogenous Leukemia	SRA737 + SN-38	0.953	0.014	5.543
907789.000	LOUCY	Haematopoietic and Lymphoid	T-Lymphoblastic Leukemia	SRA737 + SN-38	0.952	0.139	6.370
907789.000	LOUCY	Haematopoietic and Lymphoid	T-Lymphoblastic Leukemia	AZD1775 + SN-38	1.000	0.058	6.370
907789.000	LOUCY	Haematopoietic and Lymphoid	T-Lymphoblastic Leukemia	SN-38 + Olaparib	0.764	0.031	6.370
907789.000	LOUCY	Haematopoietic and Lymphoid	T-Lymphoblastic Leukemia	Prexasertib + SN-38	1.000	0.028	6.370
907790.000	LoVo	Large Intestine	Colorectal Carcinoma	Prexasertib + SN-38	0.859	-0.047	0.111
907790.000	LoVo	Large Intestine	Colorectal Carcinoma	SN-38 + Olaparib	0.785	0.104	0.111
907790.000	LoVo	Large Intestine	Colorectal Carcinoma	AZD1775 + SN-38	0.889	0.055	0.111
907790.000	LoVo	Large Intestine	Colorectal Carcinoma	SN-38 + Olaparib	0.717	0.068	0.111
907790.000	LoVo	Large Intestine	Colorectal Carcinoma	Prexasertib + SN-38	0.908	-0.030	0.111
907790.000	LoVo	Large Intestine	Colorectal Carcinoma	Prexasertib + SN-38	0.852	-0.038	0.111
907790.000	LoVo	Large Intestine	Colorectal Carcinoma	SN-38 + Olaparib	0.712	0.047	0.111
907790.000	LoVo	Large Intestine	Colorectal Carcinoma	SRA737 + SN-38	0.775	0.058	0.111
907794.000	LS-411N	Large Intestine	Colorectal Carcinoma	SN-38 + Olaparib	0.304	-0.008	0.124
907794.000	LS-411N	Large Intestine	Colorectal Carcinoma	Prexasertib + SN-38	0.838	0.042	0.124
907795.000	LS-513	Large Intestine	Colorectal Carcinoma	SN-38 + Olaparib	0.665	0.098	0.043
907795.000	LS-513	Large Intestine	Colorectal Carcinoma	AZD1775 + SN-38	0.916	0.019	0.043
907795.000	LS-513	Large Intestine	Colorectal Carcinoma	SRA737 + SN-38	0.742	-0.037	0.043
907795.000	LS-513	Large Intestine	Colorectal Carcinoma	Prexasertib + SN-38	0.757	-0.018	0.043
908120.000	MDA-MB-175-VII	Breast	Breast Carcinoma	SRA737 + SN-38	0.630	0.010	4.150
908120.000	MDA-MB-175-VII	Breast	Breast Carcinoma	Prexasertib + SN-38	0.534	0.010	4.150
908120.000	MDA-MB-175-VII	Breast	Breast Carcinoma	SN-38 + Olaparib	0.649	0.052	4.150
908120.000	MDA-MB-175-VII	Breast	Breast Carcinoma	AZD1775 + SN-38	0.659	0.055	4.150
908122.000	MDA-MB-453	Breast	Breast Carcinoma	AZD1775 + SN-38	0.747	0.058	0.299
908122.000	MDA-MB-453	Breast	Breast Carcinoma	Prexasertib + SN-38	0.948	0.067	0.299
908122.000	MDA-MB-453	Breast	Breast Carcinoma	SN-38 + Olaparib	0.746	0.058	0.299
908122.000	MDA-MB-453	Breast	Breast Carcinoma	SRA737 + SN-38	0.719	0.009	0.299
908123.000	MDA-MB-468	Breast	Breast Carcinoma	SRA737 + SN-38	0.791	0.057	4.859
908123.000	MDA-MB-468	Breast	Breast Carcinoma	AZD1775 + SN-38	0.873	0.036	4.859
908123.000	MDA-MB-468	Breast	Breast Carcinoma	Prexasertib + SN-38	0.825	0.068	4.859
908123.000	MDA-MB-468	Breast	Breast Carcinoma	SN-38 + Olaparib	0.773	0.128	4.859
908126.000	MEG-01	Haematopoietic and Lymphoid	Chronic Myelogenous Leukemia	SN-38 + Olaparib	0.768	0.011	0.214
908126.000	MEG-01	Haematopoietic and Lymphoid	Chronic Myelogenous Leukemia	AZD1775 + SN-38	0.996	0.058	0.214
908126.000	MEG-01	Haematopoietic and Lymphoid	Chronic Myelogenous Leukemia	Prexasertib + SN-38	0.939	0.011	0.214
908126.000	MEG-01	Haematopoietic and Lymphoid	Chronic Myelogenous Leukemia	SRA737 + SN-38	0.946	0.116	0.214
908126.000	MEG-01	Haematopoietic and Lymphoid	Chronic Myelogenous Leukemia	SN-38 + Olaparib	0.742	0.061	0.214
908126.000	MEG-01	Haematopoietic and Lymphoid	Chronic Myelogenous Leukemia	Prexasertib + SN-38	0.940	0.029	0.214
908128.000	Mewo	Skin	Melanoma	SRA737 + SN-38	0.679	0.080	0.880
908128.000	Mewo	Skin	Melanoma	SN-38 + Olaparib	0.337	0.029	0.880
908128.000	Mewo	Skin	Melanoma	Prexasertib + SN-38	0.712	0.086	0.880
908128.000	Mewo	Skin	Melanoma	AZD1775 + SN-38	0.698	0.037	0.880
908129.000	MFE-280	Endometrium	Endometrial Carcinoma	AZD1775 + SN-38	0.937	0.088	0.124
908129.000	MFE-280	Endometrium	Endometrial Carcinoma	SRA737 + SN-38	0.880	0.096	0.124
908129.000	MFE-280	Endometrium	Endometrial Carcinoma	SN-38 + Olaparib	0.785	0.042	0.124
908129.000	MFE-280	Endometrium	Endometrial Carcinoma	Prexasertib + SN-38	0.913	0.180	0.124
908130.000	MFE-296	Endometrium	Endometrial Carcinoma	Prexasertib + SN-38	0.720	0.054	5.533
908130.000	MFE-296	Endometrium	Endometrial Carcinoma	SN-38 + Olaparib	0.595	0.086	5.533
908131.000	MG-63	Bone	Osteosarcoma	SN-38 + Olaparib	0.896	0.083	4.977
908131.000	MG-63	Bone	Osteosarcoma	Prexasertib + SN-38	0.960	0.002	4.977
908131.000	MG-63	Bone	Osteosarcoma	SRA737 + SN-38	0.953	0.016	4.977
908131.000	MG-63	Bone	Osteosarcoma	AZD1775 + SN-38	0.968	0.044	4.977
908134.000	MHH-ES-1	Bone	Ewing's Sarcoma	SN-38 + Olaparib	0.965	0.080	5.684
908134.000	MHH-ES-1	Bone	Ewing's Sarcoma	SN-38 + Olaparib	0.978	0.092	5.684
908134.000	MHH-ES-1	Bone	Ewing's Sarcoma	Prexasertib + SN-38	1.000	0.037	5.684
908134.000	MHH-ES-1	Bone	Ewing's Sarcoma	Prexasertib + SN-38	1.000	0.033	5.684
908134.000	MHH-ES-1	Bone	Ewing's Sarcoma	SN-38 + Olaparib	0.977	0.070	5.684
908134.000	MHH-ES-1	Bone	Ewing's Sarcoma	Prexasertib + SN-38	1.000	0.054	5.684
908134.000	MHH-ES-1	Bone	Ewing's Sarcoma	SN-38 + Olaparib	0.953	0.107	5.684
908134.000	MHH-ES-1	Bone	Ewing's Sarcoma	Prexasertib + SN-38	1.000	0.038	5.684
908134.000	MHH-ES-1	Bone	Ewing's Sarcoma	SN-38 + Olaparib	0.972	0.100	5.684
908134.000	MHH-ES-1	Bone	Ewing's Sarcoma	SN-38 + Olaparib	0.972	0.181	5.684
908134.000	MHH-ES-1	Bone	Ewing's Sarcoma	AZD1775 + SN-38	0.975	0.041	5.684
908134.000	MHH-ES-1	Bone	Ewing's Sarcoma	Prexasertib + SN-38	1.000	0.040	5.684
908134.000	MHH-ES-1	Bone	Ewing's Sarcoma	AZD1775 + SN-38	0.995	0.033	5.684
908134.000	MHH-ES-1	Bone	Ewing's Sarcoma	Prexasertib + SN-38	1.000	0.046	5.684
908134.000	MHH-ES-1	Bone	Ewing's Sarcoma	SRA737 + SN-38	0.960	-0.004	5.684
908134.000	MHH-ES-1	Bone	Ewing's Sarcoma	Prexasertib + SN-38	1.000	0.066	5.684

908134.000	MHH-ES-1	Bone	Ewing's Sarcoma	Prexasertib + SN-38	1.000	0.043	5.684
908134.000	MHH-ES-1	Bone	Ewing's Sarcoma	SN-38 + Olaparib	0.994	0.112	5.684
908134.000	MHH-ES-1	Bone	Ewing's Sarcoma	SN-38 + Olaparib	0.985	0.087	5.684
908134.000	MHH-ES-1	Bone	Ewing's Sarcoma	AZD1775 + SN-38	0.981	0.031	5.684
908134.000	MHH-ES-1	Bone	Ewing's Sarcoma	SRA737 + SN-38	0.962	-0.018	5.684
908134.000	MHH-ES-1	Bone	Ewing's Sarcoma	Prexasertib + SN-38	1.000	0.068	5.684
908134.000	MHH-ES-1	Bone	Ewing's Sarcoma	SRA737 + SN-38	0.986	0.010	5.684
908134.000	MHH-ES-1	Bone	Ewing's Sarcoma	SRA737 + SN-38	0.958	-0.044	5.684
908134.000	MHH-ES-1	Bone	Ewing's Sarcoma	SRA737 + SN-38	0.967	-0.037	5.684
908134.000	MHH-ES-1	Bone	Ewing's Sarcoma	SN-38 + Olaparib	0.989	0.058	5.684
908134.000	MHH-ES-1	Bone	Ewing's Sarcoma	Prexasertib + SN-38	1.000	0.060	5.684
908134.000	MHH-ES-1	Bone	Ewing's Sarcoma	SRA737 + SN-38	0.991	0.000	5.684
908134.000	MHH-ES-1	Bone	Ewing's Sarcoma	AZD1775 + SN-38	0.978	0.050	5.684
908134.000	MHH-ES-1	Bone	Ewing's Sarcoma	AZD1775 + SN-38	0.988	0.038	5.684
908134.000	MHH-ES-1	Bone	Ewing's Sarcoma	AZD1775 + SN-38	1.000	0.038	5.684
908134.000	MHH-ES-1	Bone	Ewing's Sarcoma	SRA737 + SN-38	0.987	-0.045	5.684
908134.000	MHH-ES-1	Bone	Ewing's Sarcoma	SN-38 + Olaparib	0.970	0.110	5.684
908134.000	MHH-ES-1	Bone	Ewing's Sarcoma	AZD1775 + SN-38	1.000	0.025	5.684
908134.000	MHH-ES-1	Bone	Ewing's Sarcoma	AZD1775 + SN-38	1.000	0.049	5.684
908134.000	MHH-ES-1	Bone	Ewing's Sarcoma	SRA737 + SN-38	0.975	-0.033	5.684
908134.000	MHH-ES-1	Bone	Ewing's Sarcoma	SRA737 + SN-38	0.990	0.018	5.684
908134.000	MHH-ES-1	Bone	Ewing's Sarcoma	SN-38 + Olaparib	0.989	0.087	5.684
908134.000	MHH-ES-1	Bone	Ewing's Sarcoma	Prexasertib + SN-38	0.996	0.052	5.684
908134.000	MHH-ES-1	Bone	Ewing's Sarcoma	SN-38 + Olaparib	0.980	0.098	5.684
908134.000	MHH-ES-1	Bone	Ewing's Sarcoma	Prexasertib + SN-38	0.997	0.069	5.684
908134.000	MHH-ES-1	Bone	Ewing's Sarcoma	Prexasertib + SN-38	1.000	0.039	5.684
908134.000	MHH-ES-1	Bone	Ewing's Sarcoma	SN-38 + Olaparib	0.982	0.101	5.684
908134.000	MHH-ES-1	Bone	Ewing's Sarcoma	AZD1775 + SN-38	0.997	0.040	5.684
908134.000	MHH-ES-1	Bone	Ewing's Sarcoma	Prexasertib + SN-38	1.000	0.060	5.684
908134.000	MHH-ES-1	Bone	Ewing's Sarcoma	AZD1775 + SN-38	0.979	0.053	5.684
908134.000	MHH-ES-1	Bone	Ewing's Sarcoma	SRA737 + SN-38	0.998	0.002	5.684
908134.000	MHH-ES-1	Bone	Ewing's Sarcoma	SN-38 + Olaparib	0.964	0.120	5.684
908134.000	MHH-ES-1	Bone	Ewing's Sarcoma	SRA737 + SN-38	0.968	0.016	5.684
908134.000	MHH-ES-1	Bone	Ewing's Sarcoma	SN-38 + Olaparib	0.982	0.066	5.684
908134.000	MHH-ES-1	Bone	Ewing's Sarcoma	Prexasertib + SN-38	0.992	0.059	5.684
908134.000	MHH-ES-1	Bone	Ewing's Sarcoma	AZD1775 + SN-38	0.987	0.025	5.684
908138.000	MKN1	Stomach	Gastric Carcinoma	SRA737 + SN-38	0.866	0.091	3.489
908138.000	MKN1	Stomach	Gastric Carcinoma	AZD1775 + SN-38	0.969	0.069	3.489
908146.000	MOLT-13	Haematopoietic and Lymphoid	T-Lymphoblastic Leukemia	Prexasertib + SN-38	1.000	-0.022	6.545
908146.000	MOLT-13	Haematopoietic and Lymphoid	T-Lymphoblastic Leukemia	SN-38 + Olaparib	1.000	0.087	6.545
908148.000	MONO-MAC-6	Haematopoietic and Lymphoid	Acute Myeloid Leukemia	AZD1775 + SN-38	1.000	0.021	6.165
908148.000	MONO-MAC-6	Haematopoietic and Lymphoid	Acute Myeloid Leukemia	SN-38 + Olaparib	1.000	0.149	6.165
908148.000	MONO-MAC-6	Haematopoietic and Lymphoid	Acute Myeloid Leukemia	Prexasertib + SN-38	1.000	0.058	6.165
908148.000	MONO-MAC-6	Haematopoietic and Lymphoid	Acute Myeloid Leukemia	SRA737 + SN-38	1.000	-0.009	6.165
908150.000	MPP-89	Lung	Mesothelioma	SN-38 + Olaparib	0.646	0.064	4.147
908150.000	MPP-89	Lung	Mesothelioma	Prexasertib + SN-38	0.884	0.053	4.147
908150.000	MPP-89	Lung	Mesothelioma	AZD1775 + SN-38	0.769	0.102	4.147
908150.000	MPP-89	Lung	Mesothelioma	SRA737 + SN-38	0.730	0.072	4.147
908152.000	MSTO-211H	Lung	Mesothelioma	SRA737 + SN-38	0.705	0.020	6.086
908152.000	MSTO-211H	Lung	Mesothelioma	AZD1775 + SN-38	0.817	-0.014	6.086
908152.000	MSTO-211H	Lung	Mesothelioma	Prexasertib + SN-38	0.831	-0.036	6.086
908152.000	MSTO-211H	Lung	Mesothelioma	SN-38 + Olaparib	0.677	0.051	6.086
908156.000	MV-4-11	Haematopoietic and Lymphoid	Acute Myeloid Leukemia	SN-38 + Olaparib	1.000	0.070	2.774
908156.000	MV-4-11	Haematopoietic and Lymphoid	Acute Myeloid Leukemia	AZD1775 + SN-38	0.999	-0.003	2.774
908156.000	MV-4-11	Haematopoietic and Lymphoid	Acute Myeloid Leukemia	Prexasertib + SN-38	1.000	0.017	2.774
908156.000	MV-4-11	Haematopoietic and Lymphoid	Acute Myeloid Leukemia	SRA737 + SN-38	0.996	-0.005	2.774
908158.000	NALM-6	Haematopoietic and Lymphoid	B-Lymphoblastic Leukemia	AZD1775 + SN-38	1.000	-0.004	6.591
908158.000	NALM-6	Haematopoietic and Lymphoid	B-Lymphoblastic Leukemia	SRA737 + SN-38	1.000	0.032	6.591
908158.000	NALM-6	Haematopoietic and Lymphoid	B-Lymphoblastic Leukemia	Prexasertib + SN-38	0.989	-0.018	6.591
908158.000	NALM-6	Haematopoietic and Lymphoid	B-Lymphoblastic Leukemia	SN-38 + Olaparib	0.990	0.095	6.591
908159.000	NAMALWA	Haematopoietic and Lymphoid	Burkitt's Lymphoma	Prexasertib + SN-38	0.984	0.004	4.654
908159.000	NAMALWA	Haematopoietic and Lymphoid	Burkitt's Lymphoma	SN-38 + Olaparib	0.986	0.075	4.654
908159.000	NAMALWA	Haematopoietic and Lymphoid	Burkitt's Lymphoma	SRA737 + SN-38	0.994	0.012	4.654
908159.000	NAMALWA	Haematopoietic and Lymphoid	Burkitt's Lymphoma	AZD1775 + SN-38	1.000	0.004	4.654
908443.000	NCI-H520	Lung	Squamous Cell Lung Carcinoma	AZD1775 + SN-38	0.858	0.023	2.714
908443.000	NCI-H520	Lung	Squamous Cell Lung Carcinoma	SN-38 + Olaparib	0.834	0.098	2.714
908443.000	NCI-H520	Lung	Squamous Cell Lung Carcinoma	SRA737 + SN-38	0.819	0.035	2.714
908443.000	NCI-H520	Lung	Squamous Cell Lung Carcinoma	Prexasertib + SN-38	0.900	-0.027	2.714
908444.000	SNU-1	Stomach	Gastric Carcinoma	Prexasertib + SN-38	0.943	-0.035	0.151
908444.000	SNU-1	Stomach	Gastric Carcinoma	SN-38 + Olaparib	0.926	0.019	0.151
908446.000	SNU-16	Stomach	Gastric Carcinoma	SN-38 + Olaparib	0.841	-0.003	0.057
908446.000	SNU-16	Stomach	Gastric Carcinoma	AZD1775 + SN-38	1.000	-0.009	0.057
908446.000	SNU-16	Stomach	Gastric Carcinoma	Prexasertib + SN-38	1.000	0.057	0.057
908446.000	SNU-16	Stomach	Gastric Carcinoma	SN-38 + Olaparib	0.854	0.030	0.057
908446.000	SNU-16	Stomach	Gastric Carcinoma	SRA737 + SN-38	0.995	0.083	0.057
908446.000	SNU-16	Stomach	Gastric Carcinoma	Prexasertib + SN-38	1.000	0.065	0.057
908449.000	NMC-G1	Central Nervous System	Low Grade Glioma	Prexasertib + SN-38	0.318	-0.044	4.856
908449.000	NMC-G1	Central Nervous System	Low Grade Glioma	Prexasertib + SN-38	0.290	-0.045	4.856
908449.000	NMC-G1	Central Nervous System	Low Grade Glioma	SN-38 + Olaparib	0.271	0.028	4.856
908449.000	NMC-G1	Central Nervous System	Low Grade Glioma	SN-38 + Olaparib	0.271	0.004	4.856
908449.000	NMC-G1	Central Nervous System	Low Grade Glioma	SN-38 + Olaparib	0.211	0.019	4.856
908449.000	NMC-G1	Central Nervous System	Low Grade Glioma	Prexasertib + SN-38	0.354	-0.031	4.856
908449.000	NMC-G1	Central Nervous System	Low Grade Glioma	SN-38 + Olaparib	0.240	-0.004	4.856
908449.000	NMC-G1	Central Nervous System	Low Grade Glioma	Prexasertib + SN-38	0.312	-0.048	4.856
908449.000	NMC-G1	Central Nervous System	Low Grade Glioma	SN-38 + Olaparib	0.246	0.017	4.856
908449.000	NMC-G1	Central Nervous System	Low Grade Glioma	Prexasertib + SN-38	0.310	-0.002	4.856
908451.000	NOMO-1	Haematopoietic and Lymphoid	Acute Myeloid Leukemia	Prexasertib + SN-38	1.000	-0.029	5.339
908451.000	NOMO-1	Haematopoietic and Lymphoid	Acute Myeloid Leukemia	SN-38 + Olaparib	0.968	0.058	5.339
908451.000	NOMO-1	Haematopoietic and Lymphoid	Acute Myeloid Leukemia	AZD1775 + SN-38	1.000	0.026	5.339
908451.000	NOMO-1	Haematopoietic and Lymphoid	Acute Myeloid Leukemia	SRA737 + SN-38	0.997	0.005	5.339
908457.000	NCI-H747	Large Intestine	Colorectal Carcinoma	Prexasertib + SN-38	0.725	0.094	0.275
908457.000	NCI-H747	Large Intestine	Colorectal Carcinoma	SN-38 + Olaparib	0.542	0.059	0.275
908460.000	NCI-H441	Lung	Lung Adenocarcinoma	AZD1775 + SN-38	0.689	-0.011	5.332
908460.000	NCI-H441	Lung	Lung Adenocarcinoma	SN-38 + Olaparib	0.709	0.087	5.332
908460.000	NCI-H441	Lung	Lung Adenocarcinoma	Prexasertib + SN-38	0.735	-0.012	5.332
908460.000	NCI-H441	Lung	Lung Adenocarcinoma	SRA737 + SN-38	0.663	0.027	5.332
908461.000	NCI-N87	Stomach	Gastric Carcinoma	SRA737 + SN-38	0.732	-0.007	5.260
908461.000	NCI-N87	Stomach	Gastric Carcinoma	SN-38 + Olaparib	0.722	0.074	5.260
908461.000	NCI-N87	Stomach	Gastric Carcinoma	Prexasertib + SN-38	0.826	-0.007	5.260
908461.000	NCI-N87	Stomach	Gastric Carcinoma	AZD1775 + SN-38	0.814	0.013	5.260
908462.000	NCI-H2452	Lung	Mesothelioma	SRA737 + SN-38	0.787	0.107	5.632
908462.000	NCI-H2452	Lung	Mesothelioma	SN-38 + Olaparib	0.316	0.067	5.632
908462.000	NCI-H2452	Lung	Mesothelioma	AZD1775 + SN-38	0.902	0.036	5.632
908462.000	NCI-H2452	Lung	Mesothelioma	Prexasertib + SN-38	0.895	0.082	5.632
908465.000	NCI-H358	Lung	Lung Adenocarcinoma	SRA737 + SN-38	0.800	0.081	0.251

908465.000	NCI-H358	Lung	Lung Adenocarcinoma	AZD1775 + SN-38	0.821	0.073	0.251
908465.000	NCI-H358	Lung	Lung Adenocarcinoma	SN-38 + Olaparib	0.693	0.026	0.251
908465.000	NCI-H358	Lung	Lung Adenocarcinoma	Prexasertib + SN-38	0.897	0.013	0.251
908467.000	NCI-H1155	Lung	Non-Small Cell Lung Carcinoma	SN-38 + Olaparib	0.968	0.055	5.666
908467.000	NCI-H1155	Lung	Non-Small Cell Lung Carcinoma	Prexasertib + SN-38	0.996	-0.016	5.666
908470.000	NCI-H28	Lung	Mesothelioma	Prexasertib + SN-38	0.655	-0.023	3.369
908470.000	NCI-H28	Lung	Mesothelioma	SN-38 + Olaparib	0.429	0.074	3.369
908470.000	NCI-H28	Lung	Mesothelioma	AZD1775 + SN-38	0.674	0.021	3.369
908470.000	NCI-H28	Lung	Mesothelioma	SRA737 + SN-38	0.516	-0.054	3.369
908471.000	NCI-H1581	Lung	Non-Small Cell Lung Carcinoma	Prexasertib + SN-38	0.905	0.199	3.463
908471.000	NCI-H1581	Lung	Non-Small Cell Lung Carcinoma	SN-38 + Olaparib	0.722	0.025	3.463
908471.000	NCI-H1581	Lung	Non-Small Cell Lung Carcinoma	AZD1775 + SN-38	0.968	0.117	3.463
908471.000	NCI-H1581	Lung	Non-Small Cell Lung Carcinoma	SRA737 + SN-38	0.860	0.151	3.463
908472.000	NCI-H1573	Lung	Lung Adenocarcinoma	Prexasertib + SN-38	0.723	0.050	3.302
908472.000	NCI-H1573	Lung	Lung Adenocarcinoma	SN-38 + Olaparib	0.187	-0.004	3.302
908473.000	NCI-H1666	Lung	Lung Adenocarcinoma	SRA737 + SN-38	0.503	-0.028	1.050
908473.000	NCI-H1666	Lung	Lung Adenocarcinoma	SN-38 + Olaparib	0.532	0.108	1.050
908473.000	NCI-H1666	Lung	Lung Adenocarcinoma	Prexasertib + SN-38	0.626	0.009	1.050
908473.000	NCI-H1666	Lung	Lung Adenocarcinoma	AZD1775 + SN-38	0.623	0.049	1.050
908474.000	NCI-H1703	Lung	Squamous Cell Lung Carcinoma	SN-38 + Olaparib	0.882	0.037	4.282
908474.000	NCI-H1703	Lung	Squamous Cell Lung Carcinoma	AZD1775 + SN-38	1.000	0.008	4.282
908474.000	NCI-H1703	Lung	Squamous Cell Lung Carcinoma	Prexasertib + SN-38	0.970	0.010	4.282
908474.000	NCI-H1703	Lung	Squamous Cell Lung Carcinoma	SRA737 + SN-38	0.929	-0.017	4.282
908475.000	NCI-H1755	Lung	Lung Adenocarcinoma	AZD1775 + SN-38	0.888	0.063	0.163
908475.000	NCI-H1755	Lung	Lung Adenocarcinoma	SN-38 + Olaparib	0.616	0.055	0.163
908475.000	NCI-H1755	Lung	Lung Adenocarcinoma	Prexasertib + SN-38	0.950	0.039	0.163
908475.000	NCI-H1755	Lung	Lung Adenocarcinoma	SRA737 + SN-38	0.871	0.030	0.163
908483.000	NCI-H524	Lung	Small Cell Lung Carcinoma	SRA737 + SN-38	0.938	0.149	0.098
908483.000	NCI-H524	Lung	Small Cell Lung Carcinoma	SN-38 + Olaparib	0.784	0.063	0.098
908483.000	NCI-H524	Lung	Small Cell Lung Carcinoma	Prexasertib + SN-38	1.000	0.062	0.098
908483.000	NCI-H524	Lung	Small Cell Lung Carcinoma	AZD1775 + SN-38	0.845	0.021	0.098
909248.000	ONS-76	Central Nervous System	Other Solid Carcinomas	SRA737 + SN-38	0.876	0.020	0.111
909248.000	ONS-76	Central Nervous System	Other Solid Carcinomas	SN-38 + Olaparib	0.829	0.069	0.111
909248.000	ONS-76	Central Nervous System	Other Solid Carcinomas	AZD1775 + SN-38	0.886	0.040	0.111
909248.000	ONS-76	Central Nervous System	Other Solid Carcinomas	Prexasertib + SN-38	0.886	-0.045	0.111
909249.000	OPM-2	Haematopoietic and Lymphoid	Plasma Cell Myeloma	Prexasertib + SN-38	0.970	0.011	0.227
909249.000	OPM-2	Haematopoietic and Lymphoid	Plasma Cell Myeloma	SN-38 + Olaparib	0.734	0.015	0.227
909249.000	OPM-2	Haematopoietic and Lymphoid	Plasma Cell Myeloma	SRA737 + SN-38	0.926	0.057	0.227
909249.000	OPM-2	Haematopoietic and Lymphoid	Plasma Cell Myeloma	AZD1775 + SN-38	0.995	0.054	0.227
909253.000	P31-FUJ	Haematopoietic and Lymphoid	Acute Myeloid Leukemia	SN-38 + Olaparib	0.987	0.071	5.323
909253.000	P31-FUJ	Haematopoietic and Lymphoid	Acute Myeloid Leukemia	Prexasertib + SN-38	1.000	-0.011	5.323
909253.000	P31-FUJ	Haematopoietic and Lymphoid	Acute Myeloid Leukemia	AZD1775 + SN-38	0.990	0.049	5.323
909253.000	P31-FUJ	Haematopoietic and Lymphoid	Acute Myeloid Leukemia	SRA737 + SN-38	0.983	0.005	5.323
909255.000	PA-1	Ovary	Ovarian Carcinoma	SN-38 + Olaparib	0.848	0.019	3.796
909255.000	PA-1	Ovary	Ovarian Carcinoma	Prexasertib + SN-38	0.848	0.025	3.796
909257.000	OC-314	Ovary	Ovarian Carcinoma	AZD1775 + SN-38	1.000	0.047	6.655
909257.000	OC-314	Ovary	Ovarian Carcinoma	SN-38 + Olaparib	1.000	0.126	6.655
909257.000	OC-314	Ovary	Ovarian Carcinoma	Prexasertib + SN-38	1.000	0.034	6.655
909257.000	OC-314	Ovary	Ovarian Carcinoma	SRA737 + SN-38	0.994	0.013	6.655
909260.000	PF-382	Haematopoietic and Lymphoid	T-Lymphoblastic Leukemia	Prexasertib + SN-38	0.987	-0.032	6.320
909260.000	PF-382	Haematopoietic and Lymphoid	T-Lymphoblastic Leukemia	SN-38 + Olaparib	1.000	0.056	6.320
909262.000	Raji	Haematopoietic and Lymphoid	Burkitt's Lymphoma	SN-38 + Olaparib	0.956	0.042	4.608
909262.000	Raji	Haematopoietic and Lymphoid	Burkitt's Lymphoma	Prexasertib + SN-38	0.982	-0.020	4.608
909263.000	RCM-1	Large Intestine	Colorectal Carcinoma	SRA737 + SN-38	0.768	0.015	0.043
909263.000	RCM-1	Large Intestine	Colorectal Carcinoma	AZD1775 + SN-38	0.796	0.034	0.043
909263.000	RCM-1	Large Intestine	Colorectal Carcinoma	SN-38 + Olaparib	0.809	-0.006	0.043
909263.000	RCM-1	Large Intestine	Colorectal Carcinoma	Prexasertib + SN-38	0.878	-0.002	0.043
909696.000	REH	Haematopoietic and Lymphoid	B-Lymphoblastic Leukemia	AZD1775 + SN-38	0.996	-0.036	6.564
909696.000	REH	Haematopoietic and Lymphoid	B-Lymphoblastic Leukemia	SRA737 + SN-38	0.991	-0.016	6.564
909696.000	REH	Haematopoietic and Lymphoid	B-Lymphoblastic Leukemia	SN-38 + Olaparib	1.000	0.056	6.564
909696.000	REH	Haematopoietic and Lymphoid	B-Lymphoblastic Leukemia	Prexasertib + SN-38	1.000	0.009	6.564
909698.000	RKO	Large Intestine	Colorectal Carcinoma	SRA737 + SN-38	0.925	0.009	0.029
909698.000	RKO	Large Intestine	Colorectal Carcinoma	Prexasertib + SN-38	0.933	-0.022	0.029
909698.000	RKO	Large Intestine	Colorectal Carcinoma	SN-38 + Olaparib	0.860	0.033	0.029
909698.000	RKO	Large Intestine	Colorectal Carcinoma	AZD1775 + SN-38	0.975	0.038	0.029
909699.000	RMG-I	Ovary	Ovarian Carcinoma	AZD1775 + SN-38	0.724	0.037	0.057
909699.000	RMG-I	Ovary	Ovarian Carcinoma	SRA737 + SN-38	0.484	0.015	0.057
909699.000	RMG-I	Ovary	Ovarian Carcinoma	SN-38 + Olaparib	0.421	0.027	0.057
909699.000	RMG-I	Ovary	Ovarian Carcinoma	Prexasertib + SN-38	0.545	-0.064	0.057
909702.000	RPMI-8402	Haematopoietic and Lymphoid	T-Lymphoblastic Leukemia	AZD1775 + SN-38	1.000	0.038	5.678
909702.000	RPMI-8402	Haematopoietic and Lymphoid	T-Lymphoblastic Leukemia	Prexasertib + SN-38	1.000	0.014	5.678
909702.000	RPMI-8402	Haematopoietic and Lymphoid	T-Lymphoblastic Leukemia	SN-38 + Olaparib	1.000	0.098	5.678
909702.000	RPMI-8402	Haematopoietic and Lymphoid	T-Lymphoblastic Leukemia	SRA737 + SN-38	1.000	0.016	5.678
909704.000	RT-112	Bladder	Bladder Carcinoma	SN-38 + Olaparib	0.762	0.123	0.124
909704.000	RT-112	Bladder	Bladder Carcinoma	Prexasertib + SN-38	1.000	0.127	0.124
909704.000	RT-112	Bladder	Bladder Carcinoma	AZD1775 + SN-38	0.985	0.148	0.124
909704.000	RT-112	Bladder	Bladder Carcinoma	SRA737 + SN-38	0.950	0.160	0.124
909706.000	RVH-421	Skin	Melanoma	Prexasertib + SN-38	0.355	-0.073	0.057
909706.000	RVH-421	Skin	Melanoma	SN-38 + Olaparib	0.162	-0.017	0.057
909706.000	RVH-421	Skin	Melanoma	SN-38 + Olaparib	0.367	0.050	0.057
909706.000	RVH-421	Skin	Melanoma	Prexasertib + SN-38	0.418	-0.041	0.057
909707.000	Saos-2	Bone	Osteosarcoma	Prexasertib + SN-38	0.984	0.064	4.938
909707.000	Saos-2	Bone	Osteosarcoma	SN-38 + Olaparib	0.960	0.041	4.938
909707.000	Saos-2	Bone	Osteosarcoma	SRA737 + SN-38	0.948	0.008	4.938
909707.000	Saos-2	Bone	Osteosarcoma	AZD1775 + SN-38	0.970	0.028	4.938
909709.000	SCC-9	Head and Neck	Oral Cavity Carcinoma	AZD1775 + SN-38	0.702	0.048	0.333
909709.000	SCC-9	Head and Neck	Oral Cavity Carcinoma	SRA737 + SN-38	0.518	0.055	0.333
909709.000	SCC-9	Head and Neck	Oral Cavity Carcinoma	Prexasertib + SN-38	0.577	0.081	0.333
909709.000	SCC-9	Head and Neck	Oral Cavity Carcinoma	SN-38 + Olaparib	0.365	0.035	0.333
909713.000	SH-4	Skin	Melanoma	Prexasertib + SN-38	0.690	-0.048	0.227
909713.000	SH-4	Skin	Melanoma	SN-38 + Olaparib	0.480	0.108	0.227
909713.000	SH-4	Skin	Melanoma	AZD1775 + SN-38	0.683	0.004	0.227
909713.000	SH-4	Skin	Melanoma	SRA737 + SN-38	0.518	-0.064	0.227
909717.000	SJSA-1	Bone	Osteosarcoma	SRA737 + SN-38	0.664	-0.010	4.661
909717.000	SJSA-1	Bone	Osteosarcoma	SN-38 + Olaparib	0.804	0.064	4.661
909717.000	SJSA-1	Bone	Osteosarcoma	Prexasertib + SN-38	0.758	-0.066	4.661
909717.000	SJSA-1	Bone	Osteosarcoma	AZD1775 + SN-38	0.706	0.014	4.661
909718.000	SK-CO-1	Large Intestine	Colorectal Carcinoma	Prexasertib + SN-38	0.798	0.007	5.029
909718.000	SK-CO-1	Large Intestine	Colorectal Carcinoma	SRA737 + SN-38	0.893	-0.044	5.029
909718.000	SK-CO-1	Large Intestine	Colorectal Carcinoma	AZD1775 + SN-38	0.899	0.021	5.029
909718.000	SK-CO-1	Large Intestine	Colorectal Carcinoma	SN-38 + Olaparib	0.798	0.062	5.029
909719.000	SK-HEP-1	Liver	Hepatocellular Carcinoma	SRA737 + SN-38	0.802	-0.019	0.138
909719.000	SK-HEP-1	Liver	Hepatocellular Carcinoma	Prexasertib + SN-38	0.780	0.009	0.138
909719.000	SK-HEP-1	Liver	Hepatocellular Carcinoma	SN-38 + Olaparib	0.665	0.045	0.138
909719.000	SK-HEP-1	Liver	Hepatocellular Carcinoma	AZD1775 + SN-38	0.637	0.056	0.138

909720.000	SK-LMS-1	Soft Tissue	Other Solid Carcinomas	SRA737 + SN-38	0.890	-0.009	4.911
909720.000	SK-LMS-1	Soft Tissue	Other Solid Carcinomas	Prexasertib + SN-38	0.895	0.017	4.911
909720.000	SK-LMS-1	Soft Tissue	Other Solid Carcinomas	SN-38 + Olaparib	0.889	0.036	4.911
909720.000	SK-LMS-1	Soft Tissue	Other Solid Carcinomas	AZD1775 + SN-38	0.917	0.018	4.911
909721.000	SK-LU-1	Lung	Lung Adenocarcinoma	SRA737 + SN-38	0.639	-0.015	3.845
909721.000	SK-LU-1	Lung	Lung Adenocarcinoma	AZD1775 + SN-38	0.645	-0.037	3.845
909723.000	SK-MEL-1	Skin	Melanoma	AZD1775 + SN-38	0.560	0.079	0.057
909723.000	SK-MEL-1	Skin	Melanoma	SRA737 + SN-38	0.486	0.034	0.057
909723.000	SK-MEL-1	Skin	Melanoma	Prexasertib + SN-38	0.628	-0.031	0.057
909723.000	SK-MEL-1	Skin	Melanoma	SN-38 + Olaparib	0.516	0.045	0.057
909725.000	SK-MEL-24	Skin	Melanoma	AZD1775 + SN-38	0.807	0.005	5.326
909725.000	SK-MEL-24	Skin	Melanoma	SRA737 + SN-38	0.730	0.014	5.326
909725.000	SK-MEL-24	Skin	Melanoma	SN-38 + Olaparib	0.775	0.039	5.326
909725.000	SK-MEL-24	Skin	Melanoma	Prexasertib + SN-38	0.868	0.038	5.326
909731.000	SK-PN-DW	Central Nervous System	Other Solid Carcinomas	SN-38 + Olaparib	0.909	0.094	6.520
909731.000	SK-PN-DW	Central Nervous System	Other Solid Carcinomas	Prexasertib + SN-38	0.957	-0.027	6.520
909731.000	SK-PN-DW	Central Nervous System	Other Solid Carcinomas	AZD1775 + SN-38	0.952	-0.021	6.520
909731.000	SK-PN-DW	Central Nervous System	Other Solid Carcinomas	SRA737 + SN-38	0.927	0.025	6.520
909732.000	SK-UT-1	Uterus	Other Solid Carcinomas	Prexasertib + SN-38	0.976	0.011	4.320
909732.000	SK-UT-1	Uterus	Other Solid Carcinomas	SRA737 + SN-38	0.943	0.041	4.320
909732.000	SK-UT-1	Uterus	Other Solid Carcinomas	SN-38 + Olaparib	0.972	0.061	4.320
909732.000	SK-UT-1	Uterus	Other Solid Carcinomas	AZD1775 + SN-38	0.994	0.033	4.320
909735.000	SNG-M	Endometrium	Endometrial Carcinoma	SRA737 + SN-38	0.786	-0.042	0.151
909735.000	SNG-M	Endometrium	Endometrial Carcinoma	SN-38 + Olaparib	0.620	0.073	0.151
909735.000	SNG-M	Endometrium	Endometrial Carcinoma	Prexasertib + SN-38	0.949	-0.047	0.151
909735.000	SNG-M	Endometrium	Endometrial Carcinoma	AZD1775 + SN-38	0.933	0.045	0.151
909736.000	SNU-387	Liver	Hepatocellular Carcinoma	Prexasertib + SN-38	0.716	0.082	0.390
909736.000	SNU-387	Liver	Hepatocellular Carcinoma	AZD1775 + SN-38	0.484	0.025	0.390
909736.000	SNU-387	Liver	Hepatocellular Carcinoma	AZD1775 + SN-38	0.519	-0.002	0.390
909736.000	SNU-387	Liver	Hepatocellular Carcinoma	SRA737 + SN-38	0.543	0.066	0.390
909736.000	SNU-387	Liver	Hepatocellular Carcinoma	SN-38 + Olaparib	0.146	-0.023	0.390
909736.000	SNU-387	Liver	Hepatocellular Carcinoma	SRA737 + SN-38	0.553	0.036	0.390
909737.000	SNU-423	Liver	Hepatocellular Carcinoma	SRA737 + SN-38	0.648	0.070	3.962
909737.000	SNU-423	Liver	Hepatocellular Carcinoma	AZD1775 + SN-38	0.793	0.034	3.962
909737.000	SNU-423	Liver	Hepatocellular Carcinoma	Prexasertib + SN-38	0.804	0.095	3.962
909737.000	SNU-423	Liver	Hepatocellular Carcinoma	SN-38 + Olaparib	0.495	0.016	3.962
909738.000	SNU-449	Liver	Hepatocellular Carcinoma	SN-38 + Olaparib	0.201	-0.012	0.189
909738.000	SNU-449	Liver	Hepatocellular Carcinoma	Prexasertib + SN-38	0.655	0.024	0.189
909738.000	SNU-449	Liver	Hepatocellular Carcinoma	SRA737 + SN-38	0.704	0.072	0.189
909738.000	SNU-449	Liver	Hepatocellular Carcinoma	Prexasertib + SN-38	0.510	0.026	0.189
909738.000	SNU-449	Liver	Hepatocellular Carcinoma	SN-38 + Olaparib	0.149	-0.013	0.189
909738.000	SNU-449	Liver	Hepatocellular Carcinoma	AZD1775 + SN-38	0.737	0.046	0.189
909743.000	SUP-T1	Haematopoietic and Lymphoid	T-Lymphoblastic Leukemia	AZD1775 + SN-38	0.880	-0.041	6.804
909743.000	SUP-T1	Haematopoietic and Lymphoid	T-Lymphoblastic Leukemia	SRA737 + SN-38	0.796	-0.046	6.804
909743.000	SUP-T1	Haematopoietic and Lymphoid	T-Lymphoblastic Leukemia	SN-38 + Olaparib	0.840	0.081	6.804
909743.000	SUP-T1	Haematopoietic and Lymphoid	T-Lymphoblastic Leukemia	Prexasertib + SN-38	0.858	-0.095	6.804
909744.000	SW13	Adrenal Gland	Other Solid Carcinomas	SRA737 + SN-38	0.948	-0.010	5.239
909744.000	SW13	Adrenal Gland	Other Solid Carcinomas	SN-38 + Olaparib	0.962	0.049	5.239
909744.000	SW13	Adrenal Gland	Other Solid Carcinomas	Prexasertib + SN-38	0.995	0.023	5.239
909744.000	SW13	Adrenal Gland	Other Solid Carcinomas	AZD1775 + SN-38	0.973	0.080	5.239
909745.000	SW1088	Central Nervous System	Low Grade Glioma	SRA737 + SN-38	0.736	0.041	0.918
909745.000	SW1088	Central Nervous System	Low Grade Glioma	AZD1775 + SN-38	0.783	0.024	0.918
909745.000	SW1088	Central Nervous System	Low Grade Glioma	Prexasertib + SN-38	0.872	0.066	0.918
909745.000	SW1088	Central Nervous System	Low Grade Glioma	SN-38 + Olaparib	0.434	0.094	0.918
909746.000	SW1116	Large Intestine	Colorectal Carcinoma	SRA737 + SN-38	0.616	0.058	0.433
909746.000	SW1116	Large Intestine	Colorectal Carcinoma	AZD1775 + SN-38	0.708	0.001	0.433
909746.000	SW1116	Large Intestine	Colorectal Carcinoma	Prexasertib + SN-38	0.745	0.075	0.433
909746.000	SW1116	Large Intestine	Colorectal Carcinoma	SN-38 + Olaparib	0.214	0.022	0.433
909747.000	SW1417	Large Intestine	Colorectal Carcinoma	Prexasertib + SN-38	0.859	0.055	0.189
909747.000	SW1417	Large Intestine	Colorectal Carcinoma	SRA737 + SN-38	0.804	0.036	0.189
909747.000	SW1417	Large Intestine	Colorectal Carcinoma	SN-38 + Olaparib	0.694	0.038	0.189
909747.000	SW1417	Large Intestine	Colorectal Carcinoma	AZD1775 + SN-38	0.808	0.043	0.189
909748.000	SW1463	Large Intestine	Colorectal Carcinoma	SRA737 + SN-38	0.758	0.121	0.575
909748.000	SW1463	Large Intestine	Colorectal Carcinoma	AZD1775 + SN-38	0.759	0.037	0.575
909748.000	SW1463	Large Intestine	Colorectal Carcinoma	SN-38 + Olaparib	0.473	0.067	0.575
909748.000	SW1463	Large Intestine	Colorectal Carcinoma	Prexasertib + SN-38	0.815	0.085	0.575
909749.000	SW1710	Bladder	Bladder Carcinoma	Prexasertib + SN-38	0.871	0.076	0.465
909749.000	SW1710	Bladder	Bladder Carcinoma	AZD1775 + SN-38	0.746	0.049	0.465
909749.000	SW1710	Bladder	Bladder Carcinoma	SN-38 + Olaparib	0.327	0.045	0.465
909749.000	SW1710	Bladder	Bladder Carcinoma	SRA737 + SN-38	0.676	0.102	0.465
909750.000	SW1783	Central Nervous System	Low Grade Glioma	SN-38 + Olaparib	0.823	0.065	4.573
909750.000	SW1783	Central Nervous System	Low Grade Glioma	Prexasertib + SN-38	0.806	-0.038	4.573
909750.000	SW1783	Central Nervous System	Low Grade Glioma	SN-38 + Olaparib	0.900	0.086	4.573
909750.000	SW1783	Central Nervous System	Low Grade Glioma	SRA737 + SN-38	0.872	0.028	4.573
909750.000	SW1783	Central Nervous System	Low Grade Glioma	Prexasertib + SN-38	0.850	-0.063	4.573
909750.000	SW1783	Central Nervous System	Low Grade Glioma	SRA737 + SN-38	0.843	-0.026	4.573
909750.000	SW1783	Central Nervous System	Low Grade Glioma	AZD1775 + SN-38	0.838	-0.018	4.573
909750.000	SW1783	Central Nervous System	Low Grade Glioma	AZD1775 + SN-38	0.819	-0.005	4.573
909751.000	SW48	Large Intestine	Colorectal Carcinoma	SN-38 + Olaparib	0.799	0.034	0.345
909751.000	SW48	Large Intestine	Colorectal Carcinoma	Prexasertib + SN-38	0.881	0.045	0.345
909751.000	SW48	Large Intestine	Colorectal Carcinoma	SRA737 + SN-38	0.854	0.002	0.345
909751.000	SW48	Large Intestine	Colorectal Carcinoma	AZD1775 + SN-38	0.907	0.059	0.345
909753.000	SW626	Large Intestine	Colorectal Carcinoma	Prexasertib + SN-38	0.712	0.015	0.000
909753.000	SW626	Large Intestine	Colorectal Carcinoma	SN-38 + Olaparib	0.559	0.023	0.000
909755.000	SW837	Large Intestine	Colorectal Carcinoma	SN-38 + Olaparib	0.507	0.042	0.705
909755.000	SW837	Large Intestine	Colorectal Carcinoma	SRA737 + SN-38	0.805	0.077	0.705
909755.000	SW837	Large Intestine	Colorectal Carcinoma	Prexasertib + SN-38	0.834	0.084	0.705
909755.000	SW837	Large Intestine	Colorectal Carcinoma	AZD1775 + SN-38	0.875	0.061	0.705
909757.000	SW948	Large Intestine	Colorectal Carcinoma	SRA737 + SN-38	0.834	0.187	0.029
909757.000	SW948	Large Intestine	Colorectal Carcinoma	Prexasertib + SN-38	0.938	0.169	0.029
909757.000	SW948	Large Intestine	Colorectal Carcinoma	AZD1775 + SN-38	0.885	0.137	0.029
909757.000	SW948	Large Intestine	Colorectal Carcinoma	SN-38 + Olaparib	0.647	0.104	0.029
909759.000	SW982	Soft Tissue	Other Solid Carcinomas	SRA737 + SN-38	0.829	-0.049	4.656
909759.000	SW982	Soft Tissue	Other Solid Carcinomas	Prexasertib + SN-38	0.818	-0.002	4.656
909759.000	SW982	Soft Tissue	Other Solid Carcinomas	SN-38 + Olaparib	0.789	0.088	4.656
909759.000	SW982	Soft Tissue	Other Solid Carcinomas	AZD1775 + SN-38	0.866	0.020	4.656
909761.000	T84	Large Intestine	Colorectal Carcinoma	AZD1775 + SN-38	0.913	0.114	0.275
909761.000	T84	Large Intestine	Colorectal Carcinoma	SRA737 + SN-38	0.815	0.039	0.275
909761.000	T84	Large Intestine	Colorectal Carcinoma	SN-38 + Olaparib	0.528	-0.020	0.275
909761.000	T84	Large Intestine	Colorectal Carcinoma	Prexasertib + SN-38	0.857	0.075	0.275
909770.000	TGBC11TKB	Stomach	Gastric Carcinoma	SRA737 + SN-38	0.875	0.057	0.856
909770.000	TGBC11TKB	Stomach	Gastric Carcinoma	AZD1775 + SN-38	0.938	0.027	0.856
909770.000	TGBC11TKB	Stomach	Gastric Carcinoma	Prexasertib + SN-38	0.975	0.054	0.856
909770.000	TGBC11TKB	Stomach	Gastric Carcinoma	SN-38 + Olaparib	0.792	0.098	0.856
909774.000	TYK-nu	Ovary	Ovarian Carcinoma	SN-38 + Olaparib	0.766	0.029	5.961

909774.000	TYK-nu	Ovary	Ovarian Carcinoma	Prexasertib + SN-38	0.845	0.016	5.961
909776.000	U-2-OS	Bone	Osteosarcoma	SN-38 + Olaparib	0.209	0.019	0.098
909776.000	U-2-OS	Bone	Osteosarcoma	Prexasertib + SN-38	0.845	-0.049	0.098
909776.000	U-2-OS	Bone	Osteosarcoma	SN-38 + Olaparib	0.146	-0.007	0.098
909776.000	U-2-OS	Bone	Osteosarcoma	Prexasertib + SN-38	0.864	-0.062	0.098
909776.000	U-2-OS	Bone	Osteosarcoma	SN-38 + Olaparib	0.205	0.020	0.098
909776.000	U-2-OS	Bone	Osteosarcoma	SN-38 + Olaparib	0.296	0.032	0.098
909776.000	U-2-OS	Bone	Osteosarcoma	AZD1775 + SN-38	0.893	-0.004	0.098
909776.000	U-2-OS	Bone	Osteosarcoma	Prexasertib + SN-38	0.872	-0.104	0.098
909776.000	U-2-OS	Bone	Osteosarcoma	SN-38 + Olaparib	0.189	-0.006	0.098
909776.000	U-2-OS	Bone	Osteosarcoma	Prexasertib + SN-38	0.886	-0.047	0.098
909776.000	U-2-OS	Bone	Osteosarcoma	Prexasertib + SN-38	0.865	-0.054	0.098
909776.000	U-2-OS	Bone	Osteosarcoma	SRA737 + SN-38	0.583	-0.028	0.098
909776.000	U-2-OS	Bone	Osteosarcoma	SN-38 + Olaparib	0.293	0.023	0.098
909776.000	U-2-OS	Bone	Osteosarcoma	Prexasertib + SN-38	0.877	-0.040	0.098
909776.000	U-2-OS	Bone	Osteosarcoma	SN-38 + Olaparib	0.275	0.056	0.098
909776.000	U-2-OS	Bone	Osteosarcoma	Prexasertib + SN-38	0.867	-0.058	0.098
909776.000	U-2-OS	Bone	Osteosarcoma	AZD1775 + SN-38	0.910	0.018	0.098
909776.000	U-2-OS	Bone	Osteosarcoma	Prexasertib + SN-38	0.875	-0.071	0.098
909776.000	U-2-OS	Bone	Osteosarcoma	SRA737 + SN-38	0.616	-0.050	0.098
909776.000	U-2-OS	Bone	Osteosarcoma	Prexasertib + SN-38	0.886	-0.042	0.098
909776.000	U-2-OS	Bone	Osteosarcoma	Prexasertib + SN-38	0.865	-0.078	0.098
909776.000	U-2-OS	Bone	Osteosarcoma	SN-38 + Olaparib	0.144	0.000	0.098
909776.000	U-2-OS	Bone	Osteosarcoma	SN-38 + Olaparib	0.076	-0.012	0.098
909776.000	U-2-OS	Bone	Osteosarcoma	Prexasertib + SN-38	0.877	-0.035	0.098
909776.000	U-2-OS	Bone	Osteosarcoma	SN-38 + Olaparib	0.239	0.024	0.098
909776.000	U-2-OS	Bone	Osteosarcoma	AZD1775 + SN-38	0.940	0.046	0.098
909776.000	U-2-OS	Bone	Osteosarcoma	SRA737 + SN-38	0.610	-0.031	0.098
909776.000	U-2-OS	Bone	Osteosarcoma	SRA737 + SN-38	0.636	-0.020	0.098
909776.000	U-2-OS	Bone	Osteosarcoma	SN-38 + Olaparib	0.326	0.061	0.098
909776.000	U-2-OS	Bone	Osteosarcoma	SN-38 + Olaparib	0.166	0.000	0.098
909776.000	U-2-OS	Bone	Osteosarcoma	SN-38 + Olaparib	0.317	0.057	0.098
909776.000	U-2-OS	Bone	Osteosarcoma	AZD1775 + SN-38	0.928	-0.015	0.098
909776.000	U-2-OS	Bone	Osteosarcoma	SRA737 + SN-38	0.707	-0.008	0.098
909776.000	U-2-OS	Bone	Osteosarcoma	Prexasertib + SN-38	0.889	-0.040	0.098
909776.000	U-2-OS	Bone	Osteosarcoma	Prexasertib + SN-38	0.876	-0.027	0.098
909776.000	U-2-OS	Bone	Osteosarcoma	Prexasertib + SN-38	0.853	-0.072	0.098
909776.000	U-2-OS	Bone	Osteosarcoma	AZD1775 + SN-38	0.895	0.025	0.098
909776.000	U-2-OS	Bone	Osteosarcoma	AZD1775 + SN-38	0.963	0.032	0.098
909776.000	U-2-OS	Bone	Osteosarcoma	SN-38 + Olaparib	0.162	0.002	0.098
909776.000	U-2-OS	Bone	Osteosarcoma	SRA737 + SN-38	0.591	-0.012	0.098
909776.000	U-2-OS	Bone	Osteosarcoma	Prexasertib + SN-38	0.866	-0.099	0.098
909776.000	U-2-OS	Bone	Osteosarcoma	SN-38 + Olaparib	0.180	0.016	0.098
909776.000	U-2-OS	Bone	Osteosarcoma	AZD1775 + SN-38	0.916	0.034	0.098
909776.000	U-2-OS	Bone	Osteosarcoma	SRA737 + SN-38	0.603	-0.029	0.098
909780.000	VM-CUB-1	Bladder	Bladder Carcinoma	SN-38 + Olaparib	1.000	0.054	1.333
909780.000	VM-CUB-1	Bladder	Bladder Carcinoma	SRA737 + SN-38	1.000	-0.012	1.333
909780.000	VM-CUB-1	Bladder	Bladder Carcinoma	AZD1775 + SN-38	1.000	0.012	1.333
909780.000	VM-CUB-1	Bladder	Bladder Carcinoma	Prexasertib + SN-38	1.000	0.000	1.333
909781.000	VMRC-RCZ	Kidney	Kidney Carcinoma	Prexasertib + SN-38	0.626	0.057	1.299
909781.000	VMRC-RCZ	Kidney	Kidney Carcinoma	AZD1775 + SN-38	0.611	0.021	1.299
909781.000	VMRC-RCZ	Kidney	Kidney Carcinoma	SN-38 + Olaparib	0.281	0.025	1.299
909781.000	VMRC-RCZ	Kidney	Kidney Carcinoma	SRA737 + SN-38	0.579	0.003	1.299
909784.000	WM-115	Skin	Melanoma	SRA737 + SN-38	0.421	-0.044	4.512
909784.000	WM-115	Skin	Melanoma	SN-38 + Olaparib	0.391	0.100	4.512
909784.000	WM-115	Skin	Melanoma	Prexasertib + SN-38	0.590	-0.015	4.512
909784.000	WM-115	Skin	Melanoma	AZD1775 + SN-38	0.416	0.040	4.512
909904.000	YAPC	Pancreas	Pancreatic Carcinoma	SN-38 + Olaparib	0.646	0.053	5.552
909904.000	YAPC	Pancreas	Pancreatic Carcinoma	AZD1775 + SN-38	0.726	0.012	5.552
909904.000	YAPC	Pancreas	Pancreatic Carcinoma	SRA737 + SN-38	0.696	-0.019	5.552
909904.000	YAPC	Pancreas	Pancreatic Carcinoma	Prexasertib + SN-38	0.672	-0.020	5.552
909905.000	YH-13	Central Nervous System	Glioblastoma	AZD1775 + SN-38	0.790	0.042	1.084
909905.000	YH-13	Central Nervous System	Glioblastoma	SN-38 + Olaparib	0.580	0.108	1.084
909905.000	YH-13	Central Nervous System	Glioblastoma	Prexasertib + SN-38	0.869	0.052	1.084
909905.000	YH-13	Central Nervous System	Glioblastoma	SRA737 + SN-38	0.832	0.069	1.084
909907.000	ZR-75-30	Breast	Breast Carcinoma	AZD1775 + SN-38	0.241	-0.025	0.138
909907.000	ZR-75-30	Breast	Breast Carcinoma	SRA737 + SN-38	0.124	-0.026	0.138
909907.000	ZR-75-30	Breast	Breast Carcinoma	Prexasertib + SN-38	0.174	-0.019	0.138
909907.000	ZR-75-30	Breast	Breast Carcinoma	SN-38 + Olaparib	0.348	0.000	0.138
909975.000	KURAMOCHI	Ovary	Ovarian Carcinoma	SRA737 + SN-38	0.900	0.071	0.163
909975.000	KURAMOCHI	Ovary	Ovarian Carcinoma	Prexasertib + SN-38	0.912	0.085	0.163
909975.000	KURAMOCHI	Ovary	Ovarian Carcinoma	SN-38 + Olaparib	0.651	-0.004	0.163
909975.000	KURAMOCHI	Ovary	Ovarian Carcinoma	AZD1775 + SN-38	0.952	0.089	0.163
910079.000	OE19	Esophagus	Esophageal Adenocarcinoma	Prexasertib + SN-38	0.958	0.160	0.070
910079.000	OE19	Esophagus	Esophageal Adenocarcinoma	SN-38 + Olaparib	0.309	0.013	0.070
910546.000	PSN1	Pancreas	Pancreatic Carcinoma	Prexasertib + SN-38	0.920	0.094	2.325
910546.000	PSN1	Pancreas	Pancreatic Carcinoma	SN-38 + Olaparib	0.753	0.049	2.325
910546.000	PSN1	Pancreas	Pancreatic Carcinoma	AZD1775 + SN-38	0.972	0.053	2.325
910546.000	PSN1	Pancreas	Pancreatic Carcinoma	SRA737 + SN-38	0.908	0.021	2.325
910549.000	OE33	Esophagus	Esophageal Adenocarcinoma	SN-38 + Olaparib	0.587	0.043	3.440
910549.000	OE33	Esophagus	Esophageal Adenocarcinoma	Prexasertib + SN-38	0.844	0.048	3.440
910549.000	OE33	Esophagus	Esophageal Adenocarcinoma	AZD1775 + SN-38	0.812	0.028	3.440
910549.000	OE33	Esophagus	Esophageal Adenocarcinoma	SRA737 + SN-38	0.800	0.046	3.440
910554.000	CW-2	Large Intestine	Colorectal Carcinoma	SRA737 + SN-38	0.625	-0.043	0.138
910554.000	CW-2	Large Intestine	Colorectal Carcinoma	AZD1775 + SN-38	0.798	-0.002	0.138
910554.000	CW-2	Large Intestine	Colorectal Carcinoma	Prexasertib + SN-38	0.651	0.012	0.138
910554.000	CW-2	Large Intestine	Colorectal Carcinoma	SN-38 + Olaparib	0.604	0.037	0.138
910689.000	COLO-678	Large Intestine	Colorectal Carcinoma	Prexasertib + SN-38	0.286	-0.004	0.057
910689.000	COLO-678	Large Intestine	Colorectal Carcinoma	SN-38 + Olaparib	0.396	0.063	0.057
910691.000	COLO-684	Endometrium	Endometrial Carcinoma	AZD1775 + SN-38	0.916	0.066	0.263
910691.000	COLO-684	Endometrium	Endometrial Carcinoma	SRA737 + SN-38	0.878	0.088	0.263
910691.000	COLO-684	Endometrium	Endometrial Carcinoma	SN-38 + Olaparib	0.514	0.000	0.263
910691.000	COLO-684	Endometrium	Endometrial Carcinoma	Prexasertib + SN-38	0.935	0.082	0.263
910692.000	COLO-668	Lung	Small Cell Lung Carcinoma	SN-38 + Olaparib	1.000	0.079	6.306
910692.000	COLO-668	Lung	Small Cell Lung Carcinoma	SRA737 + SN-38	0.953	-0.009	6.306
910692.000	COLO-668	Lung	Small Cell Lung Carcinoma	Prexasertib + SN-38	0.984	-0.037	6.306
910692.000	COLO-668	Lung	Small Cell Lung Carcinoma	AZD1775 + SN-38	0.963	0.049	6.306
910698.000	BFTC-909	Kidney	Kidney Carcinoma	SN-38 + Olaparib	0.931	0.094	4.324
910698.000	BFTC-909	Kidney	Kidney Carcinoma	Prexasertib + SN-38	0.991	0.050	4.324
910702.000	AsPC-1	Pancreas	Pancreatic Carcinoma	Prexasertib + SN-38	0.369	-0.010	0.189
910702.000	AsPC-1	Pancreas	Pancreatic Carcinoma	SN-38 + Olaparib	0.156	-0.016	0.189
910704.000	AU565	Breast	Breast Carcinoma	SN-38 + Olaparib	0.891	0.083	4.673
910704.000	AU565	Breast	Breast Carcinoma	Prexasertib + SN-38	0.927	0.022	4.673
910706.000	BL-41	Haematopoietic and Lymphoid	Burkitt's Lymphoma	SRA737 + SN-38	1.000	-0.005	4.211
910706.000	BL-41	Haematopoietic and Lymphoid	Burkitt's Lymphoma	AZD1775 + SN-38	1.000	-0.001	4.211

910706.000	BL-41	Haematopoietic and Lymphoid	Burkitt's Lymphoma	Prexasertib + SN-38	1.000	0.007	4.211
910706.000	BL-41	Haematopoietic and Lymphoid	Burkitt's Lymphoma	SN-38 + Olaparib	1.000	0.048	4.211
910779.000	IA-LM	Lung	Non-Small Cell Lung Carcinoma	AZD1775 + SN-38	0.713	0.030	2.322
910779.000	IA-LM	Lung	Non-Small Cell Lung Carcinoma	Prexasertib + SN-38	0.907	0.042	2.322
910779.000	IA-LM	Lung	Non-Small Cell Lung Carcinoma	SRA737 + SN-38	0.675	-0.022	2.322
910779.000	IA-LM	Lung	Non-Small Cell Lung Carcinoma	SN-38 + Olaparib	0.878	0.068	2.322
910780.000	TGW	Peripheral Nervous System	Neuroblastoma	AZD1775 + SN-38	0.865	0.014	0.000
910780.000	TGW	Peripheral Nervous System	Neuroblastoma	SRA737 + SN-38	0.713	0.066	0.000
910780.000	TGW	Peripheral Nervous System	Neuroblastoma	SN-38 + Olaparib	0.669	0.076	0.000
910780.000	TGW	Peripheral Nervous System	Neuroblastoma	Prexasertib + SN-38	0.937	-0.022	0.000
910852.000	CAL-85-1	Breast	Breast Carcinoma	Prexasertib + SN-38	0.863	0.113	3.205
910852.000	CAL-85-1	Breast	Breast Carcinoma	AZD1775 + SN-38	0.931	0.083	3.205
910852.000	CAL-85-1	Breast	Breast Carcinoma	SN-38 + Olaparib	0.702	0.080	3.205
910852.000	CAL-85-1	Breast	Breast Carcinoma	SRA737 + SN-38	0.746	-0.013	3.205
910900.000	NCI-H1651	Lung	Lung Adenocarcinoma	AZD1775 + SN-38	0.941	0.039	5.936
910900.000	NCI-H1651	Lung	Lung Adenocarcinoma	SN-38 + Olaparib	0.903	0.043	5.936
910900.000	NCI-H1651	Lung	Lung Adenocarcinoma	Prexasertib + SN-38	0.887	0.017	5.936
910900.000	NCI-H1651	Lung	Lung Adenocarcinoma	SRA737 + SN-38	0.835	0.036	5.936
910903.000	RPMI-7951	Skin	Melanoma	Prexasertib + SN-38	0.995	-0.008	4.791
910903.000	RPMI-7951	Skin	Melanoma	SN-38 + Olaparib	0.948	0.076	4.791
910904.000	SCC-4	Head and Neck	Oral Cavity Carcinoma	Prexasertib + SN-38	0.893	0.071	2.493
910904.000	SCC-4	Head and Neck	Oral Cavity Carcinoma	SN-38 + Olaparib	0.847	0.023	2.493
910904.000	SCC-4	Head and Neck	Oral Cavity Carcinoma	AZD1775 + SN-38	0.909	0.009	2.493
910904.000	SCC-4	Head and Neck	Oral Cavity Carcinoma	SRA737 + SN-38	0.893	0.055	2.493
910905.000	SNU-C1	Large Intestine	Colorectal Carcinoma	AZD1775 + SN-38	0.772	-0.011	0.138
910905.000	SNU-C1	Large Intestine	Colorectal Carcinoma	Prexasertib + SN-38	0.783	0.010	0.138
910905.000	SNU-C1	Large Intestine	Colorectal Carcinoma	SN-38 + Olaparib	0.349	-0.030	0.138
910905.000	SNU-C1	Large Intestine	Colorectal Carcinoma	SRA737 + SN-38	0.748	0.117	0.138
910906.000	ST486	Haematopoietic and Lymphoid	Burkitt's Lymphoma	Prexasertib + SN-38	1.000	0.057	2.290
910906.000	ST486	Haematopoietic and Lymphoid	Burkitt's Lymphoma	SN-38 + Olaparib	0.702	0.091	2.290
910906.000	ST486	Haematopoietic and Lymphoid	Burkitt's Lymphoma	AZD1775 + SN-38	1.000	0.033	2.290
910906.000	ST486	Haematopoietic and Lymphoid	Burkitt's Lymphoma	SRA737 + SN-38	0.991	0.040	2.290
910907.000	SW1990	Pancreas	Pancreatic Carcinoma	SN-38 + Olaparib	0.406	0.040	4.133
910907.000	SW1990	Pancreas	Pancreatic Carcinoma	AZD1775 + SN-38	0.558	-0.003	4.133
910907.000	SW1990	Pancreas	Pancreatic Carcinoma	Prexasertib + SN-38	0.723	0.032	4.133
910907.000	SW1990	Pancreas	Pancreatic Carcinoma	SRA737 + SN-38	0.519	-0.001	4.133
910907.000	SW1990	Pancreas	Pancreatic Carcinoma	Prexasertib + SN-38	0.759	0.015	4.133
910907.000	SW1990	Pancreas	Pancreatic Carcinoma	Prexasertib + SN-38	0.794	0.007	4.133
910907.000	SW1990	Pancreas	Pancreatic Carcinoma	SN-38 + Olaparib	0.419	0.019	4.133
910907.000	SW1990	Pancreas	Pancreatic Carcinoma	SN-38 + Olaparib	0.449	-0.003	4.133
910910.000	UACC-812	Breast	Breast Carcinoma	Prexasertib + SN-38	0.608	-0.007	5.028
910910.000	UACC-812	Breast	Breast Carcinoma	SRA737 + SN-38	0.472	-0.008	5.028
910910.000	UACC-812	Breast	Breast Carcinoma	AZD1775 + SN-38	0.552	-0.010	5.028
910910.000	UACC-812	Breast	Breast Carcinoma	SN-38 + Olaparib	0.504	0.049	5.028
910910.000	UACC-812	Breast	Breast Carcinoma	SRA737 + SN-38	0.478	0.013	5.028
910910.000	UACC-812	Breast	Breast Carcinoma	AZD1775 + SN-38	0.533	0.013	5.028
910915.000	CAPAN-2	Pancreas	Pancreatic Carcinoma	SN-38 + Olaparib	0.137	-0.024	0.138
910915.000	CAPAN-2	Pancreas	Pancreatic Carcinoma	AZD1775 + SN-38	0.442	-0.010	0.138
910915.000	CAPAN-2	Pancreas	Pancreatic Carcinoma	SRA737 + SN-38	0.250	0.002	0.138
910915.000	CAPAN-2	Pancreas	Pancreatic Carcinoma	Prexasertib + SN-38	0.532	-0.017	0.138
910922.000	769-P	Kidney	Kidney Carcinoma	SN-38 + Olaparib	0.734	0.072	0.401
910922.000	769-P	Kidney	Kidney Carcinoma	SRA737 + SN-38	0.900	0.034	0.401
910922.000	769-P	Kidney	Kidney Carcinoma	Prexasertib + SN-38	0.765	-0.010	0.401
910922.000	769-P	Kidney	Kidney Carcinoma	AZD1775 + SN-38	0.895	0.032	0.401
910924.000	23132-87	Stomach	Gastric Carcinoma	Prexasertib + SN-38	0.905	-0.051	0.070
910924.000	23132-87	Stomach	Gastric Carcinoma	SN-38 + Olaparib	0.814	0.029	0.070
910926.000	BFTC-905	Bladder	Bladder Carcinoma	AZD1775 + SN-38	0.968	0.044	2.858
910926.000	BFTC-905	Bladder	Bladder Carcinoma	SRA737 + SN-38	0.912	-0.010	2.858
910926.000	BFTC-905	Bladder	Bladder Carcinoma	SN-38 + Olaparib	0.974	0.050	2.858
910926.000	BFTC-905	Bladder	Bladder Carcinoma	Prexasertib + SN-38	1.000	0.059	2.858
910927.000	CAL-51	Breast	Breast Carcinoma	Prexasertib + SN-38	0.900	-0.046	0.949
910927.000	CAL-51	Breast	Breast Carcinoma	SN-38 + Olaparib	0.801	0.127	0.949
910933.000	AM-38	Central Nervous System	Glioblastoma	Prexasertib + SN-38	0.866	0.006	6.382
910933.000	AM-38	Central Nervous System	Glioblastoma	SN-38 + Olaparib	0.882	0.023	6.382
910934.000	A4-Fuk	Haematopoietic and Lymphoid	B-Lymphoblastic Leukemia	AZD1775 + SN-38	1.000	-0.004	4.126
910934.000	A4-Fuk	Haematopoietic and Lymphoid	B-Lymphoblastic Leukemia	SRA737 + SN-38	0.974	0.033	4.126
910934.000	A4-Fuk	Haematopoietic and Lymphoid	B-Lymphoblastic Leukemia	SN-38 + Olaparib	0.960	0.038	4.126
910934.000	A4-Fuk	Haematopoietic and Lymphoid	B-Lymphoblastic Leukemia	Prexasertib + SN-38	0.972	0.003	4.126
910937.000	COR-L279	Lung	Small Cell Lung Carcinoma	Prexasertib + SN-38	1.000	0.007	6.961
910937.000	COR-L279	Lung	Small Cell Lung Carcinoma	AZD1775 + SN-38	1.000	-0.022	6.961
910937.000	COR-L279	Lung	Small Cell Lung Carcinoma	SRA737 + SN-38	1.000	0.020	6.961
910937.000	COR-L279	Lung	Small Cell Lung Carcinoma	SRA737 + SN-38	1.000	-0.010	6.961
910937.000	COR-L279	Lung	Small Cell Lung Carcinoma	AZD1775 + SN-38	1.000	0.002	6.961
910937.000	COR-L279	Lung	Small Cell Lung Carcinoma	SN-38 + Olaparib	1.000	0.108	6.961
910943.000	CAS-1	Central Nervous System	Glioblastoma	Prexasertib + SN-38	0.796	0.088	2.163
910943.000	CAS-1	Central Nervous System	Glioblastoma	SN-38 + Olaparib	0.609	-0.001	2.163
910947.000	OCI-AML2	Haematopoietic and Lymphoid	Acute Myeloid Leukemia	Prexasertib + SN-38	1.000	0.020	6.190
910947.000	OCI-AML2	Haematopoietic and Lymphoid	Acute Myeloid Leukemia	SN-38 + Olaparib	0.998	0.092	6.190
910947.000	OCI-AML2	Haematopoietic and Lymphoid	Acute Myeloid Leukemia	SRA737 + SN-38	1.000	-0.007	6.190
910947.000	OCI-AML2	Haematopoietic and Lymphoid	Acute Myeloid Leukemia	AZD1775 + SN-38	1.000	0.004	6.190
910947.000	OCI-AML2	Haematopoietic and Lymphoid	Acute Myeloid Leukemia	Prexasertib + SN-38	1.000	0.019	6.190
910947.000	OCI-AML2	Haematopoietic and Lymphoid	Acute Myeloid Leukemia	SN-38 + Olaparib	1.000	0.105	6.190
910951.000	CML-T1	Haematopoietic and Lymphoid	Chronic Myelogenous Leukemia	SRA737 + SN-38	1.000	0.026	6.655
910951.000	CML-T1	Haematopoietic and Lymphoid	Chronic Myelogenous Leukemia	Prexasertib + SN-38	1.000	0.043	6.655
910951.000	CML-T1	Haematopoietic and Lymphoid	Chronic Myelogenous Leukemia	AZD1775 + SN-38	1.000	0.020	6.655
910951.000	CML-T1	Haematopoietic and Lymphoid	Chronic Myelogenous Leukemia	SN-38 + Olaparib	1.000	0.006	6.655
910952.000	CAL-54	Kidney	Kidney Carcinoma	AZD1775 + SN-38	0.852	0.050	4.180
910952.000	CAL-54	Kidney	Kidney Carcinoma	SN-38 + Olaparib	0.788	0.006	4.180
910952.000	CAL-54	Kidney	Kidney Carcinoma	SRA737 + SN-38	0.793	-0.003	4.180
910952.000	CAL-54	Kidney	Kidney Carcinoma	Prexasertib + SN-38	0.857	-0.048	4.180
911905.000	EFO-21	Ovary	Ovarian Carcinoma	SRA737 + SN-38	0.541	0.000	0.595
911905.000	EFO-21	Ovary	Ovarian Carcinoma	AZD1775 + SN-38	0.612	0.063	0.595
911905.000	EFO-21	Ovary	Ovarian Carcinoma	SN-38 + Olaparib	0.586	0.091	0.595
911905.000	EFO-21	Ovary	Ovarian Carcinoma	Prexasertib + SN-38	0.766	0.021	0.595
917486.000	LS-1034	Large Intestine	Colorectal Carcinoma	SN-38 + Olaparib	0.553	0.062	0.111
917486.000	LS-1034	Large Intestine	Colorectal Carcinoma	Prexasertib + SN-38	0.887	0.116	0.111
917486.000	LS-1034	Large Intestine	Colorectal Carcinoma	AZD1775 + SN-38	0.905	0.106	0.111
917486.000	LS-1034	Large Intestine	Colorectal Carcinoma	SRA737 + SN-38	0.873	0.118	0.111
924100.000	22RV1	Prostate	Prostate Carcinoma	Prexasertib + SN-38	0.699	-0.010	4.804
924100.000	22RV1	Prostate	Prostate Carcinoma	SN-38 + Olaparib	0.688	0.112	4.804
924100.000	22RV1	Prostate	Prostate Carcinoma	Prexasertib + SN-38	0.738	0.007	4.804
924100.000	22RV1	Prostate	Prostate Carcinoma	SRA737 + SN-38	0.685	-0.019	4.804
924100.000	22RV1	Prostate	Prostate Carcinoma	SN-38 + Olaparib	0.707	0.038	4.804
924100.000	22RV1	Prostate	Prostate Carcinoma	AZD1775 + SN-38	0.738	0.033	4.804
924102.000	8505C	Thyroid	Thyroid Gland Carcinoma	Prexasertib + SN-38	0.788	-0.007	5.730

924102.000	8505C	Thyroid	Thyroid Gland Carcinoma	SN-38 + Olaparib	0.849	0.071	5.730
924104.000	B-CPAP	Thyroid	Thyroid Gland Carcinoma	Prexasertib + SN-38	0.935	0.121	3.719
924104.000	B-CPAP	Thyroid	Thyroid Gland Carcinoma	SN-38 + Olaparib	0.946	0.031	3.719
924106.000	CAL-148	Breast	Breast Carcinoma	AZD1775 + SN-38	0.997	0.087	1.287
924106.000	CAL-148	Breast	Breast Carcinoma	SRA737 + SN-38	0.907	-0.049	1.287
924106.000	CAL-148	Breast	Breast Carcinoma	SN-38 + Olaparib	0.960	0.108	1.287
924106.000	CAL-148	Breast	Breast Carcinoma	Prexasertib + SN-38	0.944	-0.029	1.287
924110.000	HDLM-2	Haematopoietic and Lymphoid	Hodgkin's Lymphoma	SRA737 + SN-38	0.724	-0.018	4.469
924110.000	HDLM-2	Haematopoietic and Lymphoid	Hodgkin's Lymphoma	SN-38 + Olaparib	0.499	0.002	4.469
924110.000	HDLM-2	Haematopoietic and Lymphoid	Hodgkin's Lymphoma	Prexasertib + SN-38	0.676	-0.046	4.469
924110.000	HDLM-2	Haematopoietic and Lymphoid	Hodgkin's Lymphoma	AZD1775 + SN-38	0.858	-0.019	4.469
924110.000	HDLM-2	Haematopoietic and Lymphoid	Hodgkin's Lymphoma	Prexasertib + SN-38	0.659	0.010	4.469
924110.000	HDLM-2	Haematopoietic and Lymphoid	Hodgkin's Lymphoma	SN-38 + Olaparib	0.446	-0.045	4.469
924187.000	KLE	Endometrium	Endometrial Carcinoma	SN-38 + Olaparib	0.453	0.052	0.057
924187.000	KLE	Endometrium	Endometrial Carcinoma	Prexasertib + SN-38	0.493	0.095	0.057
924239.000	L-363	Haematopoietic and Lymphoid	Plasma Cell Myeloma	AZD1775 + SN-38	0.957	0.017	0.239
924239.000	L-363	Haematopoietic and Lymphoid	Plasma Cell Myeloma	SRA737 + SN-38	0.878	0.068	0.239
924239.000	L-363	Haematopoietic and Lymphoid	Plasma Cell Myeloma	Prexasertib + SN-38	1.000	0.047	0.239
924239.000	L-363	Haematopoietic and Lymphoid	Plasma Cell Myeloma	SN-38 + Olaparib	0.421	0.033	0.239
924240.000	MDA-MB-415	Breast	Breast Carcinoma	Prexasertib + SN-38	0.568	0.162	0.864
924240.000	MDA-MB-415	Breast	Breast Carcinoma	SRA737 + SN-38	0.431	0.065	0.864
924240.000	MDA-MB-415	Breast	Breast Carcinoma	AZD1775 + SN-38	0.662	0.063	0.864
924240.000	MDA-MB-415	Breast	Breast Carcinoma	SN-38 + Olaparib	0.321	0.036	0.864
924244.000	NCI-H1975	Lung	Lung Adenocarcinoma	SN-38 + Olaparib	0.545	0.032	0.345
924244.000	NCI-H1975	Lung	Lung Adenocarcinoma	Prexasertib + SN-38	0.813	0.130	0.345
925338.000	MDA-MB-157	Breast	Breast Carcinoma	SRA737 + SN-38	0.955	0.083	3.911
925338.000	MDA-MB-157	Breast	Breast Carcinoma	AZD1775 + SN-38	0.975	0.111	3.911
925338.000	MDA-MB-157	Breast	Breast Carcinoma	Prexasertib + SN-38	0.983	0.026	3.911
925338.000	MDA-MB-157	Breast	Breast Carcinoma	SN-38 + Olaparib	0.725	0.016	3.911
925340.000	MKN45	Stomach	Gastric Carcinoma	SN-38 + Olaparib	0.847	-0.036	3.827
925340.000	MKN45	Stomach	Gastric Carcinoma	Prexasertib + SN-38	0.847	0.020	3.827
925340.000	MKN45	Stomach	Gastric Carcinoma	AZD1775 + SN-38	0.894	0.018	3.827
925340.000	MKN45	Stomach	Gastric Carcinoma	SRA737 + SN-38	0.868	0.095	3.827
925341.000	NCI-H810	Lung	Non-Small Cell Lung Carcinoma	Prexasertib + SN-38	0.978	0.144	0.070
925341.000	NCI-H810	Lung	Non-Small Cell Lung Carcinoma	SN-38 + Olaparib	0.557	0.122	0.070
925341.000	NCI-H810	Lung	Non-Small Cell Lung Carcinoma	AZD1775 + SN-38	0.899	0.056	0.070
925341.000	NCI-H810	Lung	Non-Small Cell Lung Carcinoma	SRA737 + SN-38	0.829	0.123	0.070
925346.000	PANC-03-27	Pancreas	Pancreatic Carcinoma	AZD1775 + SN-38	0.899	0.049	4.322
925346.000	PANC-03-27	Pancreas	Pancreatic Carcinoma	SRA737 + SN-38	0.844	0.029	4.322
925346.000	PANC-03-27	Pancreas	Pancreatic Carcinoma	Prexasertib + SN-38	0.957	-0.002	4.322
925346.000	PANC-03-27	Pancreas	Pancreatic Carcinoma	SN-38 + Olaparib	0.839	0.061	4.322
925347.000	PANC-08-13	Pancreas	Pancreatic Carcinoma	SN-38 + Olaparib	0.297	-0.007	0.880
925347.000	PANC-08-13	Pancreas	Pancreatic Carcinoma	Prexasertib + SN-38	0.589	0.115	0.880
925348.000	PANC-10-05	Pancreas	Pancreatic Carcinoma	Prexasertib + SN-38	0.850	0.004	4.571
925348.000	PANC-10-05	Pancreas	Pancreatic Carcinoma	SRA737 + SN-38	0.860	0.018	4.571
925348.000	PANC-10-05	Pancreas	Pancreatic Carcinoma	AZD1775 + SN-38	0.851	0.062	4.571
925348.000	PANC-10-05	Pancreas	Pancreatic Carcinoma	SN-38 + Olaparib	0.760	0.024	4.571
930297.000	SiHa	Cervix	Cervical Carcinoma	Prexasertib + SN-38	0.585	0.081	0.485
930297.000	SiHa	Cervix	Cervical Carcinoma	SN-38 + Olaparib	0.320	0.038	0.485
946353.000	TE-9	Esophagus	Esophageal Squamous Cell Carcinoma	SN-38 + Olaparib	0.458	0.054	4.037
946353.000	TE-9	Esophagus	Esophageal Squamous Cell Carcinoma	Prexasertib + SN-38	0.752	0.039	4.037
946353.000	TE-9	Esophagus	Esophageal Squamous Cell Carcinoma	SRA737 + SN-38	0.589	0.030	4.037
946353.000	TE-9	Esophagus	Esophageal Squamous Cell Carcinoma	AZD1775 + SN-38	0.785	0.030	4.037
946382.000	CAMA-1	Breast	Breast Carcinoma	SRA737 + SN-38	0.393	0.028	0.111
946382.000	CAMA-1	Breast	Breast Carcinoma	Prexasertib + SN-38	0.507	0.089	0.111
946382.000	CAMA-1	Breast	Breast Carcinoma	SN-38 + Olaparib	0.299	0.044	0.111
946382.000	CAMA-1	Breast	Breast Carcinoma	AZD1775 + SN-38	0.572	0.068	0.111
949090.000	Caov-4	Ovary	Ovarian Carcinoma	SN-38 + Olaparib	0.937	0.093	1.057
949090.000	Caov-4	Ovary	Ovarian Carcinoma	Prexasertib + SN-38	1.000	0.026	1.057
949090.000	Caov-4	Ovary	Ovarian Carcinoma	AZD1775 + SN-38	0.918	0.029	1.057
949090.000	Caov-4	Ovary	Ovarian Carcinoma	SRA737 + SN-38	0.900	0.033	1.057
949090.000	Caov-4	Ovary	Ovarian Carcinoma	SN-38 + Olaparib	0.937	0.087	1.057
949090.000	Caov-4	Ovary	Ovarian Carcinoma	Prexasertib + SN-38	1.000	-0.001	1.057
971774.000	RH-18	Soft Tissue	Rhabdomyosarcoma	Prexasertib + SN-38	0.689	-0.020	0.239
971774.000	RH-18	Soft Tissue	Rhabdomyosarcoma	SN-38 + Olaparib	0.760	0.100	0.239
998189.000	LS-180	Large Intestine	Colorectal Carcinoma	AZD1775 + SN-38	0.827	-0.052	0.014
998189.000	LS-180	Large Intestine	Colorectal Carcinoma	SRA737 + SN-38	0.740	0.001	0.014
998189.000	LS-180	Large Intestine	Colorectal Carcinoma	SN-38 + Olaparib	0.748	0.085	0.014
998189.000	LS-180	Large Intestine	Colorectal Carcinoma	Prexasertib + SN-38	0.766	0.020	0.014
1240121.000	BICR22	Head and Neck	Oral Cavity Carcinoma	Prexasertib + SN-38	0.983	-0.015	1.930
1240121.000	BICR22	Head and Neck	Oral Cavity Carcinoma	SN-38 + Olaparib	0.950	0.088	1.930
1240121.000	BICR22	Head and Neck	Oral Cavity Carcinoma	AZD1775 + SN-38	0.919	-0.014	1.930
1240121.000	BICR22	Head and Neck	Oral Cavity Carcinoma	SRA737 + SN-38	0.893	0.001	1.930
1240122.000	BICR78	Head and Neck	Oral Cavity Carcinoma	SN-38 + Olaparib	0.786	0.075	1.000
1240122.000	BICR78	Head and Neck	Oral Cavity Carcinoma	AZD1775 + SN-38	0.876	0.057	1.000
1240122.000	BICR78	Head and Neck	Oral Cavity Carcinoma	SRA737 + SN-38	0.854	0.039	1.000
1240122.000	BICR78	Head and Neck	Oral Cavity Carcinoma	Prexasertib + SN-38	0.812	0.043	1.000
1240123.000	CCK-81	Large Intestine	Colorectal Carcinoma	Prexasertib + SN-38	0.848	-0.086	0.043
1240123.000	CCK-81	Large Intestine	Colorectal Carcinoma	SN-38 + Olaparib	0.587	0.047	0.043
1240123.000	CCK-81	Large Intestine	Colorectal Carcinoma	AZD1775 + SN-38	1.000	0.007	0.043
1240123.000	CCK-81	Large Intestine	Colorectal Carcinoma	SRA737 + SN-38	0.780	-0.003	0.043
1240124.000	CL-40	Large Intestine	Colorectal Carcinoma	SN-38 + Olaparib	0.868	0.086	0.070
1240124.000	CL-40	Large Intestine	Colorectal Carcinoma	Prexasertib + SN-38	0.990	0.077	0.070
1240127.000	EN	Endometrium	Endometrial Carcinoma	AZD1775 + SN-38	0.861	0.063	0.138
1240127.000	EN	Endometrium	Endometrial Carcinoma	SRA737 + SN-38	0.678	0.016	0.138
1240127.000	EN	Endometrium	Endometrial Carcinoma	Prexasertib + SN-38	0.762	0.035	0.138
1240127.000	EN	Endometrium	Endometrial Carcinoma	SN-38 + Olaparib	0.180	-0.020	0.138
1240129.000	FU-OV-1	Ovary	Ovarian Carcinoma	AZD1775 + SN-38	0.871	0.021	0.310
1240129.000	FU-OV-1	Ovary	Ovarian Carcinoma	SRA737 + SN-38	0.495	0.024	0.310
1240142.000	HARA	Lung	Squamous Cell Lung Carcinoma	Prexasertib + SN-38	0.972	0.068	3.647
1240142.000	HARA	Lung	Squamous Cell Lung Carcinoma	AZD1775 + SN-38	0.988	0.038	3.647
1240142.000	HARA	Lung	Squamous Cell Lung Carcinoma	SRA737 + SN-38	0.898	-0.006	3.647
1240142.000	HARA	Lung	Squamous Cell Lung Carcinoma	SN-38 + Olaparib	0.851	0.131	3.647
1240145.000	HCC-44	Lung	Lung Adenocarcinoma	AZD1775 + SN-38	0.935	0.112	0.098
1240145.000	HCC-44	Lung	Lung Adenocarcinoma	SRA737 + SN-38	0.904	0.144	0.098
1240146.000	HCC-827	Lung	Lung Adenocarcinoma	Prexasertib + SN-38	0.786	0.062	2.441
1240146.000	HCC-827	Lung	Lung Adenocarcinoma	SN-38 + Olaparib	0.497	0.060	2.441
1240153.000	IGR-37	Skin	Melanoma	SRA737 + SN-38	0.818	-0.021	5.271
1240153.000	IGR-37	Skin	Melanoma	Prexasertib + SN-38	0.955	-0.020	5.271
1240153.000	IGR-37	Skin	Melanoma	SN-38 + Olaparib	0.863	0.029	5.271
1240153.000	IGR-37	Skin	Melanoma	AZD1775 + SN-38	0.897	0.003	5.271
1240155.000	IM-95	Stomach	Gastric Carcinoma	Prexasertib + SN-38	0.517	0.001	0.356
1240155.000	IM-95	Stomach	Gastric Carcinoma	SN-38 + Olaparib	0.465	0.044	0.356
1240157.000	JHH-2	Liver	Hepatocellular Carcinoma	AZD1775 + SN-38	0.876	0.158	0.084
1240157.000	JHH-2	Liver	Hepatocellular Carcinoma	Prexasertib + SN-38	0.941	0.077	0.084

1240157.000	JHH-2	Liver	Hepatocellular Carcinoma	SN-38 + Olaparib	0.618	-0.015	0.084
1240157.000	JHH-2	Liver	Hepatocellular Carcinoma	SRA737 + SN-38	0.724	0.070	0.084
1240159.000	JHH-6	Liver	Hepatocellular Carcinoma	Prexasertib + SN-38	0.718	0.104	0.029
1240159.000	JHH-6	Liver	Hepatocellular Carcinoma	SN-38 + Olaparib	0.420	0.046	0.029
1240160.000	JHH-7	Liver	Hepatocellular Carcinoma	SRA737 + SN-38	0.616	0.097	0.029
1240160.000	JHH-7	Liver	Hepatocellular Carcinoma	Prexasertib + SN-38	0.784	0.138	0.029
1240160.000	JHH-7	Liver	Hepatocellular Carcinoma	SN-38 + Olaparib	0.290	0.047	0.029
1240160.000	JHH-7	Liver	Hepatocellular Carcinoma	AZD1775 + SN-38	0.855	0.054	0.029
1240166.000	KYM-1	Soft Tissue	Rhabdomyosarcoma	AZD1775 + SN-38	0.960	0.013	5.736
1240166.000	KYM-1	Soft Tissue	Rhabdomyosarcoma	Prexasertib + SN-38	0.984	-0.028	5.736
1240166.000	KYM-1	Soft Tissue	Rhabdomyosarcoma	SN-38 + Olaparib	0.875	0.047	5.736
1240166.000	KYM-1	Soft Tissue	Rhabdomyosarcoma	SRA737 + SN-38	0.945	0.033	5.736
1240172.000	MDA-MB-436	Breast	Breast Carcinoma	Prexasertib + SN-38	0.714	0.061	5.005
1240172.000	MDA-MB-436	Breast	Breast Carcinoma	SN-38 + Olaparib	0.546	0.086	5.005
1240172.000	MDA-MB-436	Breast	Breast Carcinoma	SRA737 + SN-38	0.549	0.021	5.005
1240172.000	MDA-MB-436	Breast	Breast Carcinoma	AZD1775 + SN-38	0.591	0.022	5.005
1240173.000	MDST8	Large Intestine	Colorectal Carcinoma	Prexasertib + SN-38	0.902	-0.091	4.300
1240173.000	MDST8	Large Intestine	Colorectal Carcinoma	SN-38 + Olaparib	0.745	-0.034	4.300
1240174.000	MFE-319	Endometrium	Endometrial Carcinoma	SN-38 + Olaparib	0.301	0.039	0.014
1240174.000	MFE-319	Endometrium	Endometrial Carcinoma	Prexasertib + SN-38	0.854	0.017	0.014
1240179.000	MS751	Cervix	Cervical Carcinoma	Prexasertib + SN-38	0.967	0.102	2.157
1240179.000	MS751	Cervix	Cervical Carcinoma	SN-38 + Olaparib	0.899	0.074	2.157
1240185.000	NCI-H1944	Lung	Lung Adenocarcinoma	Prexasertib + SN-38	0.772	0.025	3.044
1240185.000	NCI-H1944	Lung	Lung Adenocarcinoma	SN-38 + Olaparib	0.634	0.039	3.044
1240187.000	NCI-H2023	Lung	Non-Small Cell Lung Carcinoma	SN-38 + Olaparib	0.857	0.122	5.813
1240187.000	NCI-H2023	Lung	Non-Small Cell Lung Carcinoma	Prexasertib + SN-38	0.909	0.000	5.813
1240187.000	NCI-H2023	Lung	Non-Small Cell Lung Carcinoma	SRA737 + SN-38	0.747	-0.008	5.813
1240187.000	NCI-H2023	Lung	Non-Small Cell Lung Carcinoma	AZD1775 + SN-38	0.802	0.052	5.813
1240189.000	NCI-H211	Lung	Small Cell Lung Carcinoma	AZD1775 + SN-38	1.000	-0.004	0.714
1240189.000	NCI-H211	Lung	Small Cell Lung Carcinoma	SRA737 + SN-38	0.987	-0.044	0.714
1240190.000	NCI-H3122	Lung	Lung Adenocarcinoma	SRA737 + SN-38	0.857	0.000	2.824
1240190.000	NCI-H3122	Lung	Lung Adenocarcinoma	Prexasertib + SN-38	0.933	0.057	2.824
1240190.000	NCI-H3122	Lung	Lung Adenocarcinoma	SN-38 + Olaparib	0.939	0.085	2.824
1240190.000	NCI-H3122	Lung	Lung Adenocarcinoma	AZD1775 + SN-38	0.861	0.036	2.824
1240190.000	NCI-H3122	Lung	Lung Adenocarcinoma	SN-38 + Olaparib	0.925	0.088	2.824
1240190.000	NCI-H3122	Lung	Lung Adenocarcinoma	Prexasertib + SN-38	0.939	0.054	2.824
1240192.000	NCI-H841	Lung	Small Cell Lung Carcinoma	AZD1775 + SN-38	0.918	0.046	0.227
1240192.000	NCI-H841	Lung	Small Cell Lung Carcinoma	Prexasertib + SN-38	0.984	0.014	0.227
1240192.000	NCI-H841	Lung	Small Cell Lung Carcinoma	SN-38 + Olaparib	0.923	0.033	0.227
1240192.000	NCI-H841	Lung	Small Cell Lung Carcinoma	SRA737 + SN-38	0.620	0.069	0.227
1240197.000	OV-90	Ovary	Ovarian Carcinoma	Prexasertib + SN-38	0.575	0.018	2.406
1240197.000	OV-90	Ovary	Ovarian Carcinoma	SRA737 + SN-38	0.519	-0.037	2.406
1240197.000	OV-90	Ovary	Ovarian Carcinoma	SN-38 + Olaparib	0.553	0.087	2.406
1240197.000	OV-90	Ovary	Ovarian Carcinoma	AZD1775 + SN-38	0.631	0.001	2.406
1240198.000	OVISE	Ovary	Ovarian Carcinoma	SN-38 + Olaparib	0.293	0.019	0.465
1240198.000	OVISE	Ovary	Ovarian Carcinoma	Prexasertib + SN-38	0.646	-0.099	0.465
1240199.000	OVKATE	Ovary	Ovarian Carcinoma	AZD1775 + SN-38	0.362	0.021	2.967
1240199.000	OVKATE	Ovary	Ovarian Carcinoma	SRA737 + SN-38	0.138	-0.029	2.967
1240199.000	OVKATE	Ovary	Ovarian Carcinoma	Prexasertib + SN-38	0.314	0.013	2.967
1240199.000	OVKATE	Ovary	Ovarian Carcinoma	SN-38 + Olaparib	0.224	0.009	2.967
1240201.000	PA-TU-8988T	Pancreas	Pancreatic Carcinoma	Prexasertib + SN-38	0.877	0.205	0.333
1240201.000	PA-TU-8988T	Pancreas	Pancreatic Carcinoma	SN-38 + Olaparib	0.604	0.039	0.333
1240212.000	SISO	Cervix	Cervical Carcinoma	SN-38 + Olaparib	0.932	0.064	6.399
1240212.000	SISO	Cervix	Cervical Carcinoma	Prexasertib + SN-38	0.969	0.030	6.399
1240216.000	SNU-182	Liver	Hepatocellular Carcinoma	SN-38 + Olaparib	0.755	0.070	0.124
1240216.000	SNU-182	Liver	Hepatocellular Carcinoma	Prexasertib + SN-38	0.889	0.069	0.124
1240217.000	SNU-398	Liver	Hepatocellular Carcinoma	Prexasertib + SN-38	0.641	0.063	3.223
1240217.000	SNU-398	Liver	Hepatocellular Carcinoma	SN-38 + Olaparib	0.255	0.012	3.223
1240219.000	SUIT-2	Pancreas	Pancreatic Carcinoma	SN-38 + Olaparib	0.728	-0.008	5.785
1240219.000	SUIT-2	Pancreas	Pancreatic Carcinoma	Prexasertib + SN-38	0.750	-0.016	5.785
1290455.000	OCI-AML3	Haematopoietic and Lymphoid	Acute Myeloid Leukemia	SRA737 + SN-38	0.849	-0.031	4.104
1290455.000	OCI-AML3	Haematopoietic and Lymphoid	Acute Myeloid Leukemia	AZD1775 + SN-38	0.981	-0.042	4.104
1290769.000	CL-11	Large Intestine	Colorectal Carcinoma	Prexasertib + SN-38	0.864	0.012	0.057
1290769.000	CL-11	Large Intestine	Colorectal Carcinoma	SN-38 + Olaparib	0.776	0.081	0.057
1290806.000	FU97	Stomach	Gastric Carcinoma	SN-38 + Olaparib	0.277	0.011	0.098
1290806.000	FU97	Stomach	Gastric Carcinoma	Prexasertib + SN-38	0.322	-0.002	0.098
1290806.000	FU97	Stomach	Gastric Carcinoma	SRA737 + SN-38	0.408	-0.049	0.098
1290806.000	FU97	Stomach	Gastric Carcinoma	AZD1775 + SN-38	0.585	0.035	0.098
1290807.000	G-292-Clone-A141B1	Bone	Osteosarcoma	AZD1775 + SN-38	0.515	0.056	0.111
1290807.000	G-292-Clone-A141B1	Bone	Osteosarcoma	Prexasertib + SN-38	0.388	0.039	0.111
1290807.000	G-292-Clone-A141B1	Bone	Osteosarcoma	SN-38 + Olaparib	0.179	-0.019	0.111
1290807.000	G-292-Clone-A141B1	Bone	Osteosarcoma	SRA737 + SN-38	0.410	0.028	0.111
1290908.000	HCC-78	Lung	Lung Adenocarcinoma	SN-38 + Olaparib	0.898	0.019	4.981
1290908.000	HCC-78	Lung	Lung Adenocarcinoma	Prexasertib + SN-38	0.947	-0.018	4.981
1290908.000	HCC-78	Lung	Lung Adenocarcinoma	SRA737 + SN-38	0.959	-0.020	4.981
1290908.000	HCC-78	Lung	Lung Adenocarcinoma	AZD1775 + SN-38	0.954	0.032	4.981
1290922.000	HDQ-P1	Breast	Breast Carcinoma	SN-38 + Olaparib	0.702	0.044	0.516
1290922.000	HDQ-P1	Breast	Breast Carcinoma	Prexasertib + SN-38	0.901	0.099	0.516
1295740.000	ALL-SIL	Haematopoietic and Lymphoid	T-Lymphoblastic Leukemia	SN-38 + Olaparib	1.000	0.090	6.646
1295740.000	ALL-SIL	Haematopoietic and Lymphoid	T-Lymphoblastic Leukemia	Prexasertib + SN-38	1.000	-0.010	6.646
1295741.000	AMO-1	Haematopoietic and Lymphoid	Plasma Cell Myeloma	SRA737 + SN-38	0.967	0.006	0.310
1295741.000	AMO-1	Haematopoietic and Lymphoid	Plasma Cell Myeloma	AZD1775 + SN-38	1.000	-0.005	0.310
1295741.000	AMO-1	Haematopoietic and Lymphoid	Plasma Cell Myeloma	Prexasertib + SN-38	0.986	-0.004	0.310
1295741.000	AMO-1	Haematopoietic and Lymphoid	Plasma Cell Myeloma	SN-38 + Olaparib	0.952	0.009	0.310
1297439.000	COR-L95	Lung	Small Cell Lung Carcinoma	AZD1775 + SN-38	0.643	-0.016	5.842
1297439.000	COR-L95	Lung	Small Cell Lung Carcinoma	SN-38 + Olaparib	0.850	0.046	5.842
1297439.000	COR-L95	Lung	Small Cell Lung Carcinoma	Prexasertib + SN-38	0.882	-0.085	5.842
1297439.000	COR-L95	Lung	Small Cell Lung Carcinoma	SRA737 + SN-38	0.572	0.011	5.842
1297446.000	DND-41	Haematopoietic and Lymphoid	T-Lymphoblastic Leukemia	Prexasertib + SN-38	0.939	-0.058	6.025
1297446.000	DND-41	Haematopoietic and Lymphoid	T-Lymphoblastic Leukemia	AZD1775 + SN-38	0.986	-0.012	6.025
1297446.000	DND-41	Haematopoietic and Lymphoid	T-Lymphoblastic Leukemia	SN-38 + Olaparib	0.947	0.048	6.025
1297446.000	DND-41	Haematopoietic and Lymphoid	T-Lymphoblastic Leukemia	SRA737 + SN-38	0.974	-0.026	6.025
1297447.000	EJM	Haematopoietic and Lymphoid	Plasma Cell Myeloma	Prexasertib + SN-38	0.954	-0.041	3.690
1297447.000	EJM	Haematopoietic and Lymphoid	Plasma Cell Myeloma	SN-38 + Olaparib	0.944	0.042	3.690
1298134.000	HeLa	Cervix	Cervical Carcinoma	SN-38 + Olaparib	0.397	0.033	0.740
1298134.000	HeLa	Cervix	Cervical Carcinoma	Prexasertib + SN-38	0.900	0.113	0.740
1298136.000	HPAC	Pancreas	Pancreatic Carcinoma	SN-38 + Olaparib	0.246	0.002	0.176
1298136.000	HPAC	Pancreas	Pancreatic Carcinoma	Prexasertib + SN-38	0.804	0.046	0.176
1298141.000	Hs-766T	Pancreas	Pancreatic Carcinoma	AZD1775 + SN-38	0.639	0.048	0.151
1298141.000	Hs-766T	Pancreas	Pancreatic Carcinoma	SN-38 + Olaparib	0.415	0.016	0.151
1298141.000	Hs-766T	Pancreas	Pancreatic Carcinoma	Prexasertib + SN-38	0.702	0.017	0.151
1298141.000	Hs-766T	Pancreas	Pancreatic Carcinoma	SRA737 + SN-38	0.631	0.063	0.151
1298146.000	huH-1	Liver	Hepatocellular Carcinoma	SN-38 + Olaparib	0.795	0.060	5.409
1298146.000	huH-1	Liver	Hepatocellular Carcinoma	Prexasertib + SN-38	0.833	0.062	5.409
1298151.000	JHH-1	Liver	Hepatocellular Carcinoma	Prexasertib + SN-38	0.562	-0.053	0.275

1298151.000	JHH-1	Liver	Hepatocellular Carcinoma	SN-38 + Olaparib	0.539	0.019	0.275
1298157.000	JIMT-1	Breast	Breast Carcinoma	Prexasertib + SN-38	0.869	0.025	1.350
1298157.000	JIMT-1	Breast	Breast Carcinoma	SN-38 + Olaparib	0.267	0.039	1.350
1298168.000	KMRC-1	Kidney	Kidney Carcinoma	SN-38 + Olaparib	0.318	0.060	4.039
1298168.000	KMRC-1	Kidney	Kidney Carcinoma	AZD1775 + SN-38	0.564	0.038	4.039
1298168.000	KMRC-1	Kidney	Kidney Carcinoma	Prexasertib + SN-38	0.464	-0.013	4.039
1298168.000	KMRC-1	Kidney	Kidney Carcinoma	SRA737 + SN-38	0.525	0.013	4.039
1298218.000	KP-2	Pancreas	Pancreatic Carcinoma	Prexasertib + SN-38	0.756	0.135	0.111
1298218.000	KP-2	Pancreas	Pancreatic Carcinoma	SN-38 + Olaparib	0.550	0.061	0.111
1298219.000	KP-3	Pancreas	Pancreatic Carcinoma	SRA737 + SN-38	0.273	0.007	0.310
1298219.000	KP-3	Pancreas	Pancreatic Carcinoma	AZD1775 + SN-38	0.538	-0.001	0.310
1298219.000	KP-3	Pancreas	Pancreatic Carcinoma	Prexasertib + SN-38	0.611	0.047	0.310
1298219.000	KP-3	Pancreas	Pancreatic Carcinoma	SN-38 + Olaparib	0.079	-0.024	0.310
1298226.000	LOU-NH91	Lung	Squamous Cell Lung Carcinoma	SN-38 + Olaparib	0.883	0.026	4.598
1298226.000	LOU-NH91	Lung	Squamous Cell Lung Carcinoma	Prexasertib + SN-38	0.894	-0.039	4.598
1298347.000	NCI-H1435	Lung	Non-Small Cell Lung Carcinoma	Prexasertib + SN-38	0.508	0.016	3.360
1298347.000	NCI-H1435	Lung	Non-Small Cell Lung Carcinoma	SN-38 + Olaparib	0.326	0.029	3.360
1298347.000	NCI-H1435	Lung	Non-Small Cell Lung Carcinoma	AZD1775 + SN-38	0.590	0.050	3.360
1298347.000	NCI-H1435	Lung	Non-Small Cell Lung Carcinoma	SRA737 + SN-38	0.460	-0.009	3.360
1298350.000	NCI-H1781	Lung	Lung Adenocarcinoma	Prexasertib + SN-38	0.998	0.019	6.224
1298350.000	NCI-H1781	Lung	Lung Adenocarcinoma	SN-38 + Olaparib	1.000	0.107	6.224
1298358.000	OCUM-1	Stomach	Gastric Carcinoma	SRA737 + SN-38	0.695	-0.048	3.136
1298358.000	OCUM-1	Stomach	Gastric Carcinoma	SN-38 + Olaparib	0.645	0.037	3.136
1298358.000	OCUM-1	Stomach	Gastric Carcinoma	AZD1775 + SN-38	0.650	0.021	3.136
1298358.000	OCUM-1	Stomach	Gastric Carcinoma	Prexasertib + SN-38	0.798	-0.043	3.136
1298359.000	OE21	Esophagus	Esophageal Squamous Cell Carcinoma	SN-38 + Olaparib	0.669	-0.003	1.104
1298359.000	OE21	Esophagus	Esophageal Squamous Cell Carcinoma	Prexasertib + SN-38	0.735	-0.007	1.104
1298475.000	PANC-02-03	Pancreas	Pancreatic Carcinoma	AZD1775 + SN-38	0.726	0.082	0.227
1298475.000	PANC-02-03	Pancreas	Pancreatic Carcinoma	SN-38 + Olaparib	0.728	0.074	0.227
1298475.000	PANC-02-03	Pancreas	Pancreatic Carcinoma	Prexasertib + SN-38	0.741	0.069	0.227
1298475.000	PANC-02-03	Pancreas	Pancreatic Carcinoma	SRA737 + SN-38	0.674	-0.016	0.227
1298476.000	PANC-04-03	Pancreas	Pancreatic Carcinoma	SN-38 + Olaparib	0.465	0.037	2.864
1298476.000	PANC-04-03	Pancreas	Pancreatic Carcinoma	AZD1775 + SN-38	0.617	0.039	2.864
1298476.000	PANC-04-03	Pancreas	Pancreatic Carcinoma	Prexasertib + SN-38	0.651	-0.017	2.864
1298476.000	PANC-04-03	Pancreas	Pancreatic Carcinoma	SRA737 + SN-38	0.570	-0.007	2.864
1298526.000	PA-TU-8902	Pancreas	Pancreatic Carcinoma	Prexasertib + SN-38	0.709	0.078	0.000
1298526.000	PA-TU-8902	Pancreas	Pancreatic Carcinoma	SN-38 + Olaparib	0.428	0.095	0.000
1298538.000	RERF-LC-Sq1	Lung	Squamous Cell Lung Carcinoma	SN-38 + Olaparib	0.672	0.057	5.111
1298538.000	RERF-LC-Sq1	Lung	Squamous Cell Lung Carcinoma	Prexasertib + SN-38	0.896	0.023	5.111
1298539.000	RKN	Soft Tissue	Other Solid Carcinomas	SN-38 + Olaparib	0.651	0.032	2.189
1298539.000	RKN	Soft Tissue	Other Solid Carcinomas	AZD1775 + SN-38	0.959	0.126	2.189
1298539.000	RKN	Soft Tissue	Other Solid Carcinomas	Prexasertib + SN-38	0.970	0.179	2.189
1298539.000	RKN	Soft Tissue	Other Solid Carcinomas	SRA737 + SN-38	0.858	0.138	2.189
1299062.000	SW1271	Lung	Small Cell Lung Carcinoma	SN-38 + Olaparib	0.758	0.142	4.204
1299062.000	SW1271	Lung	Small Cell Lung Carcinoma	SRA737 + SN-38	0.607	0.007	4.204
1299062.000	SW1271	Lung	Small Cell Lung Carcinoma	AZD1775 + SN-38	0.654	0.051	4.204
1299062.000	SW1271	Lung	Small Cell Lung Carcinoma	Prexasertib + SN-38	0.793	-0.044	4.204
1299064.000	T-T	Esophagus	Esophageal Squamous Cell Carcinoma	AZD1775 + SN-38	0.940	0.053	0.251
1299064.000	T-T	Esophagus	Esophageal Squamous Cell Carcinoma	Prexasertib + SN-38	0.977	0.152	0.251
1299064.000	T-T	Esophagus	Esophageal Squamous Cell Carcinoma	SN-38 + Olaparib	0.835	-0.014	0.251
1299064.000	T-T	Esophagus	Esophageal Squamous Cell Carcinoma	SRA737 + SN-38	0.834	0.070	0.251
1299070.000	TOV-112D	Ovary	Ovarian Carcinoma	SN-38 + Olaparib	0.965	0.057	5.606
1299070.000	TOV-112D	Ovary	Ovarian Carcinoma	Prexasertib + SN-38	0.975	0.065	5.606
1299075.000	VCaP	Prostate	Prostate Carcinoma	AZD1775 + SN-38	0.695	-0.040	0.138
1299075.000	VCaP	Prostate	Prostate Carcinoma	SN-38 + Olaparib	0.459	0.122	0.138
1299075.000	VCaP	Prostate	Prostate Carcinoma	SRA737 + SN-38	0.590	0.003	0.138
1299075.000	VCaP	Prostate	Prostate Carcinoma	Prexasertib + SN-38	0.755	0.025	0.138
1303900.000	HCC1500	Breast	Breast Carcinoma	Prexasertib + SN-38	0.673	0.016	0.070
1303900.000	HCC1500	Breast	Breast Carcinoma	SN-38 + Olaparib	0.873	0.057	0.070
1327766.000	JJN-3	Haematopoietic and Lymphoid	Plasma Cell Myeloma	SN-38 + Olaparib	0.688	0.031	0.227
1327766.000	JJN-3	Haematopoietic and Lymphoid	Plasma Cell Myeloma	Prexasertib + SN-38	0.969	0.044	0.227
1327771.000	JURL-MK1	Haematopoietic and Lymphoid	Chronic Myelogenous Leukemia	Prexasertib + SN-38	1.000	0.014	6.359
1327771.000	JURL-MK1	Haematopoietic and Lymphoid	Chronic Myelogenous Leukemia	SN-38 + Olaparib	1.000	0.057	6.359
1327775.000	KARPAS-620	Haematopoietic and Lymphoid	Plasma Cell Myeloma	AZD1775 + SN-38	0.839	-0.003	0.740
1327775.000	KARPAS-620	Haematopoietic and Lymphoid	Plasma Cell Myeloma	SRA737 + SN-38	0.658	0.076	0.740
1327775.000	KARPAS-620	Haematopoietic and Lymphoid	Plasma Cell Myeloma	SN-38 + Olaparib	0.250	-0.015	0.740
1327775.000	KARPAS-620	Haematopoietic and Lymphoid	Plasma Cell Myeloma	Prexasertib + SN-38	0.874	0.035	0.740
1330931.000	KCL-22	Haematopoietic and Lymphoid	Chronic Myelogenous Leukemia	Prexasertib + SN-38	1.000	0.011	0.536
1330931.000	KCL-22	Haematopoietic and Lymphoid	Chronic Myelogenous Leukemia	SN-38 + Olaparib	0.887	0.060	0.536
1330933.000	KOPN-8	Haematopoietic and Lymphoid	B-Lymphoblastic Leukemia	SN-38 + Olaparib	0.965	0.050	5.733
1330933.000	KOPN-8	Haematopoietic and Lymphoid	B-Lymphoblastic Leukemia	Prexasertib + SN-38	1.000	-0.007	5.733
1330935.000	L-1236	Haematopoietic and Lymphoid	Hodgkin's Lymphoma	Prexasertib + SN-38	0.883	0.008	3.990
1330935.000	L-1236	Haematopoietic and Lymphoid	Hodgkin's Lymphoma	SN-38 + Olaparib	0.870	0.031	3.990
1330935.000	L-1236	Haematopoietic and Lymphoid	Hodgkin's Lymphoma	AZD1775 + SN-38	0.982	0.030	3.990
1330935.000	L-1236	Haematopoietic and Lymphoid	Hodgkin's Lymphoma	SRA737 + SN-38	0.944	0.052	3.990
1330942.000	ME-1	Haematopoietic and Lymphoid	Acute Myeloid Leukemia	Prexasertib + SN-38	0.633	-0.012	5.290
1330942.000	ME-1	Haematopoietic and Lymphoid	Acute Myeloid Leukemia	SN-38 + Olaparib	0.497	0.016	5.290
1330942.000	ME-1	Haematopoietic and Lymphoid	Acute Myeloid Leukemia	SRA737 + SN-38	0.675	0.068	5.290
1330942.000	ME-1	Haematopoietic and Lymphoid	Acute Myeloid Leukemia	AZD1775 + SN-38	0.700	0.019	5.290
1330950.000	MOLP-8	Haematopoietic and Lymphoid	Plasma Cell Myeloma	Prexasertib + SN-38	0.870	-0.060	4.631
1330950.000	MOLP-8	Haematopoietic and Lymphoid	Plasma Cell Myeloma	SN-38 + Olaparib	0.783	0.052	4.631
1330964.000	NCI-H1341	Lung	Small Cell Lung Carcinoma	Prexasertib + SN-38	0.973	0.007	5.514
1330964.000	NCI-H1341	Lung	Small Cell Lung Carcinoma	SN-38 + Olaparib	0.990	0.084	5.514
1330973.000	NCI-H2066	Lung	Squamous Cell Lung Carcinoma	AZD1775 + SN-38	0.708	-0.027	0.043
1330973.000	NCI-H2066	Lung	Squamous Cell Lung Carcinoma	SRA737 + SN-38	0.345	0.002	0.043
1330973.000	NCI-H2066	Lung	Squamous Cell Lung Carcinoma	Prexasertib + SN-38	0.448	0.016	0.043
1330973.000	NCI-H2066	Lung	Squamous Cell Lung Carcinoma	SN-38 + Olaparib	0.096	-0.029	0.043
1331036.000	SU-DHL-5	Haematopoietic and Lymphoid	B-Cell Non-Hodgkin's Lymphoma	SN-38 + Olaparib	1.000	0.076	5.753
1331036.000	SU-DHL-5	Haematopoietic and Lymphoid	B-Cell Non-Hodgkin's Lymphoma	Prexasertib + SN-38	1.000	0.006	5.753
1331037.000	SU-DHL-6	Haematopoietic and Lymphoid	B-Cell Non-Hodgkin's Lymphoma	SRA737 + SN-38	1.000	0.038	0.748
1331037.000	SU-DHL-6	Haematopoietic and Lymphoid	B-Cell Non-Hodgkin's Lymphoma	SN-38 + Olaparib	1.000	0.054	0.748
1331037.000	SU-DHL-6	Haematopoietic and Lymphoid	B-Cell Non-Hodgkin's Lymphoma	Prexasertib + SN-38	0.995	0.168	0.748
1331037.000	SU-DHL-6	Haematopoietic and Lymphoid	B-Cell Non-Hodgkin's Lymphoma	AZD1775 + SN-38	1.000	0.044	0.748
1331040.000	SUP-M2	Haematopoietic and Lymphoid	T-Cell Non-Hodgkin's Lymphoma	Prexasertib + SN-38	1.000	0.006	5.118
1331040.000	SUP-M2	Haematopoietic and Lymphoid	T-Cell Non-Hodgkin's Lymphoma	SN-38 + Olaparib	1.000	0.070	5.118
1331050.000	WSU-DLCL2	Haematopoietic and Lymphoid	B-Cell Non-Hodgkin's Lymphoma	SN-38 + Olaparib	1.000	0.062	0.379
1331050.000	WSU-DLCL2	Haematopoietic and Lymphoid	B-Cell Non-Hodgkin's Lymphoma	AZD1775 + SN-38	1.000	0.017	0.379
1331050.000	WSU-DLCL2	Haematopoietic and Lymphoid	B-Cell Non-Hodgkin's Lymphoma	SRA737 + SN-38	1.000	0.014	0.379
1331050.000	WSU-DLCL2	Haematopoietic and Lymphoid	B-Cell Non-Hodgkin's Lymphoma	Prexasertib + SN-38	1.000	0.003	0.379
1503361.000	FLO-1	Esophagus	Esophageal Adenocarcinoma	AZD1775 + SN-38	0.974	0.100	0.138
1503361.000	FLO-1	Esophagus	Esophageal Adenocarcinoma	SRA737 + SN-38	0.815	0.099	0.138
1503361.000	FLO-1	Esophagus	Esophageal Adenocarcinoma	Prexasertib + SN-38	0.931	0.056	0.138
1503361.000	FLO-1	Esophagus	Esophageal Adenocarcinoma	SN-38 + Olaparib	0.479	0.058	0.138
1503363.000	OACM5-1	Esophagus	Esophageal Adenocarcinoma	SN-38 + Olaparib	0.796	0.060	4.274
1503363.000	OACM5-1	Esophagus	Esophageal Adenocarcinoma	Prexasertib + SN-38	0.831	-0.001	4.274

1503365.000	SK-GT-4	Esophagus	Esophageal Adenocarcinoma	SRA737 + SN-38	0.550	-0.004	0.356
1503365.000	SK-GT-4	Esophagus	Esophageal Adenocarcinoma	Prexasertib + SN-38	0.758	-0.015	0.356
1503365.000	SK-GT-4	Esophagus	Esophageal Adenocarcinoma	SN-38 + Olaparib	0.400	0.039	0.356
1503365.000	SK-GT-4	Esophagus	Esophageal Adenocarcinoma	AZD1775 + SN-38	0.522	0.063	0.356
1503366.000	ESO26	Esophagus	Esophageal Adenocarcinoma	Prexasertib + SN-38	0.653	0.113	1.220
1503366.000	ESO26	Esophagus	Esophageal Adenocarcinoma	SN-38 + Olaparib	0.764	0.051	1.220
1503367.000	ESO51	Esophagus	Esophageal Adenocarcinoma	Prexasertib + SN-38	0.826	-0.017	0.014
1503367.000	ESO51	Esophagus	Esophageal Adenocarcinoma	SN-38 + Olaparib	0.624	-0.015	0.014
1503371.000	TE-4	Esophagus	Esophageal Squamous Cell Carcinoma	SN-38 + Olaparib	0.402	0.001	2.789
1503371.000	TE-4	Esophagus	Esophageal Squamous Cell Carcinoma	Prexasertib + SN-38	0.861	0.144	2.789
1659928.000	SNU-175	Large Intestine	Colorectal Carcinoma	SN-38 + Olaparib	0.471	0.039	0.029
1659928.000	SNU-175	Large Intestine	Colorectal Carcinoma	Prexasertib + SN-38	0.603	0.000	0.029

N/A, not applicable

Table S2: Antibodies and the assays in which they were used.

Target	Manufacturer	Catalogue number	Application	Dilution
Alexa Flour 488 anti-rabbit IgG	ThermoFisher	A-11008	IF	1_1000
Alexa-flour 555 anti-mouse IgG	ThermoFisher	A-31570	IF	1_1000
Anti-mouse IgG, HRP conjugated	CST	7076	WB	1_1000
Anti-rabbit IgG, HRP conjugated	CST	7074	WB	1_1000
ATM	Abcam	ab78	WB	1_2000
ATR	CST	2790	WB	1_500
CHK1 (2G1D5)	CST	2360	WB	1_1000
DNA-PKcs (3H6)	CST	12311	WB	1_1000
EGFR (D38B1)	CST	4267	WB	1_1000
GAPDH (14C10)	CST	2118	WB	1_2000
Histone H3 (9610)	CST	3638S	WB	1_1000
KAP1	Abcam	ab10483	WB	1_2000
pATM S1981	Millipore	MAB3806	WB	1_1000
pATR T1989	GeneTex	GTX128145	WB	1_500
pCHK1 S296	Abcam	ab79758	WB	1_500
pCHK1 S317	CST	2344	WB	1_500
pCHK1 S345 (133D3)	CST	2348	WB	1_500
pDNA-PKcs S2056	AstraZeneca	N/A	WB	1_2000
pH2Ax S139 (JBW301)	Millipore	05-636	WB, IF	1_1000, 1_1000
pKAP1 S824	Abcam	ab70369	WB	1_1000
pRPA S33	Bethyl laboratories	A300-246-A	WB	1_1000
pRPA S4/8	Bethyl laboratories	A300-245-A	WB, IF	1_1000, 1_2000
RPA (9H8)	Abcam	ab2175	WB	1_2000
SLFN11	Abcam	ab121731	WB, IF, IHC	1_1000, 1_500, 0.5 µg/ml
SLFN11	Novus	NBP2-57084	WB	1_1000

Table S3: Cell lines with SLFN11 mutations in GDSC.

Cell lines	cDNA	AA	Classification	Subs.	Trunc.
MOLT-4	c.2656G>A	p.A886T	missense	N/A	N/A
MOLT-4	c.2236C>T	p.Q746*	nonsense	N/A	8
CCRF-CEM	c.2123G>A	p.S708N	missense	N/A	N/A
RS4-11	c.1976_1977delTT	p.F659fs*1	frameshift	N/A	8
DND-41	c.1430G>T	p.R477M	missense	N/A	N/A
SUP-T1	c.1245C>A	p.D415E	missense	N/A	N/A
Jurkat	c.1216C>T	p.R406*	nonsense	N/A	8
PF-382	c.1199-2A>C	N/A	ess_splice	N/A	8
DND-41	c.61G>A	p.V21I	missense	N/A	N/A
BT-474	c.487G>C	p.E163Q	missense	N/A	N/A
CW-2	c.2512C>A	p.L838I	missense	N/A	N/A
HCC2998	c.2452G>T	p.E818*	nonsense	N/A	8
SNU-81	c.2393G>A	p.R798H	missense	N/A	N/A
SNU-81	c.2057G>A	p.S686N	missense	N/A	N/A
HT-29	c.2005G>A	p.E669K	missense	2	N/A
HT55	c.1110T>A	p.S370R	missense	N/A	N/A
SNU-1040	c.938G>A	p.C313Y	missense	N/A	N/A
SNU-175	c.866C>A	p.P289H	missense	N/A	N/A
HCC2998	c.67G>A	p.E23K	missense	N/A	N/A
KYSE-140	c.2671delC	p.Q891fs*>11	frameshift	N/A	8
KYSE-450	c.2671delC	p.Q891fs*>11	frameshift	N/A	8
TE-4	c.2671delC	p.Q891fs*>11	frameshift	N/A	8
KYSE-140	c.996G>A	p.W332*	nonsense	N/A	8
KYSE-140	c.995G>A	p.W332*	nonsense	N/A	8
KYSE-510	c.355C>T	p.R119C	missense	2	N/A
LN-18	c.548C>A	p.A183D	missense	N/A	N/A
CAS-1	c.68A>C	p.E23A	missense	N/A	N/A
ML-2	c.1768C>T	p.R590C	missense	N/A	N/A
KMOE-2	c.1229_1230delAG	p.E410fs*19	frameshift	N/A	8
CTV-1	c.142C>T	p.R48W	missense	N/A	N/A
LAMA-84	c.1198+2T>C	N/A	ess_splice	N/A	8
CML-T1	c.208C>G	p.P70A	missense	N/A	N/A
KALS-1	c.92G>A	p.R31K	missense	N/A	N/A
huH-1	c.1157G>C	p.G386A	missense	1	N/A
Calu-6	c.1990delC	p.H664fs*67	frameshift	N/A	8
NCI-H1573	c.106A>G	p.K36E	missense	N/A	N/A
HARA	c.157T>G	p.L53V	missense	N/A	N/A
SK-N-DZ	c.2065C>T	p.R689W	missense	N/A	N/A
CHP-212	c.1939C>T	p.R647*	nonsense	N/A	8
GOTO	c.1741C>T	p.Q581*	nonsense	N/A	8
BE2-M17	c.1216C>T	p.R406*	nonsense	N/A	8
NCI-H1836	c.1992C>A	p.H664Q	missense	N/A	N/A
NCI-H2171	c.1867G>A	p.E623K	missense	N/A	N/A
NCI-H82	c.1295A>T	p.Q432L	missense	N/A	N/A
DMS-79	c.809T>A	p.L270*	nonsense	N/A	8

COLO-668	c.445T>C	p.F149L	missense	N/A	N/A
COR-L32	c.176G>A	p.G59E	missense	N/A	N/A
MMAC-SF	c.1145A>T	p.Y382F	missense	N/A	N/A
IGR-1	c.355C>T	p.R119C	missense	2	N/A
M14	c.134G>A	p.R45K	missense	N/A	N/A
NCI-SNU-1	c.2377G>A	p.A793T	missense	N/A	N/A
MFE-319	c.1466G>A	p.R489H	missense	2	N/A
EW-18	c.1928_1929insA	p.N644fs*17	frameshift	N/A	8
TC-YIK	c.1474T>C	p.F492L	missense	N/A	N/A
RCH-ACV	c.658A>G	p.T220A	missense	N/A	N/A
NCI-H2172	c.356G>C	p.R119P	missense	2	N/A
HH	c.143G>T	p.R48L	missense	N/A	N/A

Subs, substitutions; trunc., truncations; N/A, not applicable.

Table S4: PDX DDA or non-DDA monotherapy treatment of breast cancer.

	Drug	Dose	Schedule	% TGI
DDA	Cisplatin	2	qw x4	59
	Liposomal doxorubicin	5	q7d x3	84
	Doxorubicin	5	q7d x4	84
	Carboplatin	30	2qw x2	129
	Gemcitabine	80	q3d x4	0
	Carboplatin	30	2qw x2	56
	Cisplatin	2	qw x4	76
	Liposomal doxorubicin	5	q7d x3	103
	Cisplatin	2	qw x4	126
	Cisplatin	3	q7d x8	145
	Cisplatin	2	qw x4	10
	Liposomal doxorubicin	5	q7d x3	40
	Gemcitabine	80	q3d x4	42
	Cisplatin	3	q7d x17	74
	Cisplatin	3	q7d x8	105
	Liposomal doxorubicin	5	q7d x3	106
	Liposomal doxorubicin	5	q7d x8	16
	Cisplatin	3	q7d x8	31
	Cisplatin	2	qw x4	35
	Bendamustine	38	qd x1	112
	Bendamustine	38	qd x2	112
	Carboplatin	30	2qw x2	120
	Cisplatin	3	q7d x4	121
	Liposomal doxorubicin	5	q7d x8	127
	Cisplatin	2	qw x4	161
	Mitomycin C	5	q7d x3	44
	Bendamustine	38	qd x2	114
	Bendamustine	38	qd x1	120
	Cisplatin	5	q7d x3	104
	Doxorubicin	5	q7d x3	50
	Capecitabine	200	qd x38	68
	Carboplatin	30	2qw x2	46
	Cisplatin	6	qw x4	46
	Carboplatin	30	2qw x2	73
	Doxorubicin	3	q7d x3	21
	Doxorubicin	3	q7d x3	78
	Liposomal doxorubicin	3	q7d x3	92
	Mitomycin C	5	q7d x3	116
	Liposomal doxorubicin	3	q7d x3	139
	Carboplatin	30	2qw x2	173
	Gemcitabine	100	q7d x3	88
	Capecitabine	200	qd x21	33
	Cisplatin	5	q7d x3	82
	Cisplatin	5	q7d x3	134
	Gemcitabine	100	q7d x3	0

	Capecitabine	200	qd x21	6
	Gemcitabine	100	q7d x3	24
	Carboplatin	25	q7d x3	24
	Carboplatin	25	q7d x3	44
	Capecitabine	200	qd x21	99
	Etoposide	20	q7d x3	32
	Etoposide	20	q7d x3	34
No DDA	Cetuximab	30	2qw x4	1
	Docetaxel	10	q7d x4	55
	Docetaxel	10	q7d x8	79
	Cetuximab	30	2qw x4	-95
	Cetuximab	30	2qw x4	-3
	Docetaxel	10	q7d x8	92
	Cetuximab	30	2qw x4	20
	Docetaxel	10	q7d x17	56
	Docetaxel	10	q7d x8	90
	Docetaxel	10	q7d x8	84
	Palbociclib	50	qd x28	21
	Cetuximab	30	2qw x4	26
	Docetaxel	10	q7d x8	33
	Vinorelbine	5	q7d x3	21
	Cetuximab	30	2qw x4	53
	Cetuximab	40	2qw x4	60
	Bevacizumab	20	2qw x4	70
	Docetaxel	10	q7d x8	90
	Everolimus	100	qd x28	97
	Nab-paclitaxel	50	q4d x3	116
	Everolimus	15	qd x21	-30
	Docetaxel	20	q7d x3	77
	Eribulin	1	2qw x3	104
	Nab-paclitaxel	50	q7d x3	48
	Bevacizumab	20	2qw x3	102
	Eribulin	1	3qw x1	116
	Nab-paclitaxel	20	q7d x3	82
	Eribulin	1	q2d x3	119
	Palbociclib	70	qd x61	56
	Nab-paclitaxel	1	q7d x3	60
	Vinorelbine	0.13	q7d x3	81
	Eribulin	1	q2d x3	158
	Palbociclib	50	qd x60	-2
	Palbociclib	75	qd x21	51
	Nab-paclitaxel	20	q7d x3	88
	Docetaxel	20	qd x1	135
	Eribulin	1	q2d x3	149
	Fulvestrant	5	3qw x4	-90
	Fulvestrant	3	qw x4	40
	Everolimus	3	qd x7	45

	Palbociclib	75	qd x7	55
	Nab-paclitaxel	20	q7d x3	22
	Everolimus	20	qd x21	103
	Eribulin	1	q2d x3	125
	Eribulin	1	q2d x3	122
	Cetuximab	20	2qw x3	-32

TGI, tumour growth inhibition.

Table S5: Treatment and outcome of breast cancer patients.

	Treatment	Outcome	Duration (months)
No DDA-based	Letrozole	No response	N/A
	Trastuzumab/Vinorelbine	Response	2
	Trastuzumab emtansine	No response	N/A
	Anastrozole	No response	N/A
	Bevacizumab	Response	>24
	Trastuzumab/Vinorelbine	Response	7
	Nab-paclitaxel	Response	5
	Docetaxel	No response	N/A
	Vinorelbine/Bevacizumab	Response	7
	Paclitaxel	Response	6
	Tivantinib	No response	N/A
	Eribulin	No response	N/A
	Eribulin	No response	N/A
	Eribulin	No response	N/A
	Paclitaxel/Bevacizumab	No response	N/A
	Trastuzumab emtansine	No response	N/A
	Eribulin	No response	N/A
	Tamoxifen	Response	6
	Exemestane	Response	10
	Fulvestrant	Response	5
	Nab-paclitaxel	No response	N/A
	Eribulin	No response	N/A
	Paclitaxel	No response	N/A
	Paclitaxel/Bevacizumab	No response	N/A
	Fulvestrant	Response	9
	Letrozole	No response	N/A
	Letrozole/Taselisib	Response	2
	Megestrol	No response	N/A
	Vinorelbine	No response	N/A
	Trastuzumab/Docetaxel	Response	4
	Trastuzumab	Response	13
	Vinorelbine/Trastuzumab /Pertuzumab	No response	N/A
	Eribulin/Bevacizumab	No response	N/A
	Trastuzumab	No response	N/A
	Trastuzumab emtansine	No response	N/A
	Trastuzumab/Lapatinib	Responded	N/A
	Trastuzumab/Vinorelbine	Responded	N/A
	Trastuzumab/Pertuzumab /Paclitaxel	Responded	N/A
	Trastuzumab emtansine	No response	N/A
	Trastuzumab	No response	N/A
	Letrozole	Responded	5
	Letrozole/Palbociclib	Responded	6

		Trastuzumab/Pertuzumab /Paclitaxel	No response	N/A
		Paclitaxel	Response	6
		Paclitaxel/Bevacizumab (low dose)	Response	4
		Letrozole/Fulvestrant /Everolimus	No response	N/A
		Trastuzumab/Tamoxifen	Response	>24
		Anastrozole/Goserelin/ Lapatinib/Trastuzumab	Response	>24
		Trastuzumab/Pertuzumab /Docetaxel	Response	12
		Trastuzumab emtansine	No response	N/A
		Paclitaxel	No response	N/A
		Paclitaxel/Bevacizumab	No response	N/A
		Letrozole	No response	N/A
		Letrozole/Palbociclib	No response	N/A
		Tamoxifen	No response	N/A
		Tamoxifen	No response	N/A
		Fulvestrant/Palbociclib	No response	N/A
		Paclitaxel	Responded	N/A
		Carboplatin/Capecitabine	Response	N/A
		Capecitabine/Lapatinib	Response	6.5
		Cyclophosphamide/Docetaxel	No response	N/A
		Doxorubicin/Cyclophosphamide /Paclitaxel	Response	3
		Capecitabine/Lapatinib	Response	6
		Capecitabine/Bevacizumab	No response	N/A
		Carboplatin/Gemcitabine	No response	N/A
		Doxorubicin/Cyclophosphamide /Paclitaxel	Response	9
		Carboplatin/Gemcitabine /Bevacizumab	Response	8
		Capecitabine/Bevacizumab	Response	7
		5-Fluorouracil/Epirubicin /Cyclophosphamide	No response	N/A
		Carboplatin/Docetaxel	No response	N/A
		Cyclophosphamide/Methotrexate /5-Fluorouracil	Response	9
		Doxorubicin/Cyclophosphamide /Paclitaxel	Response	>24
		Carboplatin/Gemcitabine	No response	N/A
		Capecitabine/Docetaxel	Response	10
		Liposomal doxorubicin	No response	N/A
		Doxorubicin/Cyclophosphamide /Paclitaxel	No response	N/A
		Doxorubicin/Cyclophosphamide	Response	N/A

**DDA-based**	Carboplatin/Paclitaxel/Bevacizumab	Response	N/A
	Carboplatin/Docetaxel	No response	N/A
	Cyclophosphamide/Methotrexate/5-Fluorouracil	Response	5
	Cyclophosphamide/Epirubicin/Paclitaxel	No response	N/A
	Doxorubicin/Cyclophosphamide/Paclitaxel	Response	>24
	Liposomal doxorubicin	No response	N/A
	Capecitabine	No response	N/A
	Cisplatin/Gemcitabine	No response	N/A
	Carboplatin/Docetaxel/Trastuzumab	Responded	4
	Vinorelbine/Trastuzumab/5-Fluorouracil	No response	N/A
	Liposomal doxorubicin	No response	N/A
	Carboplatin/Trastuzumab/Pertuzumab	No response	N/A
	Capecitabine/Lapatinib/Trastuzumab	No response	N/A
	Cyclophosphamide/Methotrexate/5-Fluorouracil/Epirubicin	No response	N/A
	Capecitabine/Docetaxel	No response	N/A
	Gemcitabine/Vinorelbine	Response	5
	Cyclophosphamide/Methotrexate/5-Fluorouracil	No response	N/A
	Capecitabine	Response	3
	Carboplatin/Nab-paclitaxel	Responded	10
	Capecitabine	No response	N/A
	Carboplatin/Gemcitabine	Responded	4
	Trastuzumab/Capecitabine	No response	N/A
	Carboplatin/Docetaxel/Trastuzumab	Responded	N/A
	Cisplatin/Doxorubicin	Responded	4
	5-Fluorouracil/Epirubicin/Cyclophosphamide	No response	N/A
	Carboplatin/Docetaxel	No response	N/A
	Cyclophosphamide/Methotrexate/5-Fluorouracil	Response	9
	Capecitabine/Bevacizumab (low dose)	Response	4
	Gemcitabine/Vinorelbine/Bevacizumab	No response	N/A
	Doxorubicin/Cyclophosphamide/Paclitaxel	Response	N/A
	Doxorubicin/Cyclophosphamide	Response	N/A
	Carboplatin/Paclitaxel	Response	N/A

	Carboplatin/Gemcitabine	Response	5
	Doxorubicin/Cyclophosphamide /Paclitaxel	Response	4
	Doxorubicin/Cyclophosphamide /Paclitaxel	Response	3
	Doxorubicin/Cyclophosphamide /Paclitaxel	Response	8
	Cyclophosphamide /Docetaxel	Response	3
	Carboplatin/Docetaxel/Trastuzumab /Pertuzumab	Response	3
	Carboplatin/Paclitaxel	Response	N/A
	Doxorubicin/Cyclophosphamide	Response	N/A
	Cyclophosphamide/Methotrexate /5-Fluorouracil	No response	N/A
	Cyclophosphamide/Methotrexate /5-Fluorouracil	No response	N/A
	Doxorubicin/Cyclophosphamide	Response	4
	Carboplatin/Paclitaxel	Response	N/A
	Doxorubicin/Cyclophosphamide /Paclitaxel	Response	4
**DDA#**	Carboplatin/Capecitabine	Response	N/A
	Carboplatin/Gemcitabine	No response	N/A
	5-Fluorouracil/Epirubicin /Cyclophosphamide	No response	N/A
	Cyclophosphamide/Methotrexate /5-Fluorouracil	Response	9
	Carboplatin/Gemcitabine	No response	N/A
	Liposomal doxorubicin	No response	N/A
	Doxorubicin/Cyclophosphamide	Response	N/A
	Cyclophosphamide/Methotrexate /5-Fluorouracil	Response	5
	Liposomal doxorubicin	No response	N/A
	Capecitabine	No response	N/A
	Cisplatin/Gemcitabine	No response	N/A
	Liposomal doxorubicin	No response	N/A
	Cyclophosphamide/Methotrexate /5-Fluorouracil/Epirubicin	No response	N/A
	Cyclophosphamide/Methotrexate /5-Fluorouracil	No response	N/A
	Capecitabine	Response	3
	Capecitabine	No response	N/A
	Carboplatin/Gemcitabine	Responded	4
	Cisplatin/Doxorubicin	Responded	4
	5-Fluorouracil/Epirubicin /Cyclophosphamide	No response	N/A
	Cyclophosphamide/Methotrexate	Response	9

	/5-Fluorouracil	Response	[illegible]
	Doxorubicin/Cyclophosphamide	Response	N/A
	Carboplatin/Gemcitabine	Response	5
	Doxorubicin/Cyclophosphamide	Response	N/A
	Cyclophosphamide/Methotrexate /5-Fluorouracil	No response	N/A
	Cyclophosphamide/Methotrexate /5-Fluorouracil	No response	N/A
	Doxorubicin/Cyclophosphamide	Response	4

N/A, not applicable; #, selective DDA monotherapies or DDA combination treatments deriving from the DDA-based data set.
